# Comparison of the Electromyography Activity during Exercises with Stable and Unstable Surfaces: A Systematic Review and Meta-Analysis

**DOI:** 10.3390/sports12040111

**Published:** 2024-04-18

**Authors:** Gabriel de Amorim Batista, Sol Patricia Beltrán, Muana Hiandra Pereira dos Passos, Letícia Bojikian Calixtre, Laís Regina de Holanda Santos, Rodrigo Cappato de Araújo

**Affiliations:** 1 Associated Graduate Program in Physical Education UPE/UFPB, University of Pernambuco, Recife 50100-130, Brazil; sol.patriciabeltran@upe.br (S.P.B.); muana.pereira@upe.br (M.H.P.d.P.); 2 Graduate Program in Rehabilitation and Functional Performance (PPGRDF), University of Pernambuco (UPE–Campus Petrolina), Petrolina 56328-900, Brazil; leticia.calixtre@upe.br (L.B.C.); lais.deholanda@upe.br (L.R.d.H.S.); rodrigo.cappato@upe.br (R.C.d.A.)

**Keywords:** upper extremity, lower extremity, abdominal core, electromyography, resistance training

## Abstract

The effect of electromyographic (EMG) activity on agonist muscles during exercises performed on stable and unstable surfaces remains uncertain. We aimed to review the literature regarding the comparison of the EMG activity of the agonist muscles of exercises performed on stable and unstable surfaces. Eighty-six studies that evaluated the EMG activity of 1783 individuals during exercises for the lower limbs, upper limbs, and core were included. The EMG activities of the pectoralis major (SMD = 0.28 [95% CI 0.09, 0.47]) and triceps brachii muscles (SMD = 0.45 [95% CI 0.25, 0.66]) were significantly increased when the unstable device was added to the exercise. Likewise, the EMG activity of all core muscles showed a significant increase with the unstable surface during the exercises, such as the rectus abdominis (SMD = 0.51 [95% CI 0.37, 0.66]), external oblique (SMD = 0.44 [95% CI 0.28, 0.61]), internal oblique (SMD = 1.04 [95% CI 0.02, 2.07]), erector spinae (SMD = 0.37 [95% CI 0.04, 0.71]), and lumbar multifidus (SMD = 0.35 [95% CI 0.08, 0.61]). However, the lower limb muscles did not show greater EMG activity during the exercise with unstable surfaces compared to the stable surface. In conclusion, unstable conditions increase the EMG activity of some upper limb and core muscles compared to a stable surface.

## 1. Introduction

Instability can be defined as a situation in which a part of the body or the body as a whole has difficulty maintaining a position, which may be related to a lack of joint, muscular, or postural stability [1]. Different instability situations are common in sports situations and activities of daily living. In this sense, athletes and non-athletes have used resistance training with instability, aiming to adapt their neuromuscular system to sudden and unforeseen changes in balance [2,3,4,5,6]. Instability can be induced by devices such as a Swiss ball, Bosu ball, and TRX creating an unstable surface or condition. Training on unstable surfaces has been commonly adopted in geriatric [7,8] contexts and rehabilitation programs due to the possibility of obtaining good neuromuscular activation using low loads [9,10] and improving proprioception and balance stimuli [11,12].

It is speculated that a greater demand for stabilization during exercises on unstable surfaces would increase neuromuscular recruitment due to the need for motor and stabilizer components, consequently increasing muscle activation [12]. Resistance training with unstable devices can be performed for warm-up phases and a lower load of the training program, which would generate benefits for athletes and recreationally active individuals [13]. Some studies demonstrate greater electromyographic (EMG) activity for the agonist muscles during exercises under unstable conditions [14,15,16,17], while other studies do not demonstrate significant differences between stable and unstable conditions [18,19,20]. The results described in the literature are divergent concerning the different muscles evaluated, types of exercises, and instability devices.

Only two systematic reviews have compared the effects of using unstable surfaces on electromyographic activity, and both only evaluated periscapular muscles [21,22]. The first study included 33 studies that evaluated 678 subjects and showed that using unstable surfaces generated a slight increase in upper trapezius activity and a slight decrease in serratus anterior activity. At the same time, no effect was observed in the middle and lower trapezius [21]. The second study evaluated whether different types of instability devices provide different EMG responses in the serratus anterior and upper trapezius muscles. The authors verified that only the suspension straps increase the upper trapezius activity and that any instability device decreases the serratus anterior activity [22].

Health professionals frequently prescribe exercises on unstable surfaces during rehabilitation and physical conditioning programs considering the potential benefits of using this surface type. However, the literature is still uncertain about the EMG activity of the upper and lower limbs and core muscles. Therefore, a systematic review of the literature on the subject is necessary and can contribute to prescribing exercises on unstable and stable surfaces for athletes and non-athletes. This study aimed to systematically review and meta-analyze the literature related to the EMG activity of the agonist muscles between exercises performed on stable and unstable surfaces.

## 2. Materials and Methods

This systematic review was registered prospectively (PROSPERO—CRD42020199935) and followed the recommendations of the Preferred Reporting Items for Systematic Reviews and Meta-Analyses [23].

### 2.1. Data Sources and Searches

The bibliographic search was conducted in the PubMed, Web of Science, Scopus, Cochrane Library, Scielo, and Lilacs databases in March 2024, based on the PICOS approach described in detail in Supplementary Online Material S1. In addition, a manual search was performed in the list of references from similar published systematic reviews and included studies. Three reviewers (GAB, SPB, and MHP) independently evaluated all titles and abstracts through the Rayyan program (https://www.rayyan.ai/), and a fourth reviewer (RCA) was consulted in cases of disagreement. The selection of studies was based on the eligibility criteria adopted in the PICOS strategy. The investigators contacted the authors to request any further information by e-mail or Research Gate when needed.

### 2.2. Study Selection

The studies were included according to the following inclusion criteria: (1) Population: athletes and non-athletic adults from both sexes who were experienced with exercises using instability devices; (2) Interventions: exercises for the upper limbs, lower limbs, or trunk/core using an unstable surface; (3) Comparators: the same exercises performed on a stable basis; (4) Outcomes: EMG amplitude values of the agonist muscles; (5) Study type: cross-sectional studies that compared electromyographic activity during an exercise with and without unstable surfaces. The following exclusion criteria were considered: (a) studies which did not haves the full text available in English; (b) not published in scientific journals; and (c) participants with neurological diseases (e.g., Parkinson’s).

### 2.3. Data Extraction

Characteristics of the sample and details from the data collection and analysis were extracted by three independent investigators, followed by a consensus. The following information was collected from each of the studies: (1) authors and publication year of the article; (2) sociodemographic characteristics: age, sex, and health condition; (3) assessed muscles; (4) types of exercises and surfaces; and (5) summary of results.

In the case of studies with insufficient data or which only presented the results in graphs, the authors were contacted by email or Research Gate to allow their inclusion in the review. Some studies presented mean values and standard errors, making it not possible to include the results in the meta-analysis [24,25,26,27,28,29,30,31,32,33]. Therefore, in some cases, it was necessary to transform the standard error values into standard deviations using Review Manager 5.3 (The Cochrane Collaboration, Copenhagen, Denmark) to include these results in the meta-analysis [24,25,26,27,28,29,30,31,32,33]. The authors were contacted for further information if the paper did not provide enough data. If they did not respond, that study would only be considered for qualitative analysis in the systematic review.

### 2.4. Methodological Quality Assessment

The methodological quality of the studies was based on an adapted version of a standardized quality assessment form for observational studies [34], as recommended in the Cochrane manual for systematic reviews, to assess different aspects of the external and internal validity of studies. It was adapted for this review using modified versions of recent systematic reviews about EMG activity [21,35]. The instrument consisted of nine items regarding external validity (two items) and internal validity (seven items), with scores ranging from 0 (worst quality) to 9 (best quality) points.

### 2.5. Data Analysis

Between-group standardized mean differences (SMD) with 95% confidence intervals (95% CI) were calculated for study comparisons, and meta-analyses were performed by pooling the results of each variable using the Review Manager 5.3.4 program. The SMD used was the effect size known as Hedges’ (adjusted) g. Meta-analyses were conducted where evidence from the studies was statistically homogeneous. The Chi-squared and I^2^ statistics were used to measure the study heterogeneity. Non-significant values in the Chi-squared test (*p* > 0.05) and I^2^ scores of lower than 40% were considered non-significant [36].

Meta-analyses were performed using a random-effects model (inverse variance method) to calculate each variable’s pooled and weighted mean SMD (SMDp) and 95% CI. The meta-analyses were organized considering the agonist muscle, while the subgroups were organized by exercises. A sensitivity analysis was conducted to test the influence of the risk of bias by removing studies that scored less than 3 points on the methodological quality scale from the meta-analyses. In addition, we used the funnel plot to identify studies with possible publication bias, analyzing the subgroups with heterogeneity greater than 60%. Effect sizes were interpreted according to Hopkin’s scale [36] by considering them trivial (<0.20), small (0.20–0.59), moderate (0.60–1.19), or large (≥1.20). Statistical significance was set at *p* < 0.05.

## 3. Results

**Figure 1:**
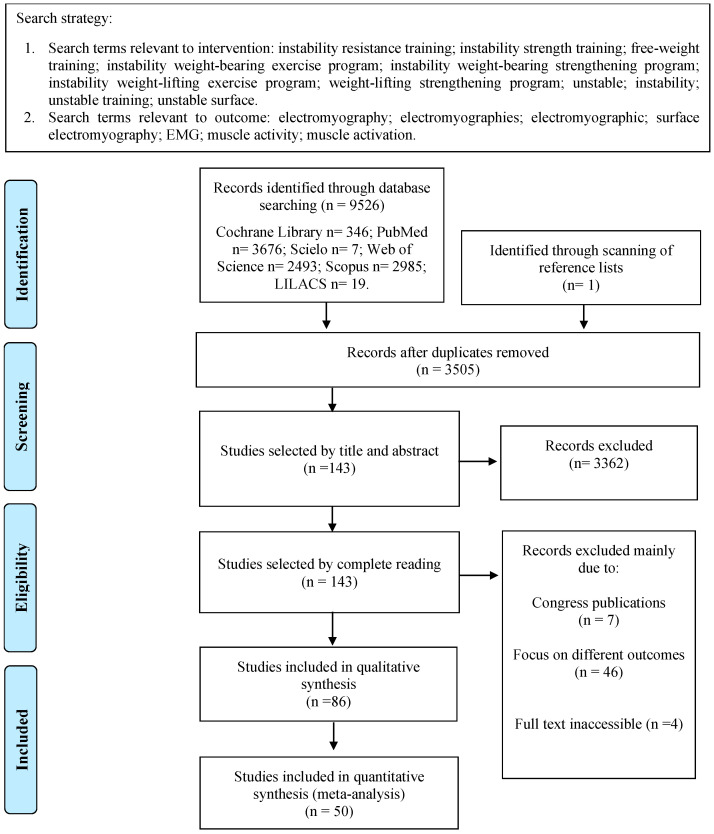
Flow diagram showing the reference screening and study selection.

We included 86 studies; however, only 50 were included for quantitative analysis (Figure 1). Thirty-six studies were not included in the meta-analysis due to the unavailability of the data and the small number of studies that analyzed specific exercises.

### 3.1. Study Characteristics

The characteristics and main results of the selected studies are shown in Supplementary Online Material S1—Tables S1–S3. All studies included in this review were cross-sectional and analyzed the EMG activity of agonist muscles for the upper (36 studies) and lower limbs (27 studies) and core (26 studies) during exercises performed on a stable surface compared to an unstable surface under similar exercise conditions. The following muscles were considered part of the core: rectus abdominis, transversus abdominis, lumbar multifidus, erector spinae, internal oblique, and external oblique [37].

The EMG activity was collected from the primary muscle for each exercise (Supplementary Online Material S1—Table S1); more than one muscle group was assessed in two studies [38,39]. The study conducted by Marshall and Murphy [38] evaluated the agonist muscles of the upper and lower limbs and core. Meanwhile, Aranda et al. [39] evaluated the agonist muscles of the upper and lower limbs. The total sample of this review consisted of 1783 individuals, 494 women and 1289 men. Most studies were composed of healthy individuals, with and without experience in resistance exercises, with a general mean age of 24.0 years, body mass of 72.5 kg, and height of 1.74 m.

A wide variety of exercises were used in the studies; the most common exercises for the upper limbs included push-ups, push-up plus, knee push-ups, knee push-up plus, chest press, bench press, and horizontal dumbbell fly. For the lower limbs, squats, isometric half squats, isometric deep squats, Bulgarian squats, and standard lunges were assessed. The exercises that were investigated for the core muscles were a double-leg bridge, single-leg bridge, side bridge, prone plank, and abdominal crunch. The most unstable devices used during the exercises were the Swiss ball, Bosu ball, wobble board, ball cushion, suspension tape, and foam pad.

Regarding the EMG analysis, 63 studies represented the amplitude using the root mean square (RMS), three studies used Integrated electromyography (iEMG), three studies used the linear envelope, three studies used the average integrated EMG, two studies used Milivolts, two studies using the integral, one study using the absolute integral (mV), one study using the mean peak (mV), one study using the peak electrical signal, and seven studies did not specify.

Normalization varied according to the use of maximum voluntary isometric contraction (MVIC) (40 studies), maximum voluntary contraction (MVC) (17 studies), reference voluntary contraction (RVC) (2 studies), reference voluntary isometric contractions (RVIC) (1 study), 1 RM test (2 studies), peak task activity (1 study), and exercise on a stable surface (1 study). At the same time, 22 did not report this data or did not perform normalization.

### 3.2. Methodological Quality Assessment

**Figure 2:**
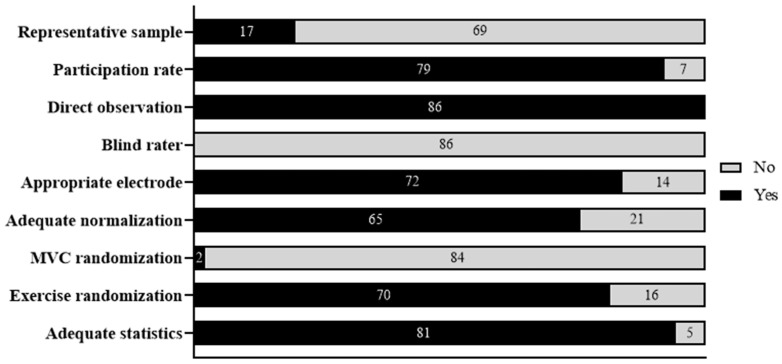
Assessment of methodological quality.

The summary of the methodological quality assessment of the included studies is shown in Figure 2 and Supplementary Online Material S2. Most studies adequately performed statistical analysis (81 studies), a randomization of exercises (70 studies), a normalization of the electromyographic signal (65 studies), and a proper placement of electrodes (72 studies). However, most of the studies did not perform some essential procedures for methodological quality, such as a randomization of the MVIC procedures (84 studies) and a sample size calculation (69 studies).

The mean score of the methodological quality assessment was 5.4/9, ranging from 3 to 8 points. The Supplementary Online Material S3 provides detailed information on each study.

### 3.3. Qualitative Analysis

The following core muscles were assessed by 26 studies: rectus abdominis (23 studies), external oblique (21 studies), internal oblique (7 studies), erector spinae (12 studies), lumbar multifidus (8 studies), and transversus abdominis (2 studies). Greater EMG activity during exercises with the use of unstable surfaces was observed in the muscles rectus abdominis (12 studies), external oblique (9 studies), internal oblique (3 studies), and transversus abdominis (1 study). However, some studies did not verify differences between the EMG activity using unstable and stable surfaces for the erector spinae (9 studies) and lumbar multifidus (6 studies). A summary of the results is presented in Table 1.

The following lower limb muscles were assessed by 27 studies: vastus lateralis (19 studies), vastus medialis (18 studies), biceps femoris (16 studies), rectus femoris (13 studies), gluteus maximus (5 studies), quadriceps (1 study), semitendinosus (1 study), semimembranosus (1 study), hamstring (1 study), and soleus (1 study). Most of the articles did not demonstrate significant differences between the surfaces for the muscles vastus lateralis (13 studies), vastus medialis (11 studies), rectus femoris (9 studies), biceps femoris (13 studies), and semimembranosus (1 study). However, some studies verified greater EMG activity during exercise on unstable surfaces for the gluteus maximus (3 studies), semitendinosus (1 study), hamstring (1 study), soleus (1 study), and quadriceps (1 study).

Finally, the following upper limb muscles were assessed by 36 studies: pectoralis major (29 studies), triceps brachii (25 studies), anterior deltoid (24 studies), biceps brachii (3 studies), latissimus dorsi (2 studies), middle trapezius (2 studies), and posterior deltoid (2 studies). The results demonstrated that the unstable surface was able to generate greater EMG activity for the muscles pectoralis major (11 studies), triceps brachii (13 studies), and anterior deltoid (8 studies). However, most studies did not show differences between the surfaces for latissimus dorsi (1 study), middle trapezius (1 study), posterior deltoid (2 studies), and biceps brachii (2 studies).

### 3.4. Quantitative Analysis

Some studies analyzed more than one type of exercise, so they were included in more than one meta-analysis. A detailed description of the results for each exercise is presented in Supplementary Online Material S1—Tables S1–S3.

Table 2 summarizes the meta-analysis results; the forest plots can be found in Supplementary Online Material S4. When necessary, a sensitivity analysis was performed, removing studies with methodological quality equal to or less than 3 points (Supplementary Online Material S5).

Most studies used body weight to perform the exercises (e.g., push-up, abdominal plank, and isometric squat). However, bench press [2,39,94], chest press [20,38], and squat exercises [5,18,39,66] require external loads with free weights or bars. Thus, the intensity of the exercises that used external loads is available in Table S1 of Supplementary Material S4.

#### 3.4.1. Core

The EMG activity of the rectus abdominis was compared on stable and unstable surfaces during seven types of exercises, demonstrating a small effect size and a significant increase in EMG activity with the insertion of unstable surfaces (SMD = 0.54 [95% CI 0.33, 0.75]; I^2^ = 56%) (Supplementary Online Material S4—Figure S1). An analysis of exercises by subgroups only did not show significant differences for single-leg bridge (SMD = 0.21 [95% CI −0.26, 0.68]) and single-legged hold exercises (SMD = 0.32 [95% CI −1.43, 2.07]). The sensitivity analysis excluding one study with low methodological quality [58] and three studies with potential publication bias [15,45,57] showed small changes, maintaining a small effect size and significantly greater EMG activity in favor of the unstable surface (SMD = 0.51 [95% CI 0.37, 0.66]; I^2^ = 0%) (Supplementary Online Material S5—Figure S1).

The external oblique was assessed during six types of exercises and showed a small effect size and significantly greater EMG activity in favor of the unstable surface (SMD = 0.48 [95% CI 0.29, 0.67]; I^2^ = 39%) (Supplementary Online Material S4—Figure S2). The subgroup analysis demonstrated that only the abdominal crunch exercise did not present a significant difference (SMD = 0.12 [95% CI −0.41, 0.66]). The results of the sensitivity analysis excluding one study due to low methodological quality [58] and one with potential publication bias [46], showed a significantly greater EMG activity in favor of the unstable surface, with a small effect size (SMD = 0.44 [95% CI 0.28, 0.61]; I^2^ = 19%) (Supplementary Online Material S5—Figure S2). 

The internal oblique muscle was assessed during only one type of exercise and showed a moderate effect size and significantly greater EMG activity for an unstable surface (SMD = 1.04 [95% CI 0.02, 2.07]; I^2^ = 74%) (Supplementary Online Material S4—Figure S3). A sensitivity analysis was not performed in this case. 

The erector spinae muscle was analyzed during four types of exercises, showing a significantly greater EMG activity for an unstable surface, with a small effect size (SMD = 0.47 [95% CI 0.18, 0.76]; I^2^ = 53%) (Supplementary Online Material S4—Figure S4). However, the analysis of subgroups shows that only the prone plank with hands exercise showed a significant difference in favor of the unstable surface SMD = 0.76 [95% CI 0.22, 1.30]). A sensitivity analysis that excluded one study with low methodological quality [14] and one subgroup with high heterogeneity showed a similar result, with a small effect size and significance in favor of the unstable surface (SMD = 0.37 [95% CI 0.04, 0.71]; I^2^ = 52%) (Supplementary Online Material S5—Figure S3).

Finally, the lumbar multifidus muscles were analyzed during three exercises and presented significantly greater EMG activity for the unstable surface and with a small effect size (SMD = 0.35 [95% CI 0.08, 0.61]; I^2^ = 32%) (Supplementary Online Material S4—Figure S5). When analyzing the data of the subgroups, only the single-leg bridge exercise did not show differences in the EMG activity between the surfaces (SMD = 0.00 [95% CI −0.47, 0.47]). A sensitivity analysis was not performed in this case.

#### 3.4.2. Upper Limbs

The EMG activity of the pectoralis major muscle was compared between stable and unstable surfaces during seven types of exercises, showing a significantly greater activity under unstable conditions but with a small effect size (SMD = 0.29 [95% CI 0.14, 0.44]; I^2^ = 21%) (Supplementary Online Material S4—Figure S6). Exercise analysis by subgroups only demonstrated significant differences in favor of the unstable surface for push-ups (dynamic), with a small effect size (SMD = 0.43 [95% CI 0.17, 0.68]), and push-ups (concentric phase) with a moderate effect size (SMD = 0.62 [95% CI 0.11, 1.12]). The sensitivity analysis excluding one study with low methodological quality [30] showed a significantly similar result with a small effect size (SMD = 0.28 [95% CI 0.09, 0,47]; I^2^ = 31%) (Supplementary Online Material S5—Figure S4).

The EMG activity of the triceps brachii muscle was assessed during four types of exercises, showing a small effect size and significantly increased muscle activity when an unstable surface was added (SMD = 0.48 [95% CI 0.12, 0.84]; I^2^ = 80%) (Supplementary Online Material S4—Figure S7). The analysis of exercises by subgroups also demonstrated a significant difference in favor of using an unstable surface for push-ups (dynamic), with a moderate effect size (SMD = 0.79 [95% CI 0.31, 1.26]) and push-up exercises (concentric phase) showing a small effect size (SMD = 0.58 [95% CI 0.10, 1.05]). A sensitivity analysis removing three potentially heterogeneous studies [26,27,94] and excluding one study with low methodological quality [30] showed smaller but still significant differences in favor of the unstable surface and with a small effect size (SMD 0.45 [95% CI 0.25, 0.66]; I^2^ = 7%) (Supplementary Online Material S5—Figure S5).

The EMG activity of the anterior deltoid muscle was assessed during four types of exercises. Overall, the subgroup analysis did not demonstrate significant differences between surfaces (SMD = −0.11 [95% CI −0.43, 0.21]; I^2^ = 72%) (Supplementary Online Material S4—Figure S8). A sensitivity analysis removing two potentially heterogeneous studies [26,27] showed similar and non-significant results (SMD = 0.08 [95% CI −0.14, 0.30]; I^2^ = 24%) (Supplementary Online Material S5—Figure S6). 

#### 3.4.3. Lower Limbs

The meta-analysis demonstrated no significant difference in the EMG activity between the surfaces for the muscles of the lower limbs (rectus femoris, vastus lateralis, vastus medialis, and biceps femoris). The rectus femoris muscle was only assessed during one type of exercise and showed no significant differences between the surfaces (SMD = −0.93 [95% CI −2.36, 0.50]; I^2^ = 94%) (Supplementary Online Material S4—Figure S9). After a sensitivity analysis excluding the study with low methodological quality [68], the results remained non-significant (SMD = −0.26 [95% CI −1.01, 0.49]; I^2^ = 61%) (Supplementary Online Material S5—Figure S7). 

Likewise, the vastus lateralis EMG activity showed no significant difference between the surfaces when analyzing four types of exercises (SMD = 0.27 [95% CI −0.17, 0.72]; I^2^ = 85%) (Supplementary Online Material S4—Figure S10). After a sensitivity analysis excluding one study [68] because of the low methodological quality, the results remained non-significant (SMD = 0.03 [95% CI −0.14, 0.21]; I^2^ = 0%) (Supplementary Online Material S5—Figure S8). 

The vastus medialis EMG activity was assessed during four types of exercises and showed no significant differences between the surfaces (SMD = 0.27 [−0.17, 0.72]; I^2^ = 85%) (Supplementary Online Material S4—Figure S11). A sensitivity analysis was carried out excluding one study [61] by analyzing the funnel plot and two studies [68,75] because of the methodological quality, and the results did not demonstrate significant differences (SMD = −0.10 [95% CI −0.40, 0.20]; I^2^ = 43%) (Supplementary Online Material S5—Figure S9). 

The EMG activity of the biceps femoris muscle was assessed in four types of exercises, showing no significant differences between the surfaces (SMD = 0.12 [95% CI −0.09, 0.33]; I^2^ = 0%) (Supplementary Online Material S4—Figure S12). A sensitivity analysis was performed, removing one study with low methodological quality [75] and showed similar results and no significant differences (SMD = 0.09 [95% CI −0.14, 0.32]; I^2^ = 0%) (Supplementary Online Material S5—Figure S10).

## 4. Discussion

This study aimed to systematically review and meta-analyze the literature related to the EMG activity of the agonist muscles between exercises performed on stable and unstable surfaces. Eighty-six cross-sectional studies were included, with an average methodological quality score of 5.4 points. The results of this review demonstrated that the use of an unstable condition was able to generate greater EMG activity for the core agonist muscles and for some muscles of the upper limbs (triceps brachial and pectoralis major) when compared to the same exercise performed using a stable surface. However, the surface did not show significant differences for the lower limb agonist muscles. The results according to the muscle group are discussed below.

### 4.1. Core

The core musculature plays a fundamental role in stabilizing the trunk. Thus, strengthening this musculature is commonly used in rehabilitation processes and aims to improve sports performance and prevent lesions. When analyzing the results of the present study, there is a small effect size in favor of using unstable surfaces for the core muscles. Isometric double-leg bridge, side bridge, and prone plank exercises with or without hand support performed on unstable surfaces can be an excellent strategy to generate greater EMG activity for the rectus abdominis muscle compared to the stable surface [14,45,46]. Furthermore, it is possible to establish a progression using instability devices, or even upper and lower limb supports to increase the complexity of the exercises.

Still considering the rectus abdominis muscle for dynamic exercises, performing abdominal crunches using a Swiss ball may be an option to increase the neuromuscular recruitment of the rectus abdominis muscle, giving preference to the positioning of the unstable surface in the lumbar region [45,52].

A small effect size is also observed in favor of using unstable surfaces for the external oblique muscle (SMD = 0.44 [95% CI 0.28, 0.61]). However, we emphasize that only the abdominal crunch exercise subgroup showed no significant difference when using unstable surfaces. These findings can be explained by considering the function, origin, and insertion of the external oblique muscle, acting in the unilateral contraction and rotation of the trunk [100]. Thus, single-leg bridge, single-legged hold, prone plank, and side bridge exercises on unstable surfaces are the main options for increasing the EMG activity of the external oblique muscle [15,45,46,59].

Only two studies regarding the analysis of the internal oblique muscle were included in the meta-analysis [43,46]. The results showed a moderate effect size in favor of using unstable surfaces during the double-leg bridge exercise (SMD = 1.04 [95% CI 0.02, 2.07]). However, we emphasize that the result of the meta-analysis presents a large confidence interval and high heterogeneity between the studies, which makes it challenging to analyze the effect of the unstable surface on the internal oblique muscle. When looking at the qualitative results in Table 1, we observed that few studies examined this musculature and that most did not demonstrate differences between the surfaces. Thus, professionals can choose to use unstable surfaces to vary the exercises and obtain greater EMG activity in other core muscles.

When analyzing the erector spinae and lumbar multifidus muscle, there was a small effect size and significant effect in favor of using unstable surfaces (SMD = 0.37 [95% CI 0.04, 0.71]) and (SMD = 0.35 [95% CI 0.08, 0.61]), respectively. We verified that the subgroup of exercises that presented the greatest significant difference was the prone plank with hands exercise. Of the two studies included in the meta-analysis [14,46], only using the TRX instability device could generate greater EMG activity, especially when the instability device was placed on the feet [14]. Using the wobble board during an abdominal plank with the hands could not generate greater EMG activity for the erector spinae muscle [46].

Finally, we emphasize that only two studies evaluated the transversus abdominis muscle, demonstrating uncertain results for this musculature. Thus, we suggest that further studies analyze this musculature to verify the effect of adding unstable surfaces.

When comparing our findings with those in the literature, we only found one systematic review that evaluated the EMG activity for the core muscles [37]. The authors analyzed 1247 participants, collecting data on the EMG activity of 233 exercises. The results demonstrate greater activity of the rectus abdominis, external oblique, and erector spinae muscles in exercises with free weights, while the internal oblique muscle is more activated during core stability exercises. Low-load exercises for activation with trunk extension generated the greatest EMG activity for the lumbar multifidus muscle. There is also a scarcity of studies that have analyzed the EMG activity of the transversus abdominis.

Although the review conducted by Oliva-Lozano and Muyor (2020) [37] also contributes to the prescription of exercises for the core region, the authors did not compare the effects between stable and unstable surfaces. Thus, no systematic review has compared the impact of using stable and unstable surfaces for the core musculature. Some studies highlight that strengthening the core can help transfer forces to the lower and upper limbs during daily performance and athletic activities [12,13]. Furthermore, specific strengthening of the trunk muscles is recommended to treat acute and chronic low back pain [101]. Based on the results of the present review, using unstable surfaces can be a complementary strategy to increase the neuromuscular demand during exercises for the core.

### 4.2. Lower Limbs

The addition of unstable surfaces for the agonist muscles of the lower limbs did not show significant differences for the EMG activity of the vastus lateralis (SMD = 0.03 [95% CI −0.14, 0.21]), vastus medialis (SMD = −0.10 [95% CI −0.40, 0.20]), rectus femoris (SMD = −0.26 [95% CI −1.01, 0.49]), and biceps femoris (SMD = 0.09 [95% CI −0.14, 0.32]). No exercise subgroup showed a significant difference, even considering different angles of the squat exercise, with or without external overload and varying the contraction types (isometric or dynamic).

Regarding the dynamic squat exercises performed with external overload, a load variation between 30% and 90% of 1 RM is observed [18,64]. The study conducted by Li, Cao, and Chen (2013) [18] analyzed the effect of the unstable surface during the squat exercise on the electromyographic activity of the vastus lateralis, vastus medialis, rectus femoris, and biceps femoris muscles. The authors used an exercise protocol with body weights of 30% and 60% of 1 RM. The results demonstrated no significant difference between the surfaces [18]. Similarly, McBride et al. [64] verified the same exercise considering the vastus lateralis and biceps femoris muscles with loads between 70% and 90% of 1 RM. The results also demonstrated no significant differences between stable and unstable surfaces [64].

A variation in knee angle between 15° and 100° is observed for the isometric squat exercises [6,38,61,62,65,68,69,71,72,75,78]. The study conducted by Kang et al. [69] analyzed the isometric squat exercise in three angles (15°, 45°, and 60°). The results demonstrated different responses for the vastus medialis muscle, with greater EMG activity being observed for an unstable surface at 15°, while 60° generated greater EMG activity for a stable surface [69]. However, there were no differences between the surfaces for the squat exercise performed at 45° [69].

These values are considered trivial even with significant differences between the surfaces at different angles (15° and 60°), so we must interpret them cautiously, as they may not reflect practical changes. Most of the studies included in this review did not find significant differences between the surfaces during the isometric squat exercise at different angles [6,38,68,75,78].

A possible justification for not observing an increase in the EMG activity of the agonist muscles during lower limb exercises could be related to a flattening of the instability devices, especially inflatable devices (i.e., Balance disc and Bosu ball). A second possibility may be related to the individuals’ experience with strength training. Thus, the subjects could perform the exercises on unstable surfaces without much neuromuscular demand.

Although there are no significant differences between the conditions for the agonist muscles, adding unstable surfaces can increase the EMG activity of the stabilizer muscles during squat exercises [12,13,67]. Furthermore, exercises in unstable conditions for the lower limbs can improve balance and reduce the risk of falls, especially in older adults [7,102]. Strength training with instability improves functional mobility and reduces concerns about falls [7,102].

Therefore, we suggest that training programs use exercises on unstable surfaces, as there are no differences in the EMG activity of the agonist muscles. Thus, the initial stages of training programs can optimize neural adaptations and improve balance by increasing the complexity of exercises performed on unstable surfaces. Furthermore, this type of training can also be used when the objective is to prevent falls and improve functional mobility in older people. However, the exercises must be performed using stable surfaces when the aim is to improve muscular strength and power.

### 4.3. Upper Limbs

The addition of unstable surfaces for the agonist muscles of the upper limbs resulted in greater EMG activity with a small effect size of the pectoralis major (SMD = 0.28 [95% CI 0.09, 0.47]) and triceps brachii (SMD = 0.45 [95% CI 0.25, 0.66]). The subgroups that showed significant differences in favor of unstable surfaces were the dynamic push-up exercises and those with concentric phases. Performing the suspended flexion exercise resulted in greater EMG activity for the pectoralis major and triceps brachii muscles when using unstable surfaces. A possible explanation for these results is the multidirectional instability projected by the suspension tapes [26,27,28].

Loads for the bench press and chest press exercises ranged from 50% to 80% of 1 RM [20,39,93,94], and the studies did not demonstrate differences between surfaces in all muscles included in the meta-analyses. In this case, high loads can result in a flattening of the ball and promote greater stability during exercises. Thus, we believe that loads close to 60% of 1 RM would be the best option for using unstable surfaces when using the Swiss ball during the fly, bench press, and chest press exercises [9,10].

The results for the anterior deltoid muscle demonstrated no differences between the surfaces (SMD = 0.08 [95% CI −0.14, 0.30]). It is difficult to discuss the findings for the other muscles evaluated, since there are few studies, and none of them had quantitative data. The results of the present study contribute to understanding the effect of adding unstable surfaces on the agonist muscles since systematic reviews have focused on the role of the stabilizer muscles [21,22].

Considering the present study’s results, using unstable surfaces during push-up exercises can be an excellent strategy to increase the EMG activation of the pectoralis major and triceps brachii muscles. However, the physical condition of individuals must be considered. The literature shows that performing push-ups on unstable surfaces can increase the EMG activity of the upper trapezius [21], especially in individuals with scapular dyskinesis [103]. Depending on the goal, we may also consider other ways to progress the push-up exercise before introducing unstable surfaces [104].

It was also observed that there are no significant differences between stable and unstable conditions regarding exercises performed with free weights. However, exercises on unstable surfaces for the upper limbs can be used as a pre-activation strategy [105] or as a primary exercise [10] with loads of 30% of 1 RM. Another factor that we must take into account when prescribing exercises is the direction of the loads (axial or rotational) and the distal segment (fixed or mobile) [106]. A study conducted by Nascimento et al. [9] used an unstable surface in exercises with rotational load and this provided superior EMG activity of the agonist muscles. In contrast, instability in exercises with axial load favors the EMG activity of the scapular stabilizing muscles.

Performing exercises on surfaces for the upper limbs can increase the EMG activity of the trunk muscles due to the need for movement control and can provide benefits when training with low loads [12,13]. However, using stable surfaces is recommended if the objective is to gain strength and power in the upper limbs. Finally, health professionals can prescribe training using stable and unstable surfaces considering the objective and conditioning of individuals.

The present study has some strengths: (1) it is the first systematic review with meta-analysis which sought to evaluate the effect of an unstable surface on the EMG activity of the agonist muscles; (2) the search was performed in six databases; (3) blind peer review; (4) an analysis by subgroups of exercises and type of muscle contraction; and (5) sensitivity analysis. However, this review also faced difficulties due to the heterogeneity of the intervention, such as different exercise intensities and devices used for instability by the primary studies. Furthermore, some primary studies did not provide the values necessary for the quantitative synthesis. In some cases, it was not possible to perform sensitivity analysis considering the relative intensity of the exercises due to the small number of studies. The methodological quality of the included studies was low, and the EMG has its limitations related to its low sensitivity. Given the above, we suggest that future studies present the results quantitatively and be careful in the methodological aspects of the study (e.g., sample calculation, randomization of exercises, and CIVM).

Considering the results of the present study, we emphasize that professionals can choose to insert unstable surfaces according to their goals. Using unstable surfaces may be a strategy to increase the recruitment of some agonist muscles of the upper limbs, such as the pectoralis major and the triceps brachii, and for all agonist core muscles. In addition, this type of training can be used to vary stimuli or as a progression during resistance training. However, additional benefits of using unstable surfaces are not seen during lower limb exercises.

## 5. Conclusions

It is concluded that using unstable surfaces generated a slight increase in electromyographic activity, considering the pectoralis major and triceps brachii muscles as agonists of the upper limbs and that the core musculature participated as an agonist in all exercises. However, no effect was observed on lower limb muscles.

## Figures and Tables

**Table 1:** Summary of the qualitative synthesis results.

	Muscle	↑ Unstable Surface	↑ Stable Surface	↔ No Difference between Surfaces	Total Number of Studies
Core	Rectus abdominis	12 studies [14,15,32,38,40,41,42,43,44,45,46,47]	----	11 studies [48,49,50,51,52,53,54,55,56,57,58]	23 studies
	External oblique	9 studies [15,41,43,44,45,46,47,59,60]	2 studies [42,55]	10 studies [32,38,50,51,52,53,54,56,57,58]	21 studies
	Internal oblique	3 studies [43,46,57]	----	4 studies [48,50,54,56]	7 studies
	Erector spinae	3 studies [14,41,59]	----	9 studies [32,40,42,46,47,48,56,58,60]	12 studies
	Lumbar multifidus	1 study [14]	1 study [49]	6 studies [31,46,47,48,50,53]	8 studies
	Transversus abdominis	1 study [54]	----	1 study [54]	2 studies
Lower limbs	Vastus lateralis	4 studies [5,61,62,63]	2 studies [64,65]	13 studies [6,18,33,38,39,66,67,68,69,70,71,72,73]	19 studies
	Vastus medialis	6 studies [5,61,62,63,71,74]	1 study [65]	11 studies [4,6,18,66,68,69,70,72,73,75]	18 studies
	Rectus femoris	3 studies [33,63,74]	1 study [72]	9 studies [4,6,18,66,68,70,76,77,78]	13 studies
	Quadriceps	----	1 study [79]	----	1 study
	Biceps femoris	2 studies [5,74]	1 study [64]	13 studies [4,6,18,33,38,39,65,66,67,72,75,78,80]	16 studies
	Gluteus maximus	3 studies [76,77,80]	----	2 studies [18,74]	5 studies
	Semitendinosus	1 study [6]	----	----	1 study
	Semimembranosus	----	----	1 study [6]	1 study
	Hamstring	1 study [76]	----	----	1 study
	Soleus	1 study [79]	----	----	1 study
Upper limbs	Pectoralis major	11 studies [9,10,16,17,25,26,28,30,81,82,83]	1 study [84]	17 studies [2,3,20,27,38,39,85,86,87,88,89,90,91,92,93,94,95]	29 studies
	Triceps brachii	13 studies [16,17,26,27,28,30,38,81,85,87,90,96,97]	3 studies [84,94,98]	9 studies [2,9,19,24,25,83,86,92,93]	25 studies
	Anterior deltoid	8 studies [9,10,17,24,84,88,93,99]	8 studies [2,26,27,28,82,86,87,90]	8 studies [20,29,39,89,92,94,95,98]	24 studies
	Latissimus dorsi	1 study [82]	----	1 study [19]	2 studies
	Middle trapezius	----	1 study [82]	1 study [19]	2 studies
	Posterior deltoid	----	----	2 studies [19,82]	2 studies
	Biceps brachii	1 study [9]	----	2 studies [2,19]	3 studies

Note: **↔**: No differences in electromyographic activity; ↑: Greater electromyographic activity.

**Table 2:** Summary of the quantitative synthesis results.

	Subgroup Categories	Types of Exercises	Studies	Std. Mean Difference, Random [95% CI]	I-Squared [%]	Std. Mean Difference, Random [95% CI] (Sensitivity Analysis)	I-Squared [%] (Sensitivity Analysis)
Core	Rectus abdominis	7	[14,15,41,43,45,46,48,50,51,52,53,57,58]	**0.54 [0.33, 0.75]**	56%	**0.51 [0.37, 0.66]**	0%
	External oblique	6	[15,41,43,45,46,50,51,52,53,57,58,59]	**0.48 [0.29, 0.67]**	39%	**0.44 [0.28, 0.61]**	19%
	Internal oblique	1	[43,46]	**1.04 [0.02, 2.07]**	74%	Not possible	-
	Erector spinae	4	[14,41,46,48,58,59]	**0.47 [0.18, 0.76]**	53%	**0.37 [0.04, 0.71]**	52%
	Lumbar multifidus	3	[14,46,48,53]	**0.35 [0.08, 0.61]**	32%	Not performed	-
Lower limbs	Vastus lateralis	4	[5,6,18,33,38,39,61,62,66,68,69,71,72,73]	0.27 [−0.17, 0.72]	85%	0.03 [−0.14, 0.21]	0%
	Vastus medialis	4	[5,6,18,61,62,64,68,69,71,72,73,75]	0.20 [−0.35, 0.76]	88%	−0.10 [−0.40, 0.20]	43%
	Rectus femoris	1	[6,68,72]	−0.93 [−2.36, 0.50]	94%	−0.26 [−1.01, 0.49]	61%
	Biceps femoris	4	[5,6,18,33,38,39,64,66,72,75]	0.12 [−0.09, 0.33]	0%	0.09 [−0.14, 0.32]	0%
Upper limbs	Pectoralis major	7	[2,3,17,20,25,26,27,28,30,38,39,81,82,83,85,89,90,94]	**0.29 [0.14, 0.44]**	21%	**0.28 [0.09, 0.47]**	31%
	Triceps brachii	4	[2,17,24,25,26,27,28,30,81,83,85,94,96,97]	**0.48 [0.12, 0.84]**	80%	**0.45 [0.25, 0.66]**	7%
	Anterior deltoid	4	[2,17,20,24,26,27,28,29,38,39,89,90,94]	−0.11 [−0.43, 0.21]	72%	0.08 [−0.14, 0.30]	24%

## Data Availability

The original contributions presented in the study are included in the article/Supplementary Materials; further inquiries can be directed to the corresponding author.

## Supplementary Materials

The following supporting information can be downloaded at: https://www.mdpi.com/article/10.3390/sports12040111/s1, Supplementary Online Material S1: Study Characteristics—Table S1: Summary of the characteristics and results of the selected studies to core muscles, Table S2: Summary of the characteristics and results of the selected studies to lower limbs muscles, Table S3: Summary of the characteristics and results of the selected studies to upper limbs muscles; Supplementary Online Material S2—Figure S1: Methodological quality of the studies that evaluated the core musculature, Figure S2: Methodological quality of the studies that evaluated the lower limbs musculature, Figure S3: Methodological quality of the studies that evaluated the upper limbs musculature; Supplementary Online Material S3— Table S1: Evaluation of the quality of the selected studies with evaluations of internal validity and external validity of core muscles, Table S2: Evaluation of the quality of the selected studies with evaluations of internal validity and external validity of lower limbs muscles, Table S3: Evaluation of the quality of the selected studies with evaluations of internal validity and external validity of upper limbs muscles, Table S4: Evaluation of the quality of the selected studies with evaluations of internal validity and external validity of all muscles; Supplementary Online Material S4—Figure S1: Forest plot of the rectus abdominis muscle EMG activity on an unstable surface versus a stable surface, Figure S2: Forest plot of the external oblique muscle EMG activity on an unstable surface versus a stable surface, Figure S3: Forest plot of the internal oblique muscle EMG activity on an unstable surface versus a stable surface, Figure S4: Forest plot of the erector spinae muscle EMG activity on an unstable surface versus a stable surface, Figure S5: Forest plot of the lumbar multifidus muscle EMG activity on an unstable surface versus a stable surface, Figure S6: Forest plot of the pectoralis major muscle EMG activity on an unstable surface versus a stable surface, Figure S7: Forest plot of the triceps muscle EMG activity on an unstable surface versus a stable surface, Figure S8: Forest plot of the anterior deltoid muscle EMG activity on an unstable surface versus a stable surface, Figure S9: Forest plot of the rectus femoris muscle EMG activity on an unstable surface versus a stable surface, Figure S10: Forest plot of the vastus lateralis muscle EMG activity on an unstable surface versus a stable surface, Figure S11: Forest plot of the vastus medialis muscle EMG activity on an unstable surface versus a stable surface, Figure S12: Forest plot of the biceps femoris muscle EMG activity on an unstable surface versus a stable surface, Table S1: Intensity of exercises included in the meta-analysis; Supplementary Online Material S5: Sensitivity Analysis—Figure S1: Forest plot of the rectus abdominis muscle EMG activity on an unstable surface versus a stable surface, Figure S2: Forest plot of the external oblique muscle EMG activity on an unstable surface versus a stable surface, Figure S3: Forest plot of the erector spinae muscle EMG activity on an unstable surface versus a stable surface, Figure S4: Forest plot of the pectoralis major muscle EMG activity on an unstable surface versus a stable surface, Figure S5: Forest plot of the triceps muscle EMG activity on an unstable surface versus a stable surface, Figure S6: Forest plot of the anterior deltoid muscle EMG activity on an unstable surface versus a stable surface, Figure S7: Forest plot of the rectus femoris muscle EMG activity on an unstable surface versus a stable surface, Figure S8: Forest plot of the vastus lateralis muscle EMG activity on an unstable surface versus a stable surface, Figure S9: Forest plot of the vastus medialis muscle EMG activity on an unstable surface versus a stable surface, Figure S10: Forest plot of the biceps femoris muscle EMG activity on an unstable surface versus a stable surface.

## References

[1] Behm D.G., Anderson K.G. (2006). The role of instability with resistance training. J. Strength Cond. Res..

[2] Sousa D.S.F., de Farias W.M., de Amorim Batista G., de Oliveira V.M.A., Pirauá A.L.T., Beltrão N.B., Pitangui A.C.R., de Araújo R.C. (2022). Activation of Upper Limb Muscles in Subjects with Scapular Dyskinesis during Bench-Press and Dumbbell Fly on Stable and Unstable Surfaces. J. Back Musculoskelet. Rehabil..

[3] Palma F., Perramont N., Rojas V., Bertolotto B., Tuesta M. (2021). Electromyographic Amplitude and Coactivation of the Core Muscles during Different Unstable Push-up Exercises. Med. Sport.

[4] Bouillon L.E., Hofener M., O’Donnel A., Milligan A., Obrock C. (2019). Comparison of Muscle Activity Using Unstable Devices during a Forward Lunge. J. Sport. Rehabil..

[5] Buscà B., Aguilera-Castells J., Arboix-Alió J., Miró A., Fort-Vanmeerhaeghe A., Peña J. (2020). Influence of the Amount of Instability on the Leg Muscle Activity during a Loaded Free Barbell Half-Squat. Int. J. Environ. Res. Public Health.

[6] Kim S., Lee J.-H., Heo J., Chang E. (2021). Differences of Thigh Muscle Activation during Various Squat Exercise on Stable and Unstable Surfaces. Exerc. Sci..

[7] Pirauá A.L.T., Cavalcante B.R., Oliveira V.M.A., Beltrão N.B., Amorim Batista G., Pitangui A.C.R., Behm D., Araújo R.C. (2019). Effect of 24-week Strength Training on Unstable Surfaces on Mobility, Balance, and Concern about Falling in Older Adults. Scand. J. Med. Sci. Sports.

[8] Cavalcante B.R., de Souza M.F., Falck R.S., Liu-Ambrose T., Behm D.G., Pitangui A.C.R., de Araújo R.C. (2020). Effects of Resistance Exercise with Instability on Cognitive Function (REI Study): A Proof-Of-Concept Randomized Controlled Trial in Older Adults with Cognitive Complaints. J. Alzheimer’s Dis..

[9] Nascimento V.Y.S., Torres R.J.B., Beltrão N.B., dos Santos P.S., Pirauá A.L.T., de Oliveira V.M.A., Pitangui A.C.R., de Araújo R.C. (2017). Shoulder Muscle Activation Levels During Exercises with Axial and Rotational Load on Stable and Unstable Surfaces. J. Appl. Biomech..

[10] Melo B., Pirauá A., Beltrão N., Pitangui A.C., Araújo R. (2014). A Utilização de Superfície Instável Aumenta a Atividade Eletromiográfica Dos Músculos Da Cintura Escapular No Exercício Crucifixo. Rev. Bras. Atividade Física Saúde.

[11] Behm D.G., Muehlbauer T., Kibele A., Granacher U. (2015). Effects of Strength Training Using Unstable Surfaces on Strength, Power and Balance Performance Across the Lifespan: A Systematic Review and Meta-Analysis. Sports Med..

[12] Behm D., Colado J.C. (2012). The Effectiveness of Resistance Training Using Unstable Surfaces and Devices for Rehabilitation. Int. J. Sports Phys. Ther..

[13] Behm D.G., Colado Sanchez J.C. (2013). Instability Resistance Training across the Exercise Continuum. Sports Health.

[14] Luk J.T.C., Kwok F.K.C., Ho I.M.K., Wong D.P. (2021). Acute Responses of Core Muscle Activity during Bridge Exercises on the Floor vs. the Suspension System. Int. J. Environ. Res. Public Health.

[15] Kim S.J., Kwon O.Y., Yi C.H., Jeon H.S., Oh J.S., Cynn H.S., Weon J.H. (2011). Comparison of Abdominal Muscle Activity during a Single-Legged Hold in the Hook-Lying Position on the Floor and on a Round Foam Roll. J. Athl. Train..

[16] De Araújo R.C., De Andrade R., Tucci H.T., Martins J., De Oliveira A.S. (2011). Shoulder Muscular Activity during Isometric Three-Point Kneeling Exercise on Stable and Unstable Surfaces. J. Appl. Biomech..

[17] Snarr R.L., Esco M.R. (2013). Electromyographic Comparison of Traditional and Suspension Push-Ups. J. Hum. Kinet..

[18] Li Y., Cao C., Chen X. (2013). Similar Electromyographic Activities of Lower Limbs between Squatting on a Reebok Core Board and Ground. J. Strength Cond. Res..

[19] Youdas J.W., Hubble J.W., Johnson P.G., McCarthy M.M., Saenz M.M., Hollman J.H. (2020). Scapular Muscle Balance and Spinal Stabilizer Recruitment during an Inverted Row. Physiother. Theory Pract..

[20] Uribe B.P., Coburn J.W., Brown L.E., Judelson D.A., Khamoui A.V., Nguyen D. (2010). Muscle Activation When Performing the Chest Press and Shoulder Press on a Stable Bench vs. a Swiss Ball. J. Strength Cond. Res..

[21] De Araújo R.C., Andrade da Silva H., Pereira dos Passos M.H., Alves de Oliveira V.M., Rodarti Pitangui A.C. (2021). Use of Unstable Exercises in Periscapular Muscle Activity: A Systematic Review and Meta-Analysis of Electromyographic Studies. J. Bodyw. Mov. Ther..

[22] Mendez-Rebolledo G., Orozco-Chavez I., Morales-Verdugo J., Ramirez-Campillo R., Cools A.M.J. (2022). Electromyographic Analysis of the Serratus Anterior and Upper Trapezius in Closed Kinetic Chain Exercises Performed on Different Unstable Support Surfaces: A Systematic Review and Meta-Analysis. PeerJ.

[23] Page M.J., McKenzie J.E., Bossuyt P.M., Boutron I., Hoffmann T.C., Mulrow C.D., Shamseer L., Tetzlaff J.M., Akl E.A., Brennan S.E. (2021). The PRISMA 2020 Statement: An Updated Guideline for Reporting Systematic Reviews. BMJ.

[24] Syed-Abdul M.M., Soni D.S., Miller W.M., Johnson R.J., Barnes J.T., Pujol T.J., Wagganer J.D. (2018). Traditional vs. Suspended Pushup Muscle Activation in Athletes and Sedentary Females. J. Strength Cond. Res..

[25] Hwangbo G., Kim M.K. (2012). The Effects of Ankle Joint Position in Various Lower Limb Ground States on the Activation of the Shoulder and Trunk Muscles during Push-up Exercises. J. Phys. Ther. Sci..

[26] Calatayud J., Borreani S., Colado J.C., Martin F., Rogers M.E. (2014). Muscle Activity Levels in Upper-Body Push Exercises with Different Loads and Stability Conditions. Physician Sportsmed..

[27] Borreani S., Calatayud J., Colado J.C., Tella V., Moya-Nájera D., Martin F., Rogers M.E. (2015). Shoulder Muscle Activation during Stable and Suspended Push-Ups at Different Heights in Healthy Subjects. Phys. Ther. Sport.

[28] Calatayud J., Borreani S., Colado J.C., Martín F.F., Rogers M.E., Behm D.G., Andersen L.L. (2014). Muscle Activation during Push-Ups with Different Suspension Training Systems. J. Sports Sci. Med..

[29] Borreani S., Calatayud J., Colado J.C., Moya-Nájera D., Triplett N.T., Martin F. (2015). Muscle Activation during Push-Ups Performed under Stable and Unstable Conditions. J. Exerc. Sci. Fit..

[30] Kim M.-K., Jung J.-M., Lee S.-Y., Hwangbo G., Lee Y.-S. (2012). Effects of Various Lower Limb Ground States on Activation of the Shoulder and Trunk Muscles during Push-up Exercises. J. Phys. Ther. Sci..

[31] Youdas J.W., Hartman J.P., Murphy B.A., Rundle A.M., Ugorowski J.M., Hollman J.H. (2015). Magnitudes of Muscle Activation of Spine Stabilizers, Gluteals, and Hamstrings during Supine Bridge to Neutral Position. Physiother. Theory Pract..

[32] Sundstrup E., Jakobsen M.D., Andersen C.H., Jay K., Andersen L.L. (2012). Swiss Ball Abdominal Crunch with Added Elastic Resistance Is an Effective Alternative to Training Machines. Int. J. Sports Phys. Ther..

[33] Aguilera-Castells J., Buscà B., Morales J., Solana-Tramunt M., Fort-Vanmeerhaeghe A., Rey-Abella F., Bantulà J., Peña J. (2019). Muscle Activity of Bulgarian Squat. Effects of Additional Vibration, Suspension and Unstable Surface. PLoS ONE.

[34] Siegfried N., Muller M., Deeks J., Volmink J., Egger M., Low N., Walker S., Williamson P. (2005). HIV and Male Circumcision—A Systematic Review with Assessment of the Quality of Studies. Lancet Infect. Dis..

[35] Kinsella R., Pizzari T. (2017). Electromyographic Activity of the Shoulder Muscles during Rehabilitation Exercises in Subjects with and without Subacromial Pain Syndrome: A Systematic Review. Shoulder Elbow.

[36] Higgins J.P.T., Thomas J., Chandler J., Cumpston M., Li T., Page M.J., Welch V.A. (2019). Cochrane Handbook for Systematic Reviews of Interventions.

[37] Oliva-Lozano J.M., Muyor J.M. (2020). Core Muscle Activity during Physical Fitness Exercises: A Systematic Review. Int. J. Environ. Res. Public Health.

[38] Marshall P., Murphy B. (2006). Changes in Muscle Activity and Perceived Exertion during Exercises Performed on a Swiss Ball. Appl. Physiol. Nutr. Metab..

[39] Aranda L.C., Mancini M., Werneck F.Z., Novaes J.D.S., Da Silva-Grigoletto M.E., Vianna J.M. (2016). Electromyographic Activity and 15RM Load during Resistance Exercises on Stable and Unstable Surfaces. J. Exerc. Physiol. Online.

[40] Behm D.G., Leonard A.M., Young W.B., Bonsey W.A.C., MacKinnon S.N. (2005). Trunk Muscle Electromyographic Activity with Unstable and Unilateral Exercises. J. Strength Cond. Res..

[41] Snarr R.L., Esco M.R. (2014). Electromyographical Comparison of Plank Variations Performed with and without Instability Devices. J. Strength. Cond. Res..

[42] Atkins S.J., Bentley I., Brooks D., Burrows M.P., Hurst H.T., Sinclair J.K. (2015). Electromyographic Response of Global Abdominal Stabilizers in Response to Stable- and Unstable-Base Isometric Exercise. J. Strength Cond. Res..

[43] Lee S., Park J., Lee D. (2015). Effects of Bridge Exercise Performed on an Unstable Surface on Lumbar Stabilizing Muscles According to the Knee Angle. J. Phys. Ther. Sci..

[44] Byrne J.M., Bishop N.S., Caines A.M., Crane K.A., Feaver A.M., Pearcey G.E.P. (2014). Effect of Using a Suspension Training System on Muscle Activation during the Performance of a Front Plank Exercise. J. Strength Cond. Res..

[45] Czaprowski D., Afeltowicz A., Gebicka A., Pawłowska P., Kedra A., Barrios C., Hadała M. (2014). Abdominal Muscle EMG-Activity during Bridge Exercises on Stable and Unstable Surfaces. Phys. Ther. Sport.

[46] Biscarini A., Contemori S., Grolla G. (2019). Activation of Scapular and Lumbopelvic Muscles During Core Exercises Executed on a Whole-Body Wobble Board. J. Sport Rehabil..

[47] Imai A., Kaneoka K., Okubo Y., Shiina I., Tatsumura M., Izumi S., Shiraki H. (2010). Trunk Muscle Activity during Lumbar Stabilization Exercises on Both a Stable and Unstable Surface. J. Orthop. Sports Phys. Ther..

[48] Yoon J.-O., Kang M.-H., Kim J.-S., Oh J.-S. (2018). Effect of Modified Bridge Exercise on Trunk Muscle Activity in Healthy Adults: A Cross Sectional Study. Braz. J. Phys. Ther..

[49] Andrade L.S., Mochizuki L., Pires F.O., da Silva R.A.S., Mota Y.L. (2015). Application of Pilates Principles Increases Paraspinal Muscle Activation. J. Bodyw. Mov. Ther..

[50] Youdas J.W., Coleman K.C., Holstad E.E., Long S.D., Veldkamp N.L., Hollman J.H. (2017). Magnitudes of Muscle Activation of Spine Stabilizers in Healthy Adults during Prone on Elbow Planking Exercises with and without a Fitness Ball. Physiother. Theory Pract..

[51] Vilaça-Alves J., Guimarães F., Rosa C., Neves E.B., Saavedra F., Fernandes A.O., Reis V.M. (2016). Electromyography Analysis of the Abdominal Crunch in Stable and Unstable Surface. Gazz. Medica Ital. Arch. Sci. Mediche.

[52] Sternlicht E., Rugg S., Fujii L.L., Tomomitsu K.F., Seki M.M. (2007). Electromyographic Comparison of a Stability Ball Crunch with a Traditional Crunch. J. Strength. Cond. Res..

[53] Feldwieser F.M., Sheeran L., Meana-Esteban A., Sparkes V. (2012). Electromyographic Analysis of Trunk-Muscle Activity during Stable, Unstable and Unilateral Bridging Exercises in Healthy Individuals. Eur. Spine J..

[54] Kim M.-H., Oh J.-S. (2015). Effects of Performing an Abdominal Hollowing Exercise on Trunk Muscle Activity during Curl-up Exercise on an Unstable Surface. J. Phys. Ther. Sci..

[55] Saeterbakken A.H., Andersen V., Jansson J., Kvellestad A.C., Fimland M.S. (2014). Effects of BOSU Ball(s) During Sit-Ups with Body Weight and Added Resistance on Core Muscle Activation. J. Strength Cond. Res..

[56] Lehman G.J., Gordon T., Langley J., Pemrose P., Tregaskis S. (2005). Replacing a Swiss Ball for an Exercise Bench Causes Variable Changes in Trunk Muscle Activity during Upper Limb Strength Exercises. Dyn. Med..

[57] Ha S., Oh J., Jeon I., Kwon O. (2015). The Effects of Surface Condition on Abdominal Muscle Activity during Single-Legged Hold Exercise. J. Electromyogr. Kinesiol..

[58] Lee D., Lee Y., Cho H.-Y., Lee K.-B., Hong S., Pyo S., Lee G. (2017). Investigation of Trunk Muscle Activity for Modified Plank Exercise: A Preliminary Study. Isokinet. Exerc. Sci..

[59] Kim J.H., Kim Y., Chung Y. (2014). The Influence of an Unstable Surface on Trunk and Lower Extremity Muscle Activities during Variable Bridging Exercises. J. Phys. Ther. Sci..

[60] Kim C.-M., Kong Y.-S., Hwang Y.-T., Park J. (2018). The Effect of the Trunk and Gluteus Maximus Muscle Activities According to Support Surface and Hip Joint Rotation during Bridge Exercise. J. Phys. Ther. Sci..

[61] Hyong I.H., Kang J.H. (2013). Activities of the Vastus Lateralis and Vastus Medialis Oblique Muscles during Squats on Different Surfaces. J. Phys. Ther. Sci..

[62] Park J.-K., Lee D.-Y., Kim J.-S., Hong J.-H., You J.-H., Park I.-M. (2015). Effects of Visibility and Types of the Ground Surface on the Muscle Activities of the Vastus Medialis Oblique and Vastus Lateralis. J. Phys. Ther. Sci..

[63] Souto Maior B., Simão A., Freitas De Salles R., Miranda B., Costa H., Brando P. (2009). Neuromuscular activity during the squat exercise on an unstable platform. Braz. J. Biomot..

[64] McBride J.M., Larkin T.R., Dayne A.M., Haines T.L., Kirby T.J. (2010). Effect of Absolute and Relative Loading on Muscle Activity during Stable and Unstable Squatting. Int. J. Sports Physiol. Perform..

[65] McBride J.M., Cormie P., Deane R. (2006). Isometric Squat Force Output and Muscle Activity in Stable and Unstable Conditions. J. Strength Cond. Res..

[66] Andersen V., Fimland M.S., Brennset O., Haslestad L.R., Lundteigen M.S., Skalleberg K., Saeterbakken A.H. (2014). Muscle Activation and Strength in Squat and Bulgarian Squat on Stable and Unstable Surface. Int. J. Sports Med..

[67] Anderson K., Behm D.G. (2005). Trunk Muscle Activity Increases with Unstable Squat Movements. Can. J. Appl. Physiol..

[68] Jeon G.-R., Yu Y.-W., To M., Hong J.-H., Yu J.-H., Kim J.-S., Lee D.-Y. (2020). A Study on the Selective Strengthening Exercise of the Quadriceps Muscle According to Various Squat Types. Med.-Leg. Update.

[69] Kang J.-I., Park J.-S., Choi H., Jeong D.-K., Kwon H.-M., Moon Y.-J. (2017). A Study on Muscle Activity and Ratio of the Knee Extensor Depending on the Types of Squat Exercise. J. Phys. Ther. Sci..

[70] Lee S., Choi Y.H., Kim J. (2017). The Effects of Changes in Support and Inclined Boards on Lower-Extremity Muscle Activity. J. Phys. Ther. Sci..

[71] Marín P.J., Hazell T.J. (2014). Effects of Whole-Body Vibration with an Unstable Surface on Muscle Activation. J. Musculoskelet. Neuronal Interact..

[72] Saeterbakken A.H., Fimland M.S. (2013). Muscle Force Output and Electromyographic Activity in Squats with Various Unstable Surfaces. J. Strength Cond. Res..

[73] Gündoğan B., Aydin E.M., Sağlam A.F. (2023). Muscle Activation during Squat on Different Surfaces. Pamukkale J. Sport. Sci..

[74] Nairn B.C., Sutherland C.A., Drake J.D.M. (2017). Motion and Muscle Activity Are Affected by Instability Location during a Squat Exercise. J. Strength Cond. Res..

[75] Han D., Nam S., Song J., Lee W., Kang T. (2017). The Effect of Knee Flexion Angles and Ground Conditions on the Muscle Activation of the Lower Extremity in the Squat Position. J. Phys. Ther. Sci..

[76] Krause D.A., Elliott J.J., Fraboni D.F., McWilliams T.J., Rebhan R.L., Hollman J.H. (2018). Electromyography of the hip and thigh muscles during two variations of the lunge exercise: A cross-sectional study. Int. J. Sports Phys. Ther..

[77] Miller W.M., Barnes J.T., Sofo S.S., Wagganer J.D. (2019). Comparison of Myoelectric Activity During a Suspension-Based and Traditional Split Squat. J. Strength Cond. Res..

[78] Wahl M.J., Behm D.G. (2008). Not All Instability Training Devices Enhance Muscle Activation in Highly Resistance-Trained Individuals. J. Strength Cond. Res..

[79] Behm D.G., Anderson K., Curnew R.S. (2002). Muscle Force and Activation under Stable and Unstable Conditions. J. Strength Cond. Res..

[80] Kim J.-W., Han J.-Y., Kang M.-H., Ha S.-M., Oh J.-S. (2013). Comparison of Posterior Oblique Sling Activity during Hip Extension in the Prone Position on the Floor and on a Round Foam Roll. J. Phys. Ther. Sci..

[81] Sandhu J., Mahajan S., Shenoy S. (2008). An Electromyographic Analysis of Shoulder Muscle Activation during Push-up Variations on Stable and Labile Surfaces. Int. J. Shoulder Surg..

[82] De Mey K., Danneels L., Cagnie B., Borms D., T’Jonck Z., Van Damme E., Cools A.M. (2014). Shoulder Muscle Activation Levels during Four Closed Kinetic Chain Exercises with and without Redcord Slings. J. Strength Cond. Res..

[83] Park S.-Y., Yoo W.-G. (2013). Effects of Push-up Exercise Phase and Surface Stability on Activation of the Scapulothoracic Musculature. Int. J. Athl. Ther. Train..

[84] Nairn B.C., Sutherland C.A., Drake J.D.M. (2015). Location of Instability During a Bench Press Alters Movement Patterns and Electromyographical Activity. J. Strength Cond. Res..

[85] Lehman G.J., MacMillan B., MacIntyre I., Chivers M., Fluter M. (2006). Shoulder Muscle EMG Activity during Push up Variations on and off a Swiss Ball. Dyn. Med..

[86] Torres R.J.B., Pirauá A.L.T., Nascimento V.Y.S., dos Santos P.S., Beltrão N.B., de Oliveira V.M.A., Pitangui A.C.R., de Araújo R.C. (2017). Shoulder Muscle Activation Levels during the Push-up-plus Exercise on Stable and Unstable Surfaces. J. Sport. Rehabil..

[87] De Bezerra E.S., da Orssatto L.B.R., Werlang L.C., Generoso A.M., Moraes G., Sakugawa R.L. (2020). Effect of Push-up Variations Performed with Swiss Ball on Muscle Electromyographic Amplitude in Trained Men: A Cross-Sectional Study. J. Bodyw. Mov. Ther..

[88] de Oliveira A.S., de Morais Carvalho M., de Brum D.P.C. (2008). Activation of the Shoulder and Arm Muscles during Axial Load Exercises on a Stable Base of Support and on a Medicine Ball. J. Electromyogr. Kinesiol..

[89] Herrington L., Waterman R., Smith L. (2015). Electromyographic Analysis of Shoulder Muscles during Press-up Variations and Progressions. J. Electromyogr. Kinesiol..

[90] Pontillo M., Orishimo K.F., Kremenic I.J., McHugh M.P., Mullaney M.J., Tyler T.F. (2007). Shoulder Musculature Activity and Stabilization during Upper Extremity Weight-Bearing Activities. N. Am. J. Sports Phys. Ther..

[91] Patterson J.M., Vigotsky A.D., Oppenheimer N.E., Feser E.H. (2015). Differences in Unilateral Chest Press Muscle Activation and Kinematics on a Stable versus Unstable Surface While Holding One versus Two Dumbbells. PeerJ.

[92] Anderson K.G., Behm D.G. (2004). Maintenance of EMG Activity and Loss of Force Output with Instability. J. Strength Cond. Res..

[93] Marshall P.W.M., Murphy B.A. (2006). Increased Deltoid and Abdominal Muscle Activity during Swiss Ball Bench Press. J. Strength Cond. Res..

[94] Saeterbakken A.H., Fimland M.S. (2013). Electromyographic Activity and 6RM Strength in Bench Press on Stable and Unstable Surfaces. J. Strength Cond. Res..

[95] Reiser F.C., Lira J.L.O., Bonfim B.M.A., Filho S.J.A.S., Durante B.G., Cardoso J.M.D., Miotto H., Soares M.A.A., Bonuzzi G.M.G., Tavares L.D. (2017). Electromyography of Dumbbell Fly Exercise Using Different Planes and Labile Surfaces. J. Exerc. Physiol. Online.

[96] Park S., Yoo W., Kwon H., Kim D., Lee S., Park M. (2013). Scapulothoracic Muscle Activation on Stable and Unstable Support Surfaces. Int. J. Athl. Ther. Train..

[97] Anderson G.S., Gaetz M., Holzmann M., Twist P. (2013). Comparison of EMG Activity during Stable and Unstable Push-up Protocols. Eur. J. Sport. Sci..

[98] Kohler J.M., Flanagan S.P., Whiting W.C. (2010). Muscle Activation Patterns While Lifting Stable and Unstable Loads on Stable and Unstable Surfaces. J. Strength Cond. Res..

[99] Jeong S.Y., Chung S.H., Shim J.H. (2014). Comparison of Upper Trapezius, Anterior Deltoid, and Serratus Anterior Muscle Activity during Pushup plus Exercise on Slings and a Stable Surface. J. Phys. Ther. Sci..

[100] Kendall F., McCreary E., Provance P. (1993). Muscles: Testing and Function.

[101] George S.Z., Fritz J.M., Silfies S.P., Schneider M.J., Beneciuk J.M., Lentz T.A., Gilliam J.R., Hendren S., Norman K.S., Beattie P.F. (2021). Interventions for the Management of Acute and Chronic Low Back Pain: Revision 2021. J. Orthop. Sports Phys. Ther..

[102] Seo B.-D., Yun Y.-D., Kim H.-R., Lee S.-H. (2012). Effect of 12-Week Swiss Ball Exercise Program on Physical Fitness and Balance Ability of Elderly Women. J. Phys. Ther. Sci..

[103] Pirauá A.L.T., Pitangui A.C.R., Silva J.P., dos Passos M.H.P., de Oliveira V.M.A., da Batista L.S.P., de Araújo R.C. (2014). Electromyographic Analysis of the Serratus Anterior and Trapezius Muscles during Push-Ups on Stable and Unstable Bases in Subjects with Scapular Dyskinesis. J. Electromyogr. Kinesiol..

[104] Ebben W.P., Wurm B., VanderZanden T.L., Spadavecchia M.L., Durocher J.J., Bickham C.T., Petushek E.J. (2011). Kinetic Analysis of Several Variations of Push-Ups. J. Strength Cond. Res..

[105] Pirauá A.L.T., Beltrão N.B., Santos C.X., Pitangui A.C.R., de Araújo R.C. (2017). Analysis of Muscle Activity during the Bench Press Exercise Performed with the Pre-Activation Method on Stable and Unstable Surfaces. Kinesiology.

[106] Lephart S.M., Henry T.J. (1996). The Physiological Basis for Open and Closed Kinetic Chain Rehabilitation for the Upper Extremity. J. Sports Rehab..

