# Supplementary material for: Comparison of the Electromyography Activity during Exercises with Stable and Unstable Surfaces: A Systematic Review and Meta-Analysis
Source: Sports (Basel). 2024 Apr 18;12(4):111. doi: 10.3390/sports12040111 (PMC11055131; doi:10.3390/sports12040111)
Supplement: Supplementary file 1 [file sports-12-00111-s001.zip › Supplementary Online Material S1.pdf]

Title: Comparison of the electromyography activity during exercises with stable and unstable surfaces: A systematic review and meta-analysis

Supplementary Online Material S1

1. **Electronic searches:** A systematic review of the literature was conducted on six electronic databases for studies published until March 2023 (Pubmed, Web of Science, Scopus, Cochrane Library, Scielo and Lilacs).
2. **PICOS Strategy:** 1) Population: athletes and non-athletic adults from both sexes who were experienced with exercises using instability devices; (2) Interventions: exercises for the upper, lower limbs or trunk/core using an unstable surface; (3) Comparators: the same exercises performed on a stable base; (4) Outcomes: EMG amplitude values of the agonist muscles; (5) Study type: cross-sectional studies that compared electromyographic activity during an exercise with and without unstable surfaces.

Data base	Expressions	Results
**PUBMED**	(((((((((("instability resistance training") OR ("instability strength training")) OR ("free-weight training")) OR ("instability weight-bearing exercise program")) OR ("instability weight-bearing strengthening program")) OR ("instability weight-lifting exercise program")) OR ("weight-lifting strengthening program")) OR (unstable)) OR (instability)) OR ("unstable training")) OR ("unstable surface")) AND (((((((Electromyography) OR (Electromyographies)) OR (Electromyographic)) OR ("Surface Electromyography")) OR (EMG)) OR ("Muscle activity")) OR ("Muscle activation"))	3676
**Cochrane Library**	Electromyography OR Electromyographies OR Electromyographic OR "Surface Electromyography" OR EMG OR "Muscle activity" OR "Muscle activation" in Title Abstract Keyword AND "instability resistance training" OR "instability strength training" OR "free-weight training" OR "instability weight-bearing exercise program" OR "instability weight-bearing strengthening program" OR "instability weight-lifting exercise program" OR "weight-lifting strengthening program" OR unstable OR instability OR "unstable training" OR "unstable surface" in Title Abstract Keyword - (Word variations have been searched)	346
**Scielo**	("instability resistance training") OR ("instability strength training") OR ("free-weight training") OR ("instability weight-bearing exercise program") OR ("instability weight-bearing strengthening program") OR ("instability weight-lifting exercise program") OR ("weight-lifting strengthening program") OR (unstable) OR (instability) OR ("unstable training") OR ("unstable surface") AND (Electromyography) OR (Electromyographies) OR (Electromyographic) OR ("Surface Electromyography") OR (EMG) OR ("Muscle activity") OR ("Muscle activation")	7
**Scopus**	TITLE-ABS-KEY (electromyography) OR TITLE-ABS-KEY (electromyographies) OR TITLE-ABS-KEY (electromyographic) OR TITLE-ABS-KEY ("Surface Electromyography") OR TITLE-ABS-KEY (EMG) OR TITLE-ABS-KEY ("Muscle activity") OR TITLE-ABS-KEY ("Muscle activation") AND TITLE-ABS-KEY (unstable) OR TITLE-ABS-KEY (instability) OR TITLE-ABS-KEY ("instability resistance training") OR TITLE-ABS-KEY ("instability strength training") OR TITLE-ABS-KEY ("free-weight training") OR TITLE-ABS-KEY ("instability weight-bearing exercise program") OR TITLE-ABS-KEY ("instability weight-bearing strengthening program") OR TITLE-ABS-KEY ("instability weight-lifting exercise program") OR TITLE-ABS-KEY ("weight-lifting strengthening program") OR TITLE-ABS-KEY ("unstable training") OR TITLE-ABS-KEY ("unstable surface")	2985
**Web of Science**	(ALL=("instability resistance training" OR "instability strength training" OR "free-weight training" OR "instability weight-bearing exercise program" OR "instability weight-bearing strengthening program" OR "instability weight-lifting exercise program" OR "weight-lifting strengthening program" OR unstable OR instability OR "unstable training" OR "unstable surface")) AND ALL=(Electromyography OR electromyographics OR Electromyographic OR "Surface Electromyography" OR EMG OR "Muscle activity" OR "Muscle activation")	2493
**LILACS**	(tw:("instability resistance training")) OR (tw:("instability strength training")) OR (tw:("free-weight training")) OR (tw:("instability weight-bearing exercise program")) OR (tw:("instability weight-bearing strengthening program")) OR (tw:("instability weight-lifting exercise program")) OR (tw:("weight-lifting strengthening program")) OR (tw:(unstable)) OR (tw:(instability)) OR (tw:("unstable training")) OR (tw:("unstable surface")) AND (tw:(Electromyography)) OR (tw:(Electromyographies)) OR (tw:(Electromyographic)) OR (tw:("Surface Electromyography")) OR (tw:(EMG)) OR (tw:("Muscle activity")) OR (tw:("Muscle activation"))	19
**Total articles**		**9526**

Table 1. Summary of the characteristics and results of the selected studies to core muscles.

Authors	Sample	Muscles	Exercise	Unstable surface	Stable surface	Main Results
(BEHM et al., 2005)	n = 6 males; 5 females 24.1 ± 7.4 years old	Upper lumbar, lumbosacral erector spinae, lower-abdominal.	Bridge, pelvic tilt, alternate arm and leg extension, parallel hold, side bridge, superman.	Swiss ball.	Bench.	↑ Unstable surface (lower-abdominal) ↔ EMG activity (upper lumbar, lumbosacral erector spinae)
(MARSHALL; MURPHY, 2006a)	n = 8 males; 4 females 22.1 ± 2.4 years old	Rectus abdominis, external oblique.	Double leg hold.	Swiss ball.	Stable surface.	↑ Unstable surface (rectus abdominis) ↔ EMG activity (external oblique)
(YOUDAS et al., 2015)	n = 13 males; 13 females 23.4 ± 1.2 years old	Lumbar multifidus	Double-leg bridge, single-leg bridge, Double-leg bridge with hamstring curl.	Swiss ball, Bosu ball.	Floor, slider sheet.	↔ EMG activity (lumbar multifidus)
(KIM; KIM; CHUNG, 2014)	n = 30 females 37.13 ± 6.39 years old	External oblique, erector spinae.	Double-leg bridge, Single-leg bridge, Single-leg cross bridging.	Air cushion.	Floor.	↑ Unstable surface (external oblique erector spinae)
(YOON et al., 2018)	n = 12 males; 3 females 27.47 ± 3.04 years old	Rectus abdominis, internal oblique, erector spinae, multifidus.	Single-leg bridge.	Dynamic air cushion.	Stable surface.	↔ EMG activity (rectus abdominis, internal oblique, erector spinae, multifidus)
(ANDRADE et al., 2015)	n = 19 females 25.6 ± 5 years old	Rectus abdominis, lumbar multifidus.	Crunch exercise.	Foam roller.	Floor.	↔ EMG activity (rectus abdominis) ↑ Stable surface (lumbar multifidus)
(YOUDAS et al., 2017)	n = 13 males; 13 females 25.2 ± 4.7 years old	Lumbar multifidi, rectus abdominis, external oblique, internal oblique.	Prone plank.	Swiss ball.	Floor.	↔ EMG activity (lumbar multifidi, internal oblique, rectus abdominis, external oblique)
(VILAÇA-ALVES et al., 2016)	n = 20 males 22.4 ± 2.4 years old	Rectus abdominal, external oblique.	Crunch exercise.	Swiss ball.	Floor.	↔ EMG activity (rectus abdominal, external oblique)
(STERNLICHT et al., 2007)	n = 23 males; 18 females 20.3 ± 1.5 years old	Upper Rectus abdominis, lower rectus abdominis, external oblique.	Abdominal crunch.	Swiss ball.	Floor.	↓ Unstable surface when supporting the Swiss ball in the upper region (upper rectus abdominis, lower rectus abdominis, external oblique). However, there was no difference between the surfaces when the ball was placed in the inferior region.
(LUK et al., 2021)	N = 43 females 21.40 ± 1.78 years old	Rectus abdominis, lumbar multifidus, erector spinae.	Double-leg bridge, Prone plank.	TRX.	Floor.	↑ in activity of the rectus abdominal, erector spinae, lumbar multifidus on unstable surface (**Suspension on feet**) in comparison with Supine-arm suspension and stable surface.

Table 1. *(continued)*

(FELDWIESER et al., 2012)	n = 4 males; 16 females<br>25.45 ±3.57 years old	Lumbar multifidus, rectus abdominis, external oblique.	Double-leg bridge, single-leg bridge.	Ball cushion, Swiss ball.	Floor.	**Double-leg bridge**<br>↔ EMG activity (rectus abdominis, external oblique, lumbar multifidus)<br><br>**Single-leg bridge**<br>↔ EMG activity (lumbar multifidus, external oblique, rectus abdominis)
(KIM; OH, 2015)	n = 9 males; 5 females<br>20.6 ± 1.6 years old	Rectus abdominis, external oblique, internal oblique, transverse abdominis.	Curl-up.	Swiss ball.	Floor.	↔ EMG activity (rectus abdominis, external oblique, internal oblique)<br>↑ Unstable surface (transverse abdominis)
(SAETERBAKKEN et al., 2014)	n = 24 males<br>23 ± 2 years old	Rectus abdominis, external oblique.	Sit-ups with body weight and elastic resistance.	Bosu ball.	Stable surface.	**Sit-ups with body weight**<br>↔ EMG activity (rectus abdominis)<br>↑ Stable surface (external oblique)<br><br>**Sit-ups with elastic resistance**<br>↑ Unstable surface (rectus abdominis)<br>↔ EMG activity (external oblique)
(SUNDSTRUP et al., 2012)	n = 18 males; 24 females<br>42 ± 11 years old	Rectus abdominis, external oblique, erector spinae.	Abdominal crunch.	Swiss ball.	Machine.	↑ Unstable surface (rectus abdominis)<br>↔ EMG activity (external oblique, erector spinae)
(LEHMAN et al., 2005)	n = 7 males; 5 females<br>25.9 ± 5.5 years old	Rectus abdominis, external oblique, internal oblique, erector spinae.	Curl up.	Swiss ball.	Flat bench.	↔ EMG activity (rectus abdominis, external oblique, internal oblique, erector spinae)
(HA et al., 2015)	n = 13 males<br>39.0 ± 6.5 years old	Rectus abdominis, external oblique, internal oblique.	Single-leg hold.	Foam-roll, motorized rotating platform.	Floor.	↑ Unstable surface (internal oblique) for foam-roll,<br>↔ EMG activity (rectus abdominis, external oblique)
(KIM et al., 2018)	n = 28 males<br>26.43 ± 3.42 years old	External oblique, erector spinae.	Bridge.	Swiss ball.	Stable surface.	↑ Unstable surface (external oblique)<br>↔ EMG activity (erector spinae)
(ATKINS et al., 2015)	n = 18 males<br>15.9 ± 2 years old	Rectus abdominis, external oblique, erector spinae.	Prone plank.	Swiss ball, TRX.	Floor.	↑ Unstable surface—TRX (rectus abdominis)<br>↑ Stable surface (external oblique)<br>↔ EMG activity (erector spinae)
(LEE; PARK; LEE, 2015)	n = 15 males<br>22.2 ± 2.0 years old	External oblique, internal oblique, rectus abdominis.	Double-leg bridge.	Aero-step (Aero-step XL, TOGU, Germany).	Floor.	↑ Unstable surface (external oblique, internal oblique, rectus abdominis) with angulation of 120°
(BYRNE et al., 2014)	n = 11 males; 10 females<br>21.9 ± 2.4 years old	Rectus abdominis, external oblique.	Prone plank.	TRX.	Floor.	↑ Unstable surface (rectus abdominis, external oblique)
(KIM et al., 2011)	n =11 males; 8 females<br>23.2 ± 2.3 years old	Rectus abdominis, external oblique.	Single-legged hold.	Round foam roll.	Floor.	↑ Unstable surface (all muscles)

Table 1. ***(continued)***

(IMAI et al., 2010)	n = 9 males<br>24.1 ± 0.8 years old	External oblique, erector spinae, lumbar multifidus, rectus abdominis, transverse abdominis.	Elbow–toe, back bridge, Hand–knee, side bridge, Curl-up.	Bosu ball, Swiss ball, Balance disk.	Floor.	**Elbow-toe**<br>↑ Unstable surface (all muscles)<br><br>**Back bridge**<br>↔ EMG activity (all muscles)<br><br>**Hand-knee**<br>↑ Unstable surface (rectus abdominis, external oblique, erector spinae).<br>↔ EMG activity (transverse abdominis, lumbar multifidus)<br><br>**Side bridge**<br>↑ Unstable surface (rectus abdominis)<br>↔ EMG activity (external oblique, transverse abdominis, erector spinae, lumbar multifidus)<br><br>**Curl-up**<br>↑ Unstable surface (external oblique)<br>↑ Stable surface (transverse abdominis)<br>↔ EMG activity (rectus abdominis, erector spinae, lumbar multifidus)
(SNARR; ESCO, 2014)	n= 6 males; 6 females<br>23.25 ± 2.95 years old	Rectus abdominis, external oblique, lumbosacral erector spinae.	Prone plank.	Swiss ball, TRX.	Floor.	↑ Unstable surface (all muscles), but in different exercises<br>↑ EMG activity was verified for the **elbows in suspension device plank** (rectus abdominis, external oblique and lumbosacral erector spinae), but no difference was found for the **feet on ball plank** (external oblique and lumbosacral erector spinae) and **elbows on ball plank** (lumbosacral erector spinae)
(CZAPROWSKI et al., 2014)	n = 15 males; 18 females<br>23.2 ± 2.5 years old	Rectus abdominis, external oblique.	Prone plank,<br><br>side bridge,<br><br>double-leg bridge.	Bosu ball, Swiss ball.	Stable surface (Thera-Band, Canada).	**Prone plank**<br>↑ Unstable surface (Swiss ball) of the rectus abdominis, external oblique<br><br>**Side bridge**<br>↑ Unstable surface (Swiss ball) of the rectus abdominis, external oblique<br><br>**Double-leg bridge**<br>↑ Unstable surface (Swiss ball) of the rectus abdominis, external oblique

Table 1. ***(continued)***

(BISCARINI; CONTEMORI; GROLLA, 2019)	n = 11 males; 7 females<br>29 years old<br>Range 21-52 years old	Rectus abdominis, external oblique, internal oblique, erector spinae, multifidus.	double-leg bridge,<br>side Bridge,<br>prone Plank,<br>supine position with hip flexed at 90◦,<br>bird dog.	Wobble board.	Floor.	**Double-leg bridge**<br>↔ EMG activity (erector spinae, multifidus)<br>↑ Unstable surface (rectus abdominis, internal oblique, external oblique)<br>**Side Bridge**<br>↑ Unstable surface (rectus abdominis, external oblique, internal oblique)<br>↔ EMG activity (erector spinae, multifidus)<br>**Prone Plank**<br>↔ EMG activity (rectus abdominis, multifidus, erector spinae, internal oblique)<br>↑ Unstable surface (external oblique)<br>**Supine position with hip flexed at 90◦**<br>↔ EMG activity (rectus abdominis, external oblique, internal oblique)<br>Does not display the data (erector spinae, multifidus)<br>**Bird dog**<br>↑ Unstable surface (rectus abdominis, external oblique, internal oblique)<br>↑ Stable surface (erector spinae, multifidus)
(LEE et al., 2017)	n = 6 males; 1 female<br>22,6 ± 2.23 years old	External oblique, rectus abdominis, erector spinae.	Prone plank,<br>prone plank on knees.	Dynamic cushion.	Stable surface.	**Prone plank**<br>↔ EMG activity (rectus abdominis, external oblique, erector spinae)<br>**Prone plank on knees**<br>↔ EMG activity (rectus abdominis, external oblique, erector spinae)

Table 2. Summary of the characteristics and results of the selected studies to lower limbs muscles.

Authors	Sample	Muscles	Exercise	Unstable surface	Stable surface	Main Results
(ANDERSON; BEHM, 2005)	n = 14 males<br>25.2 ± 6.2 years old	Vastus lateralis,<br>biceps femoris.	Squat,<br><br>Smith squat.	Balance discs.	Floor.	**Squat free**<br>↔ EMG activity (vastus lateralis, biceps femoris)<br><br>**Smith squat**<br>↔ EMG activity (vastus lateralis, biceps femoris)
(MCBRIDE et al., 2010)	n = 10 males<br>24.1 ± 2.0 years old	Vastus lateralis,<br>biceps femoris.	Squat.	Does not specify.	Floor.	↑ Stable surface, except during the concentric phase of the absolute loading condition of 75 kg (vastus lateralis).<br><br>↑ Stable surface, during four of the twelve conditions (biceps femoris)
(LI; CAO; CHEN, 2013)	n = 13 males<br>19.4 ± 1.2 years old	Vastus lateralis,<br>vastus medialis,<br>rectus femoris,<br>biceps femoris,<br>gluteus maximus.	Squat.	Reebok Core Board.	Floor.	↔ EMG activity (all muscles)
(ANDERSEN et al., 2014)	n = 15 males<br>24 ± 4 years old	Vastus lateralis,<br>vastus medialis,<br>rectus femoris,<br>biceps femoris.	Squat,<br><br>Bulgarian squat.	Foam cushion.	Floor.	**Squat**<br>↔ EMG activity (all muscles)<br><br>**Bulgarian squat**<br>↑ Stable surface (biceps femoris)<br>↔ EMG activity (rectus femoris, vastus lateralis, vastus medialis)
(NAIRN; SUTHERLAND; DRAKE, 2017)	n = 10 males<br>21 ± 3 years old	Gluteus maximus,<br>rectus femoris,<br>biceps femoris,<br>vastus medialis.	Squat.	Bosu ball,<br>Tube.	Floor.	↔ EMG activity (gluteus maximus)<br><br>↑ Unstable surface (rectus femoris, vastus medialis, biceps femoris)
(HAN et al., 2017)	n = 15 females<br>21.5 ± 0.7 years old	Vastus medialis,<br>biceps femoris.	Half squat (70°),<br>Half squat (90°),<br>Deep squat (100°).	Aero step.	Floor.	↔ EMG activity (all muscles)
(WAHL; BEHM, 2008)	n = 16 males<br>24 ± 3.1 years old	Biceps femoris,<br>rectus femoris	Squat (60°),<br>Forward lunge.	Wobble board,<br>Swiss ball,<br>Bosu up,<br>Bosu down,<br>Dyna disc.	Floor.	↔ EMG activity (all muscles)
(SAETERBAKKEN; FIMLAND, 2013)	n = 15 males<br>23.3 6 ±2.7 years old	Rectus femoris,<br>vastus medialis,<br>vastus lateralis,<br>biceps femoris,	Half squat (90°).	Power board,<br>Bosu ball,<br>balance cone.	Floor.	↔ EMG activity (vastus medialis, vastus lateralis, biceps femoris)<br><br>↑ Stable surface (rectus femoris)
(MARSHALL; MURPHY, 2006)	n = 8 males; 4 females<br>22.1 ± 2.4 years old	Vastus lateralis,<br>biceps femoris.	Half squat (90°) ISO.	Swiss ball.	Stable surface.	↔ EMG activity (vastus lateralis, biceps femoris)

Table 2. ***(continued)***

(HYONG; KANG, 2013)	n = 5 males; 9 females 21.4 years old	Vastus medialis, vastus lateralis.	Squat (60°).	Foam pads, Rubber air discs.	Hard plate.	↑ Unstable surface (all muscles)
(JEON et al., 2020)	n = 23 males; 17 females 20.8±1.43 years old	Rectus femoris, vastus medialis, vastus lateralis.	Half squat (90°).	Bosu ball, Swiss ball.	Floor.	↔ EMG activity (all muscles)
(KANG et al., 2017)	n = 24 males 26.04 ± 2.19 years old	vastus medialis, vastus lateralis.	Squat (15º) ISO, Squat (45º) ISO, Squat (60º) ISO.	Aerostep.	Stable surface.	**Squat (15º) ISO** ↑ Unstable surface (vastus medialis) ↔ EMG activity (vastus lateralis) **Squat (45º) ISO** ↔ EMG activity (all muscles) **Squat (60º) ISO** ↑ Stable surface (vastus medialis) ↔ EMG activity (vastus lateralis)
(MCBRIDE; CORMIE; DEANE, 2006)	n = 9 males 22.4 ± 2.7 years old	Vastus medialis, vastus lateralis, biceps femoris.	Deep squat (100°).	Inflated balance disk.	Force plate.	↑ Stable surface (vastus medialis, lateralis) ↔ EMG activity (biceps femoris)
(ARANDA et al., 2016)	n = 19 males 24.65 ± 3.48 years old	Biceps femoris, vastus lateralis.	Squat.	Balance disc.	Floor.	↔ EMG activity (all muscles)
(MARÍN; HAZELL, 2014)	n = 23 males; 5 females 21.7 ± 1.3 years old	Vastus medialis, vastus lateralis.	Squat (60°).	Wobble board. Vibration platform.	Floor.	↑ Unstable surface-vibration platform 30 Hz (vastus medialis) ↔ EMG activity (vastus lateralis)
(PARK et al., 2015)	n = 12 males; 13 females 25.19 ± 2.48 years old	Vastus medialis, vastus lateralis.	Squat (60°) (Vision Allowed).	Foam pad.	Floor.	**Vision Allowed** ↑ Unstable surface (all muscles)
(AGUILERA-CASTELLS et al., 2019)	n = 20 males 24.40 ± 3.63 years old	Rectus femoris, biceps femoris, vastus medialis, vastus lateralis.	Bulgarian squat.	Bosu ball, TRX.	Floor.	↑ Unstable surface (rectus femoris) ↔ EMG activity (vastus lateralis, vastus medialis, biceps femoris)
(KIM et al., 2013)	n = 11 males; 11 females 23.5 ± 4.92 years old	Ipsilateral gluteus maximus, ipsilateral biceps femoris.	Prone hip extension.	Foam roll.	Floor.	↑ Unstable surface (ipsilateral gluteus maximus) ↔ EMG activity (ipsilateral biceps femoris)
(MILLER et al., 2019)	n = 9 males; 10 females 21.45± 1.8 years old	Rectus femoris, gluteus maximus	Split squat.	TRX.	Stable bench.	↑ Unstable surface (gluteus maximus) ↔ EMG activity (rectus femoris)
(KRAUSE et al., 2018)	n = 15 males;15 females 23.9 ± 1.7 years old	Rectus femoris, hamstring, gluteus maximus.	Standart lunge.	TRX.	Floor.	↑ Unstable surface (hamstring, gluteus maximus) ↔ EMG activity (rectus femoris)

Table 2. ***(continued)***

(BOUILLON et al., 2019)	n = 7 males; 13 females<br>23.4 ± 1.47 years old	Rectus femoris,<br>vastus medialis,<br>biceps femoris.	Standart lunge.	Stepright,<br>Bosu.	Firm surface.	↔ EMG activity (biceps femoris, rectus femoris, vastus medialis)<br><br>However, the Stepright device showed ↑ EMG activity when compared to the Bosu ball, but with no difference for the stable surface (rectus femoris, vastus medialis).
(LEE; CHOI; KIM, 2017)	n = 15 males<br>22.0 ± 2.0 years old	Vastus lateralis,<br>vastus medialis,<br>rectus femoris.	Standing position with extension of the knee joint.	Aero step (the plate that could be tilted at 0° and 20°)	Floor (the plate that could be tilted at 0° and 20°).	**Not Inclined board 0º**<br>↔ EMG activity (all muscles)<br><br>**Inclined board 20º**<br>↑ Unstable surface (vastus lateralis, vastus medialis, rectus femoris)
(BEHM; ANDERSON; CURNEW, 2002)	n = 8 males<br>24.3 ± 6.7 years old	Quadriceps,<br>soleus.	Unilateral knee extension, seated.<br><br>Unilateral plantar flexion, seated.	Swiss ball	Bench.	**Unilateral knee extension seated.**<br>↑ Stable surface (quadriceps).<br><br>**Unilateral plantar flexion seated.**<br>↑ Unstable surface (soleus)
(BUSCÀ et al., 2020)	n = 5 males; 9 females<br>20.2 ± 1.54 years old	Vastus medialis,<br>vastus lateralis,<br>biceps femoris.	Squat.	Foam,<br>Bosu-up,<br>Bosu-down.	Floor.	↑ Unstable surface—Bosu-up (vastus medialis)<br><br>↑ Unstable surface—Bosu-down (vastus lateralis)<br><br>↑ Unstable surface—Bosu-down and Bosu-up (biceps femoris)
(KIM et al., 2021)	n = 22 males<br>22.5 ± 2.7 years old	Rectus femoris,<br>vastus lateralis,<br>vastus medialis,<br>biceps femoris,<br>semitendinosus,<br>semimembranosus.	Squat,<br>wall squat,<br>Spanish squat.	Swiss ball,<br>Bosu ball.	Floor.	**Squat and Wall squat**<br>↔ EMG activity (all muscles)<br><br>**Spanish squat**<br>↔ EMG activity (rectus femoris, vastus lateralis, vastus medialis, biceps femoris, semimembranosus)<br>↑ Unstable surface (semitendinosus)
(GÜNDOĞAN; AYDIN; SAĞLAM, 2023)	n = 14 males<br>20.4 ± 1.2 years old	Vastus medialis,<br>vastus lateralis	Squat	Gymnastics mat,<br>Bosu ball	Stable surface.	↔ EMG activity (all muscles)
(MAIOR et al., 2009)	n = 20 males<br>25 ± 3 years old	Rectus femoris,<br>vastus lateralis,<br>vastus medialis	Squat	Core Board Training®	Floor	↑ Unstable surface (all muscles)

Legends: ISO – Isometric.

Table 3. Summary of the characteristics and results of the selected studies to upper limbs muscles.

Authors	Sample	Muscles	Exercise	Unstable surface	Stable surface	Main Results
(JEONG; CHUNG; SHIM, 2014)	n = 10 males; 18 females<br>23.67 ± 2.67 years old	Anterior deltoid.	Push up plus	Slings.	Stable surface.	↑ Unstable surface (anterior deltoid)
(LEHMAN et al., 2006)	n = 13 males<br>26.3 ± 1.5 years old	Pectoralis major, triceps brachii.	Push up,<br>Push up plus.	Swiss ball.	Bench.	**Push up with the support of the devices on the hands.**<br>↑ Unstable surface (triceps brachii)<br>↔ EMG activity (pectoralis major)<br><br>**Push up with the support of the devices on the feet.**<br>↔ EMG activity (pectoralis major, triceps brachii)<br><br>**Push up plus**<br>↑ Unstable surface (triceps brachii)<br>↔ EMG activity (pectoralis major)
(TORRES et al., 2017)	n = 20 males<br>20.9 ± 1.8 years old	Anterior deltoid, triceps brachii, pectoralis major.	Push up plus.	Bosu ball.	Floor.	↑ Stable surface (anterior deltoid)<br><br>↔ EMG activity (triceps brachii, pectoralis major)
(SYED-ABDUL et al., 2018)	n = 69 females<br>Range 18-24 years old	Anterior deltoid, triceps brachii.	Push up.	Suspension tape at a standardized height of 213.4 cm, with handles approximately 7.5-12.5 cm above the floor.	Floor.	↑ Unstable surface (triceps brachii) in soccer players group<br>↔ EMG activity (triceps brachii) in gymnasts and sedentary group<br><br>↑ Unstable surface (anterior deltoid) in soccer players and gymnasts group<br>↔ EMG activity (anterior deltoid) in sedentary group
(HWANGBO; KIM, 2012)	n = 39 males; 6 females<br>25.3 ±2.2 years old	Pectoralis major, triceps brachii.	Push up.	Swiss ball.	Table.	**Push up with dorsiflexion.**<br>↑ Unstable surface (pectoralis major)<br>↔ EMG activity (triceps brachii)<br><br>**Push up with plantarflexion.**<br>↔ EMG activity (pectoralis major, triceps brachii)
(PARK et al., 2013)	n = 16 males<br>24-26 years old	Triceps brachii.	Push up.	Wobble board.	Stable surface.	↑ Unstable surface (triceps brachii)
(CALATAYUD et al., 2014a)	n = 29 males<br>2.5 ± 2.0 years old	Pectoralis major, anterior deltoid, triceps brachii.	Push up.	TRX.	Floor.	↑ Unstable surface—Suspended push up with open eyes (pectoralis major, triceps brachii)<br><br>↑ Stable surface (anterior deltoid)
(MARSHALL; MURPHY, 2006a)	n = 8 males; 4 females<br>22.1 ± 2.4 years old	Pectoralis major, triceps brachii	Push up.	Swiss ball.	Stable surface.	↔ EMG activity (pectoralis major)<br>↑ Unstable surface (triceps brachii)
(DE ARAÚJO et al., 2011)	n = 12 males<br>22 ± 3 years old	Pectoralis major, triceps brachii.	One-arm knee press up.	Swiss ball.	Stable wooden stand.	↑ Unstable surface<br>(Pectoralis major, triceps brachii)

Table 3. ***(continued).***

(BEZERRA et al., 2020)	n = 10 males<br>26 ± 5 years old	Pectoralis major,<br>triceps brachii,<br>anterior deltoid.	Push up.	Swiss ball.	Floor.	↔ EMG activity (pectoralis major)<br><br>↑ Stable surface (anterior deltoid)<br><br>↑ Unstable surface (triceps brachii)
(DE OLIVEIRA; DE MORAIS CARVALHO; DE BRUM, 2008)	n = 20 males<br>23 ± 7 years old	Anterior deltoid,<br>pectoralis major.	Wall-press,<br>push-up,<br>bench-press.	Medicine ball.	Stable surface.	**Wall-press**<br>↑ Unstable surface (anterior deltoid)<br>↔ EMG activity (pectoralis major)<br><br>**Push-up**<br>↑ Unstable surface (anterior deltoid)<br>↑ Stable surface (pectoralis major)<br><br>**Bench-press**<br>↑ Unstable surface (anterior deltoid)<br>↔ EMG activity (pectoralis major)
(SANDHU; MAHAJAN; SHENOY, 2008)	n = 35 males<br>Range 20-30 years old	Pectoralis major,<br>triceps brachii.	Push-up,<br>knee push-up,<br>rlbow push-up,<br>eall press.	Swiss ball.	Stable surface.	**Push-up (concentric phase)**<br>↑ Unstable surface (pectoralis major, triceps brachii)<br><br>**Knee push-up (concentric phase)**<br>↑ Unstable surface (pectoralis major, triceps brachii)<br><br>**Elbow push-up (concentric phase)**<br>↔ EMG activity (pectoralis major, triceps brachii)<br><br>**Wall press (concentric phase)**<br>↔ EMG activity (triceps brachii)<br>↑ Unstable surface (pectoralis major)
(BORREANI et al., 2015b)	n = 29 males<br>23.5 ± 3.1 years old	Triceps brachii,<br>anterior deltoid,<br>pectoralis major.	Push-ups with their hands at 2 different heights (10 cm and 65 cm from the floor).	TRX.	Box,<br>bar in a Smith machine.	**Exercise 10 cm from the floor**<br>↔ EMG activity (pectoralis major)<br>↑ Unstable surface (triceps brachii)<br>↑ Stable surface (anterior deltoid)<br><br>**Exercise 65 cm from the floor**<br>↔ EMG activity (pectoralis major)<br>↑ Unstable surface (triceps brachii)<br>↑ Stable surface (anterior deltoid)
(BORREANI et al., 2015a)	n = 30 males<br>23 ± 1.13 years old	Anterior deltoid.	Push-ups.	Wobble board,<br>Stability disc,<br>Bosu ball,<br>TRX Suspension Trainer.	Floor.	↔ EMG activity (anterior deltoid)

Table 3. *(continued).*

(CALATAYUD et al., 2014b)	n = 29 males<br>23.5 ± 3.1 years old	Triceps brachii,<br>anterior deltoid,<br>pectoralis major.	Push-Ups.	TRX Suspension Trainer,<br>Jungle Gym XT,<br>Flying,<br>AirFit Trainer Pro.	Floor.	↑ Unstable surface— AirFit Trainer Pro (triceps brachii)<br><br>↑ Unstable surface— Jungle Gym XT (clavicular pectoralis)<br><br>↑ Stable surface (anterior deltoid), but no difference to Jungle Gym XT
(SNARR; ESCO, 2013)	n = 15 males; 6 females<br>25.24 ± 3.42 years old	Pectoralis major,<br>anterior deltoid,<br>triceps brachii.	Push-up.	TRX.	Floor.	↑ Unstable surface (all muscles)
(HERRINGTON; WATERMAN; SMITH, 2015)	n = 10 males; 11 females<br>22.8 ± 1.4 years old	Anterior deltoid,<br>pectoralis major.	One-arm knee press up,<br>one-arm press up,<br>two-arm press up.	Airex pad,<br>Swiss ball.	Floor.	**One-arm knee press up**<br>↔ EMG activity (pectoralis major, anterior deltoid)<br><br>**One-arm press up**<br>↔ EMG activity (pectoralis major, anterior deltoid)<br><br>**Two-arm press up**<br>↔ EMG activity (pectoralis major)<br>↑ Stable surface (anterior deltoid)
(PONTILLO et al., 2007)	n = 10 males; 5 females<br>30 ± 6 years old	Anterior deltoid,<br>pectoralis major,<br>triceps brachii.	One-arm knee press up.	Thera-Band® stability trainer.	Force platform.	↑ Stable surface (anterior deltoid)<br><br>↑ Unstable surface (triceps brachii)<br><br>↔ EMG activity (pectoralis major)
(DE MEY et al., 2014)	n = 26 males; 21 females<br>22 ± 4.31 years old	Pectoralis major,<br>anterior deltoid,<br>latissimus dorsi,<br>posterior deltoid,<br>middle trapezius.	Half push-up,<br>knee push-up,<br>knee prone bridging plus,<br>pull-up.	Redcord slings.	Bar,<br>floor.	**Half push-up**<br>↑ Unstable surface (pectoralis major)<br>↔ EMG activity (anterior deltoid)<br><br>**Knee push-up**<br>↑ Stable surface (anterior deltoid)<br>↔ EMG activity (pectoralis major)<br><br>**Knee prone bridging plus**<br>↑ Stable surface (anterior deltoid)<br>↑ Unstable surface (pectoralis major)<br><br>**Pull-up**<br>↑ Stable surface (middle trapezius, lower trapezius)<br>↑ Unstable surface (latissimus dorsi)<br>↔ EMG activity (posterior deltoid)
(ANDERSON et al., 2013)	n = 10 males; 5 females<br>29.3 ± 6.4 years old	Triceps brachii.	Push up.	Extreme balance board,<br>stability ball.	Floor.	↑ Unstable surface – Dual instability (triceps brachii)

Table 3. ***(continued).***

(YOUDAS et al., 2020)	n = 13 males; 13 females<br>24.2 ± 3.6 years old	Biceps brachii,<br>latissimus dorsi,<br>posterior deltoid,<br>middle trapezius.	Inverted row.	Bosu ball.	Floor.	↔ EMG activity (all muscles)
(KIM et al., 2012)	n = 33 (does not inform the gender of the participants)<br>21.61 ± 1.32 years old	Pectoralis major,<br>triceps brachii.	Push up.	Swiss ball.	Bench.	**Supports in the ankle region**<br>↑ Unstable surface (all muscles)<br><br>**Supports in the knee region**<br>↑ Unstable surface (all muscles)
(PARK; YOO, 2013)	n = 14 males<br>22 ± 2 years old	Pectoralis major,<br>triceps brachii.	Push up.	Wobble board.	Stable surface.	↑ Unstable surface (Pectoralis major)<br><br>↔ EMG activity<br>(triceps brachii)
(PATTERSON et al., 2015)	n = 15 males; 7 females<br>22.4 ± 5.0 years old	Pectoralis major.	Unilateral chest press,<br>bilateral chest press.	COR bench.	Flat bench.	**Unilateral chest press**<br>↔EMG activity (pectoralis major)<br><br>**Bilateral chest press**<br>↔EMG activity (pectoralis major)
(ANDERSON; BEHM, 2004)	n = 10 males<br>26.3 ± 6.0 years old	Pectoralis major,<br>anterior deltoid,<br>triceps brachii.	Chest press.	Swiss ball.	Bench.	**Dynamic condition**<br>↔ EMG activity (all muscles)<br><br>**Isometric condition**<br>↔ EMG activity (all muscles)
(MARSHALL; MURPHY, 2006b)	n = 9 males; 5 females<br>23.6 ± 1.6 years old	Anterior deltoid,<br>triceps brachii,<br>pectoralis major.	Chest press.	Swiss ball.	Bench.	↑ Unstable surface (anterior deltoid)<br>↔ EMG activity (triceps brachii, pectoralis major)
(SAETERBAKKEN; FIMLAND, 2013)	n = 16 males<br>22.5 ± 2.0 years old	Pectoralis major,<br>anterior deltoid,<br>triceps brachii.	Bench press.	Swiss ball,<br>balance cushion.	Bench.	**Swiss ball**<br>↓ Unstable surface (pectoralis major, triceps brachii)<br>↔ EMG activity (anterior deltoid)<br><br>**Balance cushion**<br>↓ Unstable surface (triceps brachii)<br>↔ EMG activity (anterior deltoid, pectoralis major)
(URIBE et al., 2010)	n = 16 males<br>24.1 ± 2.1 years old	Anterior deltoid,<br>pectoralis major.	Chest press,<br>shoulder press.	Swiss ball.	Bench.	**Chest press**<br>↔ EMG activity (all muscles)<br><br>**Shoulder press**<br>↔ EMG activity (all muscles)
(PALMA et al., 2021)	n = 12 males<br>23.7 ± 3.0 years old	Pectoralis major.	Push up.	Bosu,<br>balance disc,<br>TRX.	Floor.	↔ EMG activity (Pectoralis major)

Table 3. ***(continued).***

(KOHLER; FLANAGAN; WHITING, 2010)	n = 24 males; 6 females<br>30 ± 8 years old	Anterior deltoid, middle deltoid, upper trapezius, triceps brachii.	Shoulder press with bar, shoulder press with dumbbells.	Swiss ball.	Bench.	**Shoulder press with bar**<br>↔ EMG activity (anterior deltoid, middle deltoid, upper trapezius)<br>↑ Stable surface (triceps brachii)<br><br>**Shoulder press with dumbbells**<br>↔ EMG activity (anterior deltoid, middle deltoid, upper trapezius)<br>↑ Stable surface (triceps brachii)
(NAIRN; SUTHERLAND; DRAKE, 2015)	n = 10 males<br>21 ± 3 years old	Pectoralis major, triceps brachii, anterior deltoid.	Bench press.	Tube,<br>Swiss ball.	Bench.	↑ Stable surface – When performed with the barbell in the concentric phase (pectoralis major, triceps brachii)<br><br>↑ Unstable surface - When performed on the Swiss ball for the concentric phase (anterior deltoid)
(ARANDA et al., 2016)	n = 19 males<br>24.65 ± 3.48 years old	Anterior deltoid, pectoralis major.	Bench press.	Swiss ball,<br>Balance disk.	Bench,<br>floor.	↔ EMG activity (all muscles)
(NASCIMENTO et al., 2017)	n = 20 males<br>20.9 ± 1.8 years old	Anterior deltoid, pectoralis major, biceps brachii, triceps brachii.	Horizontal dumbbell fly, bench press.	Proprioceptive disc.	Bench.	**Horizontal Dumbbell Fly**<br>↑ Unstable surface (anterior deltoid, biceps brachii, pectoralis major)<br><br>**Bench press**<br>↑ Unstable surface (pectoralis major)<br>↔ EMG activity (anterior deltoid, triceps brachii).
(REISER et al., 2017)	n = 17 males<br>26 ± 6.4 years old	Anterior deltoid, pectoralis major.	Horizontal dumbbell fly.	Swiss ball.	Bench.	↔ EMG activity (all muscles)
(MELO et al., 2014)	n = 14 males<br>22,5 ± 2,4 years old	Pectoralis major, anterior deltoid.	Horizontal dumbbell fly.	Swiss ball.	Flat bench.	↑ Unstable surface (all muscles)
(SOUSA et al., 2022)	n = 28 males<br>24.6 ± 3.5 years old	Anterior deltoid, biceps brachii, pectoralis major, triceps brachii.	Horizontal dumbbell fly, bench press.	Swiss ball.	Flat bench.	**Horizontal Dumbbell Fly**<br>Control group<br>↔ EMG activity (all muscles)<br>Dyskinesis group<br>↔ EMG activity (all muscles)<br><br>**Bench press**<br>Control group<br>↑ Stable surface (anterior deltoid)<br>↔ EMG activity (pectoralis major, triceps brachii)<br>Dyskinesis group<br>↔ EMG activity (all muscles)
