# Supplementary material for: Comparison of the Electromyography Activity during Exercises with Stable and Unstable Surfaces: A Systematic Review and Meta-Analysis
Source: Sports (Basel). 2024 Apr 18;12(4):111. doi: 10.3390/sports12040111 (PMC11055131; doi:10.3390/sports12040111)
Supplement: Supplementary file 1 [file sports-12-00111-s001.zip › Supplementary Online Material S2.pdf]

Title: Comparison of the electromyography activity during exercises with stable and unstable surfaces: A systematic review and meta-analysis

<u>Supplementary Online Material S2</u>

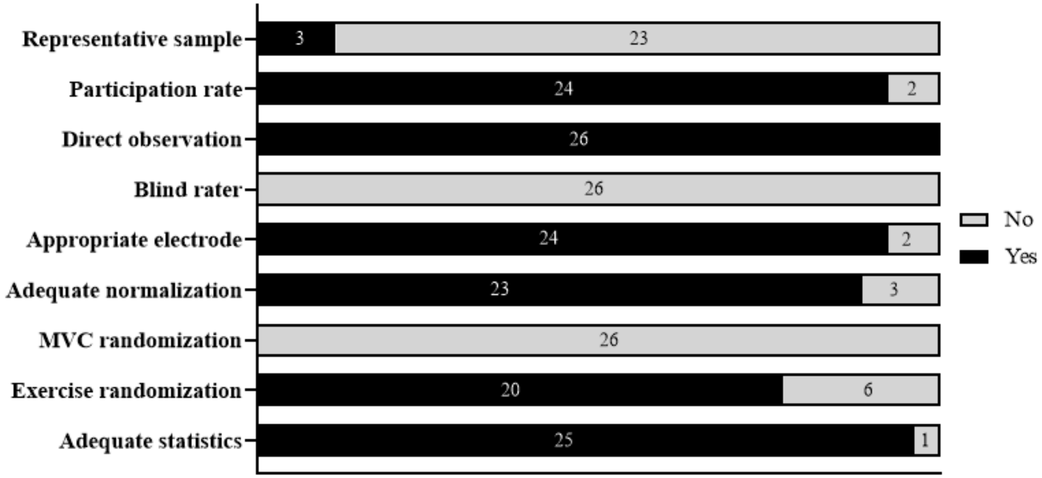

Figure 1. Methodological quality of the studies that evaluated the core musculature.

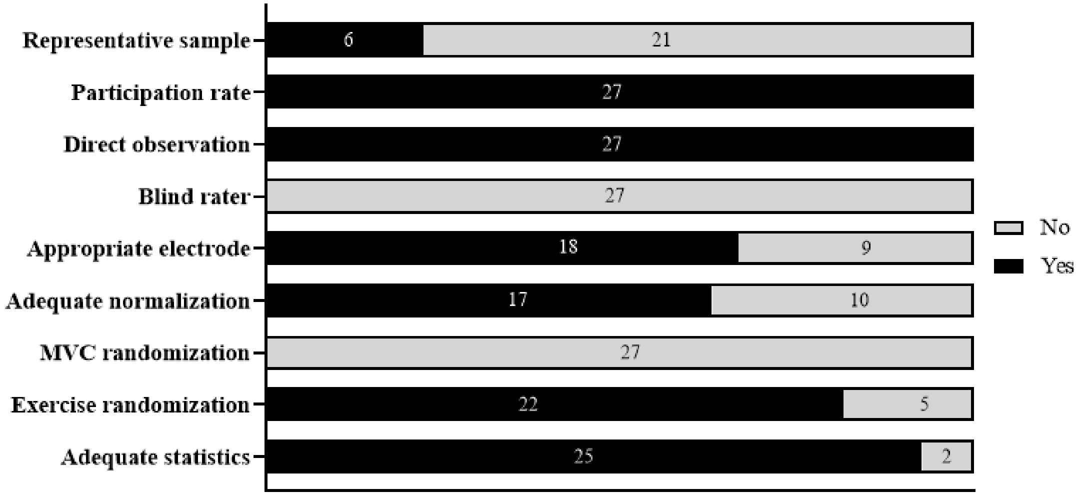

Figure 2. Methodological quality of the studies that evaluated the lower limbs musculature.

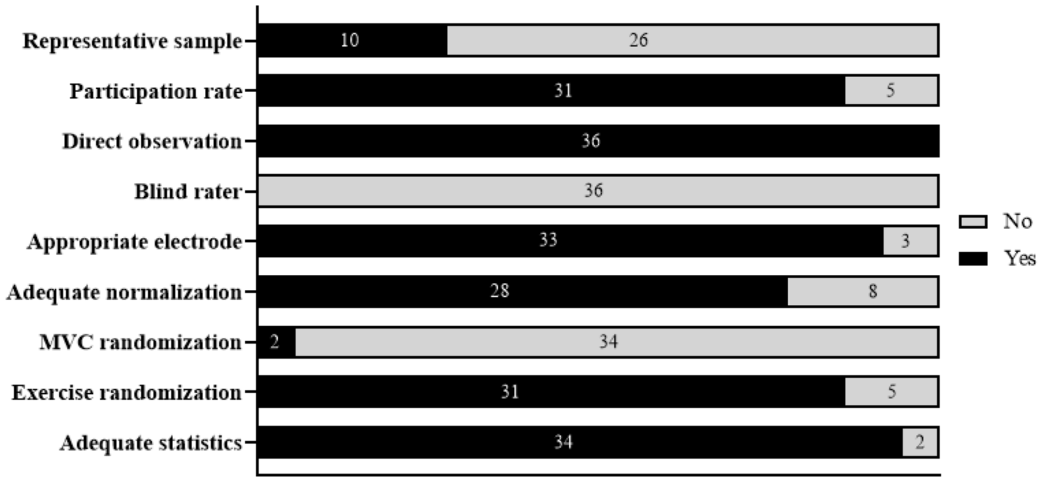

Figure 3. Methodological quality of the studies that evaluated the upper limbs musculature.
