# Supplementary material for: Comparison of the Electromyography Activity during Exercises with Stable and Unstable Surfaces: A Systematic Review and Meta-Analysis
Source: Sports (Basel). 2024 Apr 18;12(4):111. doi: 10.3390/sports12040111 (PMC11055131; doi:10.3390/sports12040111)
Supplement: Supplementary file 1 [file sports-12-00111-s001.zip › Supplementary Online Material S3.pdf]

Title: Comparison of the electromyography activity during exercises with stable and unstable surfaces: A systematic review and meta-analysis

Supplementary Online Material S3

Table 1. Evaluation of the quality of the selected studies with evaluations of internal validity and external validity of core muscles.

No/Author/year	External Validity		Internal Validity							
			Performance	Detection	Selection Bias					
	Representative sample	Participation rate	Direct observation	Blind rater	Appropriate electrode	Adequate normalization	MVC randomization	Exercise randomization	Adequate statistics	Total score
(BEHM et al., 2005)	No	Yes	Yes	No	Yes	Yes	No	Yes	Yes	6
(IMAI et al., 2010)	No	Yes	Yes	No	Yes	Yes	No	No	Yes	5
(KIM; KIM; CHUNG, 2014)	No	Yes	Yes	No	Yes	Yes	No	No	Yes	5
(HA et al., 2015)	No	Yes	Yes	No	Yes	Yes	No	Yes	Yes	6
(KIM et al., 2018)	No	Yes	Yes	No	Yes	Yes	No	Yes	Yes	6
(SUNDSTRUP et al., 2012)	No	Yes	Yes	No	Yes	Yes	No	Yes	Yes	6
(LEHMAN et al., 2005)	No	Yes	Yes	No	Yes	Yes	No	No	Yes	5
(YOUDAS et al., 2015)	Yes	Yes	Yes	No	Yes	Yes	No	Yes	Yes	7
(YOUDAS et al., 2017)	Yes	Yes	Yes	No	Yes	Yes	No	Yes	Yes	7
(VILAÇA-ALVES et al., 2016)	No	Yes	Yes	No	Yes	No	No	Yes	Yes	5
(SNARR; ESCO, 2014)	No	Yes	Yes	No	Yes	Yes	No	Yes	Yes	6
(ATKINS et al., 2015)	No	Yes	Yes	No	Yes	Yes	No	Yes	Yes	6
(STERNLICHT et al., 2007)	No	Yes	Yes	No	Yes	No	No	Yes	Yes	5
(FELDWIESER et al., 2012)	No	Yes	Yes	No	Yes	Yes	No	Yes	Yes	6
(KIM; OH, 2015)	No	Yes	Yes	No	Yes	Yes	No	Yes	Yes	6
(LEE; PARK; LEE, 2015)	No	Yes	Yes	No	No	Yes	No	Yes	Yes	5
(SAETERBAKKEN et al., 2014)	No	Yes	Yes	No	Yes	No	No	Yes	Yes	5
(BYRNE et al., 2014)	No	No	Yes	No	Yes	Yes	No	Yes	Yes	5
(YOON et al., 2018)	No	Yes	Yes	No	Yes	Yes	No	Yes	Yes	6
(KIM et al., 2011)	No	Yes	Yes	No	Yes	Yes	No	Yes	Yes	6
(MARSHALL; MURPHY, 2006)	Yes	Yes	Yes	No	Yes	Yes	No	Yes	Yes	7
(ANDRADE et al., 2015)	No	Yes	Yes	No	Yes	Yes	No	No	Yes	5
(BISCARINI; CONTEMORI; GROLLA, 2019)	No	Yes	Yes	No	No	Yes	No	Yes	Yes	5
(CZAPROWSKI et al., 2014)	No	Yes	Yes	No	Yes	Yes	No	Yes	Yes	6
(LEE et al., 2017)	No	No	Yes	No	Yes	Yes	No	No	No	3
(LUK et al., 2021)	No	Yes	Yes	No	Yes	Yes	No	No	Yes	5

Table 2. Evaluation of the quality of the selected studies with evaluations of internal validity and external validity of lower limbs muscles.

Author/year	External Validity		Internal Validity							
			Performance	Detection	Selection Bias					
	Representative sample	Participation rate	Direct observation	Blind rater	Appropriate electrode	Adequate normalization	MVC randomization	Exercise randomization	Adequate statistics	Total score
(ANDERSON; BEHM, 2005)	No	Yes	Yes	No	Yes	No	No	Yes	Yes	5
(LEE; CHOI; KIM, 2017)	No	Yes	Yes	No	No	Yes	No	No	Yes	4
(HAN et al., 2017)	No	Yes	Yes	No	No	No	No	No	Yes	3
(LI; CAO; CHEN, 2013)	No	Yes	Yes	No	Yes	No	No	Yes	Yes	5
(WAHL; BEHM, 2008)	No	Yes	Yes	No	Yes	No	No	Yes	Yes	5
(SAETERBAKKEN; FIMLAND, 2013)	No	Yes	Yes	No	Yes	No	No	Yes	Yes	5
(BEHM; ANDERSON; CURNEW, 2002)	Yes	Yes	Yes	No	Yes	No	No	No	Yes	5
(AGUILERA-CASTELLS et al., 2019)	Yes	Yes	Yes	No	Yes	Yes	No	Yes	Yes	7
(ANDERSEN et al., 2014)	No	Yes	Yes	No	Yes	Yes	No	Yes	Yes	6
(NAIRN; SUTHERLAND; DRAKE, 2017)	No	Yes	Yes	No	Yes	Yes	No	Yes	Yes	6
(KRAUSE et al., 2018b)	Yes	Yes	Yes	No	Yes	Yes	No	Yes	Yes	7
(ARANDA et al., 2016)	No	Yes	Yes	No	Yes	Yes	No	Yes	Yes	6
(MARÍN; HAZELL, 2014)	No	Yes	Yes	No	Yes	Yes	No	Yes	Yes	6
(PARK et al., 2015)	No	Yes	Yes	No	No	No	No	No	Yes	3
(MCBRIDE et al., 2010)	No	Yes	Yes	No	Yes	No	No	Yes	Yes	5
(KIM et al., 2013)	No	Yes	Yes	No	Yes	Yes	No	Yes	Yes	6
(MILLER et al., 2019)	Yes	Yes	Yes	No	Yes	Yes	No	Yes	Yes	7
(BOUILLON et al., 2019)	No	Yes	Yes	No	Yes	Yes	No	Yes	Yes	6
(MARSHALL; MURPHY, 2006)	Yes	Yes	Yes	No	Yes	Yes	No	Yes	Yes	7
(HYONG; KANG, 2013)	No	Yes	Yes	No	No	Yes	No	Yes	Yes	5
(JEON et al., 2020)	No	Yes	Yes	No	No	No	No	No	Yes	3
(KANG et al., 2017)	No	Yes	Yes	No	No	Yes	No	Yes	No	4
(MCBRIDE; CORMIE; DEANE, 2006)	No	Yes	Yes	No	No	Yes	No	Yes	Yes	5
(BUSCÀ et al., 2020)	Yes	Yes	Yes	No	No	No	No	Yes	Yes	5
(KIM et al., 2021)	No	Yes	Yes	No	Yes	Yes	No	Yes	Yes	6
(GÜNDOĞAN; AYDIN; SAĞLAM, 2023)	No	Yes	Yes	No	Yes	Yes	No	Yes	Yes	6
(MAIOR et al., 2009)	No	Yes	Yes	No	No	Yes	No	Yes	No	4

Table 3. Evaluation of the quality of the selected studies with evaluations of internal validity and external validity of upper limbs muscles.

Author/year	External Validity		Internal Validity							
			Performance	Detection	Selection Bias					
	Representative sample	Participation rate	Direct observation	Blind rater	Appropriate electrode	Adequate normalization	MVC randomization	Exercise randomization	Adequate statistics	Total score
(SOUSA et al., 2022)	Yes	Yes	Yes	No	Yes	Yes	Yes	Yes	Yes	8
(PALMA et al., 2021)	No	No	Yes	No	Yes	Yes	No	No	Yes	4
(SANDHU; MAHAJAN; SHENOY, 2008)	No	No	Yes	No	Yes	Yes	No	Yes	Yes	5
(BORREANI et al., 2015b)	Yes	Yes	Yes	No	Yes	Yes	No	Yes	Yes	7
(CALATAYUD et al., 2014b)	Yes	Yes	Yes	No	Yes	Yes	No	Yes	Yes	7
(BORREANI et al., 2015a)	Yes	Yes	Yes	No	Yes	Yes	No	Yes	Yes	7
(SNARR; ESCO, 2013)	No	Yes	Yes	No	Yes	Yes	No	Yes	Yes	6
(HERRINGTON; WATERMAN; SMITH, 2015)	No	Yes	Yes	No	Yes	Yes	No	No	Yes	5
(PONTILLO et al., 2007)	No	Yes	Yes	No	Yes	Yes	No	Yes	Yes	6
(DE ARAÚJO et al., 2011)	No	Yes	Yes	No	Yes	Yes	No	Yes	Yes	6
(KIM et al., 2012)	No	Yes	Yes	No	No	No	No	No	Yes	3
(PARK; YOO, 2013)	No	Yes	Yes	No	Yes	Yes	No	Yes	No	5
(ANDERSON et al., 2013)	No	Yes	Yes	No	Yes	No	No	No	Yes	4
(PATTERSON et al., 2015)	No	No	Yes	No	Yes	No	No	Yes	Yes	4
(ANDERSON; BEHM, 2004)	No	Yes	Yes	No	Yes	No	No	Yes	Yes	5
(MARSHALL; MURPHY, 2006b)	Yes	Yes	Yes	No	Yes	No	No	Yes	Yes	6
(SAETERBAKKEN; FIMLAND, 2013)	Yes	No	Yes	No	Yes	No	No	Yes	Yes	5
(NAIRN; SUTHERLAND; DRAKE, 2015)	No	Yes	Yes	No	Yes	Yes	No	Yes	Yes	6
(ARANDA et al., 2016)	No	Yes	Yes	No	Yes	Yes	No	Yes	Yes	6
(REISER et al., 2017)	Yes	No	Yes	No	Yes	Yes	No	Yes	Yes	6
(MELO et al., 2014)	No	Yes	Yes	No	Yes	Yes	Yes	Yes	Yes	7
(NASCIMENTO et al., 2017)	No	Yes	Yes	No	Yes	Yes	No	Yes	No	5
(KOHLER; FLANAGAN; WHITING, 2010)	Yes	Yes	Yes	No	Yes	No	No	Yes	Yes	6
(MARSHALL; MURPHY, 2006)	Yes	Yes	Yes	No	Yes	Yes	No	Yes	Yes	7
(JEONG; CHUNG; SHIM, 2014)	No	Yes	Yes	No	Yes	Yes	No	No	Yes	5
(LEHMAN et al., 2006)	No	Yes	Yes	No	Yes	Yes	No	Yes	Yes	6
(TORRES et al., 2017)	No	Yes	Yes	No	Yes	Yes	No	Yes	Yes	6

Table 3. ***(continued)***

(SYED-ABDUL et al., 2018)	Yes	Yes	Yes	No	Yes	No	No	Yes	Yes	6
(HWANGBO; KIM, 2012)	No	Yes	Yes	No	No	Yes	No	Yes	Yes	5
(PARK et al., 2013)	No	Yes	Yes	No	Yes	Yes	No	Yes	Yes	6
(CALATAYUD et al., 2014a)	No	Yes	Yes	No	Yes	Yes	No	Yes	Yes	6
(BEZERRA et al., 2020)	No	Yes	Yes	No	Yes	Yes	No	Yes	Yes	6
(DE OLIVEIRA; DE MORAIS; DE BRUM, 2008)	No	Yes	Yes	No	Yes	Yes	No	Yes	Yes	6
(URIBE et al., 2010)	No	Yes	Yes	No	Yes	Yes	No	Yes	Yes	6
(YOUDAS et al., 2020)	No	Yes	Yes	No	Yes	Yes	No	Yes	Yes	6
(DE MEY et al., 2014)	No	Yes	Yes	No	No	Yes	No	Yes	Yes	5

Table 4. Evaluation of the quality of the selected studies with evaluations of internal validity and external validity of all muscles.

Author/year	External Validity		Internal Validity							
			Performance	Detection	Selection Bias					
	Representative sample	Participation rate	Direct observation	Blind rater	Appropriate electrode	Adequate normalization	MVC randomization	Exercise randomization	Adequate statistics	Total score
(BEHM et al., 2005)	No	Yes	Yes	No	Yes	Yes	No	Yes	Yes	6
(IMAI et al., 2010)	No	Yes	Yes	No	Yes	Yes	No	No	Yes	5
(KIM; KIM; CHUNG, 2014)	No	Yes	Yes	No	Yes	Yes	No	No	Yes	5
(HA et al., 2015)	No	Yes	Yes	No	Yes	Yes	No	Yes	Yes	6
(KIM et al., 2018)	No	Yes	Yes	No	Yes	Yes	No	Yes	Yes	6
(SUNDSTRUP et al., 2012)	No	Yes	Yes	No	Yes	Yes	No	Yes	Yes	6
(LEHMAN et al., 2005)	No	Yes	Yes	No	Yes	Yes	No	No	Yes	5
(YOUDAS et al., 2015)	Yes	Yes	Yes	No	Yes	Yes	No	Yes	Yes	7
(YOUDAS et al., 2017)	Yes	Yes	Yes	No	Yes	Yes	No	Yes	Yes	7
(VILAÇA-ALVES et al., 2016)	No	Yes	Yes	No	Yes	No	No	Yes	Yes	5
(SNARR; ESCO, 2014)	No	Yes	Yes	No	Yes	Yes	No	Yes	Yes	6
(ATKINS et al., 2015)	No	Yes	Yes	No	Yes	Yes	No	Yes	Yes	6
(STERNLICHT et al., 2007)	No	Yes	Yes	No	Yes	No	No	Yes	Yes	5
(FELDWIESER et al., 2012)	No	Yes	Yes	No	Yes	Yes	No	Yes	Yes	6
(KIM; OH, 2015)	No	Yes	Yes	No	Yes	Yes	No	Yes	Yes	6
(LEE; PARK; LEE, 2015)	No	Yes	Yes	No	No	Yes	No	Yes	Yes	5
(SAETERBAKKEN et al., 2014)	No	Yes	Yes	No	Yes	No	No	Yes	Yes	5
(BYRNE et al., 2014)	No	No	Yes	No	Yes	Yes	No	Yes	Yes	5
(YOON et al., 2018)	No	Yes	Yes	No	Yes	Yes	No	Yes	Yes	6
(KIM et al., 2011)	No	Yes	Yes	No	Yes	Yes	No	Yes	Yes	6
(MARSHALL; MURPHY, 2006)	Yes	Yes	Yes	No	Yes	Yes	No	Yes	Yes	7
(ANDRADE et al., 2015)	No	Yes	Yes	No	Yes	Yes	No	No	Yes	5
(BISCARINI; CONTEMORI; GROLLA, 2019)	No	Yes	Yes	No	No	Yes	No	Yes	Yes	5
(CZAPROWSKI et al., 2014)	No	Yes	Yes	No	Yes	Yes	No	Yes	Yes	6
(LEE et al., 2017)	No	No	Yes	No	Yes	Yes	No	No	No	3

Table 4. ***(continued)***

(LUK et al., 2021)	No	Yes	Yes	No	Yes	Yes	No	No	Yes	5
(ANDERSON; BEHM, 2005)	No	Yes	Yes	No	Yes	No	No	Yes	Yes	5
(LEE; CHOI; KIM, 2017)	No	Yes	Yes	No	No	Yes	No	No	Yes	4
(HAN et al., 2017)	No	Yes	Yes	No	No	No	No	No	Yes	3
(LI; CAO; CHEN, 2013)	No	Yes	Yes	No	Yes	No	No	Yes	Yes	5
(WAHL; BEHM, 2008)	No	Yes	Yes	No	Yes	No	No	Yes	Yes	5
(SAETERBAKKEN; FIMLAND, 2013)	No	Yes	Yes	No	Yes	No	No	Yes	Yes	5
(BEHM; ANDERSON; CURNEW, 2002)	Yes	Yes	Yes	No	Yes	No	No	No	Yes	5
(AGUILERA-CASTELLS et al., 2019)	Yes	Yes	Yes	No	Yes	Yes	No	Yes	Yes	7
(ANDERSEN et al., 2014)	No	Yes	Yes	No	Yes	Yes	No	Yes	Yes	6
(NAIRN; SUTHERLAND; DRAKE, 2017)	No	Yes	Yes	No	Yes	Yes	No	Yes	Yes	6
(KRAUSE et al., 2018b)	Yes	Yes	Yes	No	Yes	Yes	No	Yes	Yes	7
(ARANDA et al., 2016)	No	Yes	Yes	No	Yes	Yes	No	Yes	Yes	6
(MARÍN; HAZELL, 2014)	No	Yes	Yes	No	Yes	Yes	No	Yes	Yes	6
(PARK et al., 2015)	No	Yes	Yes	No	No	No	No	No	Yes	3
(MCBRIDE et al., 2010)	No	Yes	Yes	No	Yes	No	No	Yes	Yes	5
(KIM et al., 2013)	No	Yes	Yes	No	Yes	Yes	No	Yes	Yes	6
(MILLER et al., 2019)	Yes	Yes	Yes	No	Yes	Yes	No	Yes	Yes	7
(BOUILLON et al., 2019)	No	Yes	Yes	No	Yes	Yes	No	Yes	Yes	6
(HYONG; KANG, 2013)	No	Yes	Yes	No	No	Yes	No	Yes	Yes	5
(JEON et al., 2020)	No	Yes	Yes	No	No	No	No	No	Yes	3
(KANG et al., 2017)	No	Yes	Yes	No	No	Yes	No	Yes	No	4
(MCBRIDE; CORMIE; DEANE, 2006)	No	Yes	Yes	No	No	Yes	No	Yes	Yes	5
(BUSCÀ et al., 2020)	Yes	Yes	Yes	No	No	No	No	Yes	Yes	5
(KIM et al., 2021)	No	Yes	Yes	No	Yes	Yes	No	Yes	Yes	6
(SOUSA et al., 2022)	Yes	Yes	Yes	No	Yes	Yes	Yes	Yes	Yes	8
(PALMA et al., 2021)	No	No	Yes	No	Yes	Yes	No	No	Yes	4
(SANDHU; MAHAJAN; SHENOY, 2008)	No	No	Yes	No	Yes	Yes	No	Yes	Yes	5

Table 4. ***(continued)***

(BORREANI et al., 2015b)	Yes	Yes	Yes	No	Yes	Yes	No	Yes	Yes	7
(CALATAYUD et al., 2014b)	Yes	Yes	Yes	No	Yes	Yes	No	Yes	Yes	7
(BORREANI et al., 2015a)	Yes	Yes	Yes	No	Yes	Yes	No	Yes	Yes	7
(SNARR; ESCO, 2013)	No	Yes	Yes	No	Yes	Yes	No	Yes	Yes	6
(HERRINGTON; WATERMAN; SMITH, 2015)	No	Yes	Yes	No	Yes	Yes	No	No	Yes	5
(PONTILLO et al., 2007)	No	Yes	Yes	No	Yes	Yes	No	Yes	Yes	6
(DE ARAÚJO et al., 2011)	No	Yes	Yes	No	Yes	Yes	No	Yes	Yes	6
(KIM et al., 2012)	No	Yes	Yes	No	No	No	No	No	Yes	3
(PARK; YOO, 2013)	No	Yes	Yes	No	Yes	Yes	No	Yes	No	5
(ANDERSON et al., 2013)	No	Yes	Yes	No	Yes	No	No	No	Yes	4
(PATTERSON et al., 2015)	No	No	Yes	No	Yes	No	No	Yes	Yes	4
(ANDERSON; BEHM, 2004)	No	Yes	Yes	No	Yes	No	No	Yes	Yes	5
(MARSHALL; MURPHY, 2006b)	Yes	Yes	Yes	No	Yes	No	No	Yes	Yes	6
(SAETERBAKKEN; FIMLAND, 2013)	Yes	No	Yes	No	Yes	No	No	Yes	Yes	5
(NAIRN; SUTHERLAND; DRAKE, 2015)	No	Yes	Yes	No	Yes	Yes	No	Yes	Yes	6
(REISER et al., 2017)	Yes	No	Yes	No	Yes	Yes	No	Yes	Yes	6
(MELO et al., 2014)	No	Yes	Yes	No	Yes	Yes	Yes	Yes	Yes	7
(NASCIMENTO et al., 2017)	No	Yes	Yes	No	Yes	Yes	No	Yes	No	5
(KOHLER; FLANAGAN; WHITING, 2010)	Yes	Yes	Yes	No	Yes	No	No	Yes	Yes	6
(JEONG; CHUNG; SHIM, 2014)	No	Yes	Yes	No	Yes	Yes	No	No	Yes	5
(LEHMAN et al., 2006)	No	Yes	Yes	No	Yes	Yes	No	Yes	Yes	6
(TORRES et al., 2017)	No	Yes	Yes	No	Yes	Yes	No	Yes	Yes	6
(SYED-ABDUL et al., 2018)	Yes	Yes	Yes	No	Yes	No	No	Yes	Yes	6
(HWANGBO; KIM, 2012)	No	Yes	Yes	No	No	Yes	No	Yes	Yes	5
(PARK et al., 2013)	No	Yes	Yes	No	Yes	Yes	No	Yes	Yes	6
(CALATAYUD et al., 2014a)	No	Yes	Yes	No	Yes	Yes	No	Yes	Yes	6
(BEZERRA et al., 2020)	No	Yes	Yes	No	Yes	Yes	No	Yes	Yes	6
(DE OLIVEIRA; DE MORAIS; DE BRUM, 2008)	No	Yes	Yes	No	Yes	Yes	No	Yes	Yes	6

Table 4. ***(continued)***

(URIBE et al., 2010)	No	Yes	Yes	No	Yes	Yes	No	Yes	Yes	6
(YOUDAS et al., 2020)	No	Yes	Yes	No	Yes	Yes	No	Yes	Yes	6
(DE MEY et al., 2014)	No	Yes	Yes	No	No	Yes	No	Yes	Yes	5
(GÜNDOĞAN; AYDIN; SAĞLAM, 2023)	No	Yes	Yes	No	Yes	Yes	No	Yes	Yes	6
(MAIOR et al., 2009)	No	Yes	Yes	No	No	Yes	No	Yes	No	4
