# Supplementary material for: Comparison of the Electromyography Activity during Exercises with Stable and Unstable Surfaces: A Systematic Review and Meta-Analysis
Source: Sports (Basel). 2024 Apr 18;12(4):111. doi: 10.3390/sports12040111 (PMC11055131; doi:10.3390/sports12040111)
Supplement: Supplementary file 1 [file sports-12-00111-s001.zip › Supplementary Online Material S4.pdf]

Title: Comparison of the electromyography activity during exercises with stable and unstable surfaces: A systematic review and meta-analysis

Supplementary Online Material S4

Study or Subgroup	Unstable surface Mean	SD	Total	Stable surface Mean	SD	Total	Weight	Std. Mean Difference IV, Random, 95% CI
**1.1.1 Double leg bridge**								
Biscarini; Contemor; Grolla, 2019	6.4	4.6	18	3.6	2.3	18	4.8%	0.75 [0.07, 1.43]
Czaprowski et al., 2013	3.53	2.6	33	2.16	1.6	33	6.3%	0.63 [0.13, 1.12]
Feldwieser et al., 2012	7.39	3.4	20	7.03	3.44	20	5.3%	0.10 [-0.52, 0.72]
Lee; Park; Lee, 2015	0.9	0.7	15	0.8	0.6	15	4.6%	0.15 [-0.57, 0.87]
Luk et al., 2021	4.3	4.2	43	2.2	1.8	43	6.9%	0.64 [0.21, 1.08]
**Subtotal (95% CI)**			**129**			**129**	**28.0%**	**0.51 [0.26, 0.76]**
Heterogeneity: Tau² = 0.00; Chi² = 3.70, df = 4 (P = 0.45); I² = 0%								
Test for overall effect: Z = 3.99 (P < 0.0001)								
**1.1.2 Single-leg bridge**								
Feldwieser et al., 2012	13.02	6.68	20	11.4	5.61	20	5.3%	0.26 [-0.37, 0.88]
Yoon et al., 2017	11.81	7.01	15	10.78	6.37	15	4.6%	0.15 [-0.57, 0.87]
**Subtotal (95% CI)**			**35**			**35**	**9.9%**	**0.21 [-0.26, 0.68]**
Heterogeneity: Tau² = 0.00; Chi² = 0.05, df = 1 (P = 0.82); I² = 0%								
Test for overall effect: Z = 0.88 (P = 0.38)								
**1.1.3 Single-legged hold**								
Ha et al., 2015	7.8	5.9	13	13.9	13.1	13	4.1%	-0.58 [-1.37, 0.21]
Kim et al., 2011	24.27	11.63	19	13	5.77	19	4.7%	1.20 [0.50, 1.90]
**Subtotal (95% CI)**			**32**			**32**	**8.8%**	**0.32 [-1.43, 2.07]**
Heterogeneity: Tau² = 1.45; Chi² = 11.04, df = 1 (P = 0.0009); I² = 91%								
Test for overall effect: Z = 0.36 (P = 0.72)								
**1.1.4 Prone plank**								
Czaprowski et al., 2013	44.7	19.2	33	18.1	9.1	33	5.7%	1.75 [1.18, 2.32]
Lee et al., 2017	39.14	17.69	7	34.93	29.44	7	2.9%	0.16 [-0.89, 1.21]
Snarr; Esco, 2014	65	44	12	36.1	28.1	12	3.9%	0.76 [-0.08, 1.59]
Youdas et al., 2017	54.7	31.6	26	41.2	24.6	26	5.8%	0.47 [-0.08, 1.02]
**Subtotal (95% CI)**			**78**			**78**	**18.2%**	**0.83 [0.10, 1.57]**
Heterogeneity: Tau² = 0.42; Chi² = 12.78, df = 3 (P = 0.005); I² = 77%								
Test for overall effect: Z = 2.21 (P = 0.03)								
**1.1.5 Prone plank with hands**								
Biscarini; Contemor; Grolla, 2019	19.9	11.65	18	15.3	7.89	18	5.0%	0.45 [-0.21, 1.11]
Luk et al., 2021	48.3	24.3	43	26.9	15.1	43	6.7%	1.05 [0.60, 1.50]
**Subtotal (95% CI)**			**61**			**61**	**11.7%**	**0.80 [0.23, 1.38]**
Heterogeneity: Tau² = 0.09; Chi² = 2.12, df = 1 (P = 0.15); I² = 53%								
Test for overall effect: Z = 2.73 (P = 0.006)								
**1.1.6 Side bridge**								
Biscarini; Contemor; Grolla, 2019	25.4	11.5	18	18.1	9.51	18	4.9%	0.68 [0.00, 1.35]
Czaprowski et al., 2013	18.6	6.8	33	16.1	6.7	33	6.4%	0.37 [-0.12, 0.85]
**Subtotal (95% CI)**			**51**			**51**	**11.3%**	**0.47 [0.08, 0.87]**
Heterogeneity: Tau² = 0.00; Chi² = 0.54, df = 1 (P = 0.46); I² = 0%								
Test for overall effect: Z = 2.35 (P = 0.02)								
**1.1.7 Crunch abdominal**								
Sternlicht et al., 2007	1.66	0.99	41	1.27	0.78	41	6.9%	0.43 [-0.00, 0.87]
Vilaça-Alves et al., 2016	610.52	314.62	20	518.06	333.22	20	5.3%	0.28 [-0.34, 0.90]
**Subtotal (95% CI)**			**61**			**61**	**12.1%**	**0.38 [0.02, 0.74]**
Heterogeneity: Tau² = 0.00; Chi² = 0.16, df = 1 (P = 0.69); I² = 0%								
Test for overall effect: Z = 2.09 (P = 0.04)								
**Total (95% CI)**			**447**			**447**	**100.0%**	**0.54 [0.33, 0.75]**
Heterogeneity: Tau² = 0.12; Chi² = 41.21, df = 18 (P = 0.001); I² = 56%								
Test for overall effect: Z = 5.03 (P < 0.00001)								
Test for subgroup differences: Chi² = 3.70, df = 6 (P = 0.72), I² = 0%								

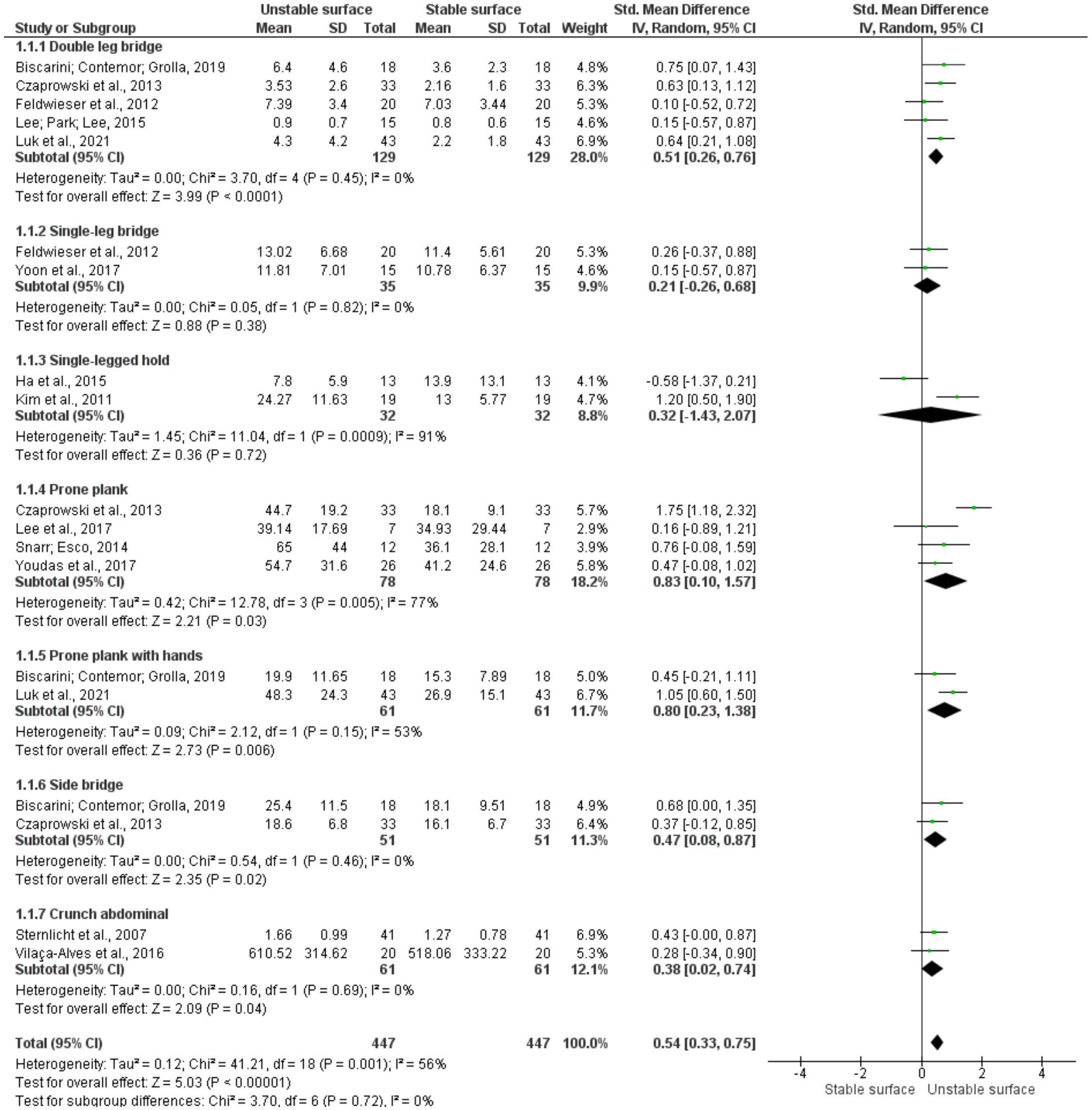

Figure 1. Forest plot of the rectus abdominis muscle EMG activity on an unstable surface versus a stable surface.

Study or Subgroup	Unstable surface Mean	SD	Total	Stable surface Mean	SD	Total	Weight	Std. Mean Difference IV, Random, 95% CI
**5.1.1 Double-leg bridge**								
Biscarini; Contemor; Grolla, 2019	6.2	3.8	18	1.9	1.1	18	4.5%	1.50 [0.75, 2.25]
Czaprowski et al., 2013	6.6	4	33	3.9	2.3	33	7.4%	0.82 [0.31, 1.32]
Feldwieser et al., 2012	8.31	9.81	20	10.18	13.33	20	5.8%	-0.16 [-0.78, 0.46]
Kim; Chung, 2014	6.77	2.31	30	5.71	2.68	30	7.3%	0.42 [-0.09, 0.93]
Lee; Park; Lee, 2015	6.1	6.1	15	4.1	3.4	15	4.7%	0.39 [-0.33, 1.12]
**Subtotal (95% CI)**			**116**			**116**	**29.7%**	**0.58 [0.09, 1.06]**
Heterogeneity: Tau² = 0.21; Chi² = 12.70, df = 4 (P = 0.01); I² = 69%								
Test for overall effect: Z = 2.33 (P = 0.02)								
**5.1.2 Single-leg bridge**								
Feldwieser et al., 2012	17.31	14.93	20	12.81	9.51	20	5.8%	0.35 [-0.27, 0.98]
Kim; Chung, 2014	39.21	13.98	30	33.31	6.74	30	7.2%	0.53 [0.02, 1.05]
**Subtotal (95% CI)**			**50**			**50**	**13.0%**	**0.46 [0.06, 0.86]**
Heterogeneity: Tau² = 0.00; Chi² = 0.19, df = 1 (P = 0.67); I² = 0%								
Test for overall effect: Z = 2.26 (P = 0.02)								
**5.1.3 Single-legged hold**								
Ha et al., 2015	31.1	21.2	13	18	13.1	13	4.1%	0.72 [-0.08, 1.52]
Kim et al., 2011	45.34	19.51	19	25.38	10.92	19	5.0%	1.24 [0.54, 1.94]
**Subtotal (95% CI)**			**32**			**32**	**9.1%**	**1.01 [0.49, 1.54]**
Heterogeneity: Tau² = 0.00; Chi² = 0.91, df = 1 (P = 0.34); I² = 0%								
Test for overall effect: Z = 3.77 (P = 0.0002)								
**5.1.4 Prone Plank**								
Czaprowski et al., 2013	54.7	22.9	33	42.3	19.5	33	7.6%	0.58 [0.08, 1.07]
Lee et al., 2017	23.79	5.59	7	24.81	7.48	7	2.7%	-0.14 [-1.19, 0.90]
Snarr; Esco, 2014	59.1	40.9	12	42	21.6	12	4.0%	0.50 [-0.31, 1.32]
Youdas et al., 2017	88.3	56	26	76.4	63.4	26	6.8%	0.20 [-0.35, 0.74]
**Subtotal (95% CI)**			**78**			**78**	**21.0%**	**0.37 [0.05, 0.69]**
Heterogeneity: Tau² = 0.00; Chi² = 2.09, df = 3 (P = 0.55); I² = 0%								
Test for overall effect: Z = 2.28 (P = 0.02)								
**5.1.5 Crunch Abdominal**								
Sternlicht et al., 2007	0.57	0.3	41	0.46	0.32	41	8.5%	0.35 [-0.09, 0.79]
Vilaça-Alves et al., 2016	552.59	331.24	20	617.36	297.5	20	5.8%	-0.20 [-0.82, 0.42]
**Subtotal (95% CI)**			**61**			**61**	**14.3%**	**0.12 [-0.41, 0.66]**
Heterogeneity: Tau² = 0.08; Chi² = 2.04, df = 1 (P = 0.15); I² = 51%								
Test for overall effect: Z = 0.44 (P = 0.66)								
**5.1.6 Side bridge**								
Biscarini; Contemor; Grolla, 2019	41.6	15.7	18	31.8	10.61	18	5.2%	0.72 [0.04, 1.39]
Czaprowski et al., 2013	45.1	20.8	33	37.6	16.3	33	7.7%	0.40 [-0.09, 0.88]
**Subtotal (95% CI)**			**51**			**51**	**12.9%**	**0.51 [0.11, 0.90]**
Heterogeneity: Tau² = 0.00; Chi² = 0.56, df = 1 (P = 0.45); I² = 0%								
Test for overall effect: Z = 2.51 (P = 0.01)								
**Total (95% CI)**			**388**			**388**	**100.0%**	**0.48 [0.29, 0.67]**
Heterogeneity: Tau² = 0.06; Chi² = 26.12, df = 16 (P = 0.05); I² = 39%								
Test for overall effect: Z = 4.96 (P < 0.00001)								
Test for subgroup differences: Chi² = 6.29, df = 5 (P = 0.28), I² = 20.6%								

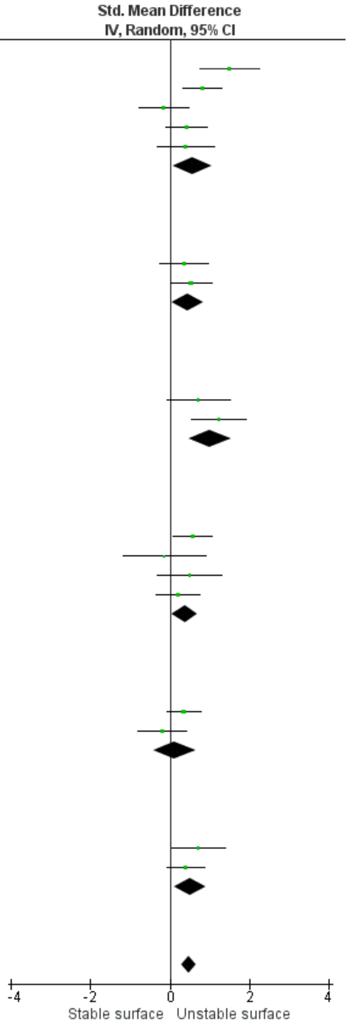

Figure 2. Forest plot of the external oblique muscle EMG activity on an unstable surface versus a stable surface.

Study or Subgroup	Unstable surface Mean	SD	Total	Stable surface Mean	SD	Total	Weight	Std. Mean Difference IV, Random, 95% CI
**4.1.1 Double-leg bridge**								
Biscarini; Contemor; Grolla, 2019	17.2	11	18	4.3	2.7	18	49.5%	1.57 [0.82, 2.33]
Lee; Park; Lee, 2015	4.1	2.4	15	3	1.6	15	50.5%	0.52 [-0.20, 1.25]
**Subtotal (95% CI)**			**33**			**33**	**100.0%**	**1.04 [0.02, 2.07]**
Heterogeneity: Tau² = 0.41; Chi² = 3.82, df = 1 (P = 0.05); I² = 74%								
Test for overall effect: Z = 1.99 (P = 0.05)								
**Total (95% CI)**			**33**			**33**	**100.0%**	**1.04 [0.02, 2.07]**
Heterogeneity: Tau² = 0.41; Chi² = 3.82, df = 1 (P = 0.05); I² = 74%								
Test for overall effect: Z = 1.99 (P = 0.05)								
Test for subgroup differences: Not applicable								

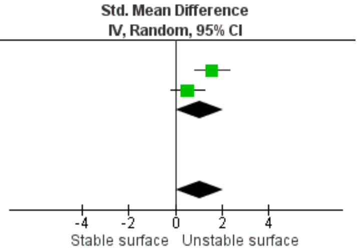

Figure 3. Forest plot of the internal oblique muscle EMG activity on an unstable surface versus a stable surface.

Study or Subgroup	Unstable surface Mean	SD	Total	Stable surface Mean	SD	Total	Weight	Std. Mean Difference IV, Random, 95% CI
**3.1.1 Double-leg bridge**								
Biscarini; Contemor; Grolla, 2019	26.6	8.6	18	27.1	9.3	18	10.6%	-0.05 [-0.71, 0.60]
Kim; Chung, 2014	34.88	8.95	30	32.29	8.06	30	13.3%	0.30 [-0.21, 0.81]
Luk et al., 2021	49.3	16.7	43	34.8	14.1	43	14.7%	0.93 [0.48, 1.38]
**Subtotal (95% CI)**			**91**			**91**	**38.6%**	**0.43 [-0.14, 1.00]**
Heterogeneity: Tau² = 0.18; Chi² = 6.92, df = 2 (P = 0.03); I² = 71%								
Test for overall effect: Z = 1.47 (P = 0.14)								
**3.1.2 Single-leg bridge**								
Kim; Chung, 2014	42.01	7.14	30	39.47	6.19	30	13.3%	0.38 [-0.14, 0.89]
Yoon et al., 2017	25.78	22.07	15	27.66	20.46	15	9.6%	-0.09 [-0.80, 0.63]
**Subtotal (95% CI)**			**45**			**45**	**22.9%**	**0.22 [-0.21, 0.65]**
Heterogeneity: Tau² = 0.01; Chi² = 1.06, df = 1 (P = 0.30); I² = 5%								
Test for overall effect: Z = 0.98 (P = 0.33)								
**3.1.3 Prone plank**								
Lee et al., 2017	23.79	5.59	7	24.81	7.48	7	5.8%	-0.14 [-1.19, 0.90]
Snarr; Esco, 2014	17.5	9.56	12	10	4.1	12	7.7%	0.98 [0.13, 1.84]
**Subtotal (95% CI)**			**19**			**19**	**13.5%**	**0.46 [-0.64, 1.57]**
Heterogeneity: Tau² = 0.40; Chi² = 2.67, df = 1 (P = 0.10); I² = 63%								
Test for overall effect: Z = 0.82 (P = 0.41)								
**3.1.4 Prone plank with hands**								
Biscarini; Contemor; Grolla, 2019	3.1	1.07	18	2.7	0.72	18	10.4%	0.43 [-0.23, 1.09]
Luk et al., 2021	6.2	2.4	43	4.2	1.5	43	14.6%	0.99 [0.54, 1.44]
**Subtotal (95% CI)**			**61**			**61**	**25.1%**	**0.76 [0.22, 1.30]**
Heterogeneity: Tau² = 0.07; Chi² = 1.89, df = 1 (P = 0.17); I² = 47%								
Test for overall effect: Z = 2.78 (P = 0.006)								
**Total (95% CI)**			**216**			**216**	**100.0%**	**0.47 [0.18, 0.76]**
Heterogeneity: Tau² = 0.10; Chi² = 16.94, df = 8 (P = 0.03); I² = 53%								
Test for overall effect: Z = 3.13 (P = 0.002)								
Test for subgroup differences: Chi² = 2.43, df = 3 (P = 0.49), I² = 0%								

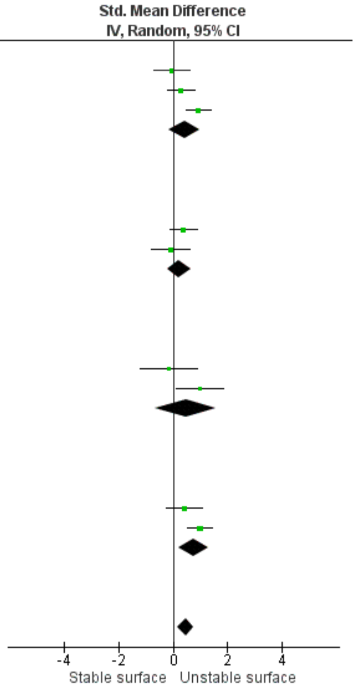

Figure 4. Forest plot of the erector spinae muscle EMG activity on an unstable surface versus a stable surface.

Study or Subgroup	Unstable surface Mean	Unstable surface SD	Unstable surface Total	Stable surface Mean	Stable surface SD	Stable surface Total	Weight	Std. Mean Difference IV, Random, 95% CI
**2.1.1 Double-leg bridge**								
Biscarini; Contemor; Grolla, 2019	26.3	9.1	18	27.2	12.5	18	11.9%	-0.08 [-0.73, 0.57]
Feldwieser et al., 2012	40.71	10.57	20	34.19	12.3	20	12.5%	0.56 [-0.08, 1.19]
Luk et al., 2021	53.6	19.6	43	45.3	18.3	43	20.6%	0.43 [0.01, 0.86]
**Subtotal (95% CI)**			**81**			**81**	**45.0%**	**0.34 [0.01, 0.68]**
Heterogeneity: Tau² = 0.01; Chi² = 2.23, df = 2 (P = 0.33); I² = 10%								
Test for overall effect: Z = 2.01 (P = 0.04)								
**2.1.2 Single-leg bridge**								
Feldwieser et al., 2012	40.53	10.72	20	40.37	11.65	20	12.9%	0.01 [-0.61, 0.63]
Yoon et al., 2017	17.83	16.2	15	18.07	14.06	15	10.4%	-0.02 [-0.73, 0.70]
**Subtotal (95% CI)**			**35**			**35**	**23.3%**	**0.00 [-0.47, 0.47]**
Heterogeneity: Tau² = 0.00; Chi² = 0.00, df = 1 (P = 0.95); I² = 0%								
Test for overall effect: Z = 0.01 (P = 1.00)								
**2.1.3 Prone plank with hands**								
Biscarini; Contemor; Grolla, 2019	4.9	3.47	18	4	2.75	18	11.8%	0.28 [-0.38, 0.94]
Luk et al., 2021	5.5	2.6	43	3.6	1.9	43	19.9%	0.83 [0.39, 1.27]
**Subtotal (95% CI)**			**61**			**61**	**31.7%**	**0.61 [0.09, 1.13]**
Heterogeneity: Tau² = 0.07; Chi² = 1.83, df = 1 (P = 0.18); I² = 45%								
Test for overall effect: Z = 2.29 (P = 0.02)								
**Total (95% CI)**			**177**			**177**	**100.0%**	**0.35 [0.08, 0.61]**
Heterogeneity: Tau² = 0.04; Chi² = 8.81, df = 6 (P = 0.18); I² = 32%								
Test for overall effect: Z = 2.59 (P = 0.009)								
Test for subgroup differences: Chi² = 2.97, df = 2 (P = 0.23), I² = 32.6%								

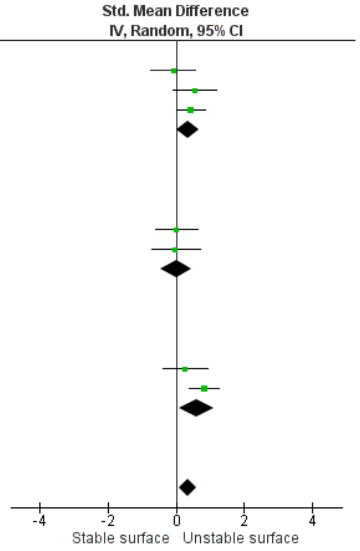

Figure 5. Forest plot of the lumbar multifidus muscle EMG activity on an unstable surface versus a stable surface.

Study or Subgroup	Unstable surface Mean	SD	Total	Stable surface Mean	SD	Total	Weight	Std. Mean Difference IV, Random, 95% CI
**1.1.1 Bench press**								
Aranda et al., 2016	0.3	0.21	19	0.4	0.44	19	4.6%	-0.28 [-0.92, 0.36]
Saeterbakken; Fimland, 2013	0.448	0.19	16	0.555	0.28	16	3.9%	-0.44 [-1.14, 0.27]
Sousa et al., 2022	42.37	20.32	14	41.87	17.34	14	3.6%	0.03 [-0.72, 0.77]
**Subtotal (95% CI)**			**49**			**49**	**12.0%**	**-0.24 [-0.64, 0.16]**
Heterogeneity: Tau² = 0.00; Chi² = 0.81, df = 2 (P = 0.67); I² = 0%								
Test for overall effect: Z = 1.20 (P = 0.23)								
**1.1.2 Chest press (concentric phase)**								
Marshall; Bernadette; Murphy, 2006	453.55	150.61	14	421.25	128.31	14	3.5%	0.22 [-0.52, 0.97]
Uribe et al., 2010	1.74	1.81	16	1.64	1.68	16	4.0%	0.06 [-0.64, 0.75]
**Subtotal (95% CI)**			**30**			**30**	**7.5%**	**0.13 [-0.37, 0.64]**
Heterogeneity: Tau² = 0.00; Chi² = 0.11, df = 1 (P = 0.75); I² = 0%								
Test for overall effect: Z = 0.52 (P = 0.60)								
**1.1.3 Push up (dynamic)**								
Borreani et al., 2015b	30.81	13.24	29	29.25	10.33	29	6.4%	0.13 [-0.39, 0.64]
Calatayud et al., 2014a	29.46	9.15	29	23.58	9.37	29	6.2%	0.63 [0.10, 1.15]
Calatayud et al., 2014b	41.6	15.5	29	29.6	10.12	29	5.9%	0.90 [0.36, 1.45]
Kim et al., 2012	6,968.7	2,755.66	33	5,979.6	2,656.28	33	7.0%	0.36 [-0.13, 0.85]
Palma et al., 2021	55	27.9	12	56.6	34.2	12	3.1%	-0.05 [-0.85, 0.75]
Snarr; Esco, 2013	3.08	1.13	21	2.66	1.05	21	4.9%	0.38 [-0.23, 0.99]
**Subtotal (95% CI)**			**153**			**153**	**33.5%**	**0.43 [0.17, 0.68]**
Heterogeneity: Tau² = 0.02; Chi² = 6.26, df = 5 (P = 0.28); I² = 20%								
Test for overall effect: Z = 3.25 (P = 0.001)								
**1.1.4 Push up (concentric phase)**								
Park; Yoo, 2013	66.09	17.6	14	61.23	17.17	14	3.5%	0.27 [-0.47, 1.02]
Sandhu; Mahajan; Shenoy, 2008	287.1	174.9	35	165.1	117.9	35	6.9%	0.81 [0.32, 1.30]
**Subtotal (95% CI)**			**49**			**49**	**10.5%**	**0.62 [0.11, 1.12]**
Heterogeneity: Tau² = 0.04; Chi² = 1.40, df = 1 (P = 0.24); I² = 29%								
Test for overall effect: Z = 2.39 (P = 0.02)								
**1.1.5 Push up with isometric phases**								
Hwangbo; Kim, 2012	17,239.9	16,930.8	45	11,289.1	7,771.4	45	8.6%	0.45 [0.03, 0.87]
Lehman et al. 2006	29.9	7.6	13	30.4	9.7	13	3.3%	-0.06 [-0.82, 0.71]
**Subtotal (95% CI)**			**58**			**58**	**11.9%**	**0.30 [-0.14, 0.75]**
Heterogeneity: Tau² = 0.03; Chi² = 1.27, df = 1 (P = 0.26); I² = 21%								
Test for overall effect: Z = 1.33 (P = 0.18)								
**1.1.6 Knee push ups**								
De Mey et al., 2014	90.13	66.64	47	82.88	45.82	47	9.0%	0.13 [-0.28, 0.53]
Kim et al., 2012	5,077.1	2,062.29	33	4,278.8	1,615.94	33	6.9%	0.43 [-0.06, 0.91]
**Subtotal (95% CI)**			**80**			**80**	**15.9%**	**0.25 [-0.06, 0.56]**
Heterogeneity: Tau² = 0.00; Chi² = 0.86, df = 1 (P = 0.35); I² = 0%								
Test for overall effect: Z = 1.56 (P = 0.12)								
**1.1.7 One-arm knee press up**								
Herrington; Waterman; Smith, 2015	9.73	12.12	21	6.13	5.4	21	4.9%	0.38 [-0.23, 0.99]
Pontillo et al., 2007	9.6	8.6	15	8.7	7.2	15	3.8%	0.11 [-0.61, 0.83]
**Subtotal (95% CI)**			**36**			**36**	**8.7%**	**0.26 [-0.20, 0.73]**
Heterogeneity: Tau² = 0.00; Chi² = 0.31, df = 1 (P = 0.58); I² = 0%								
Test for overall effect: Z = 1.12 (P = 0.26)								
**Total (95% CI)**			**455**			**455**	**100.0%**	**0.29 [0.14, 0.44]**
Heterogeneity: Tau² = 0.02; Chi² = 22.76, df = 18 (P = 0.20); I² = 21%								
Test for overall effect: Z = 3.77 (P = 0.0002)								
Test for subgroup differences: Chi² = 9.92, df = 6 (P = 0.13), I² = 39.5%								

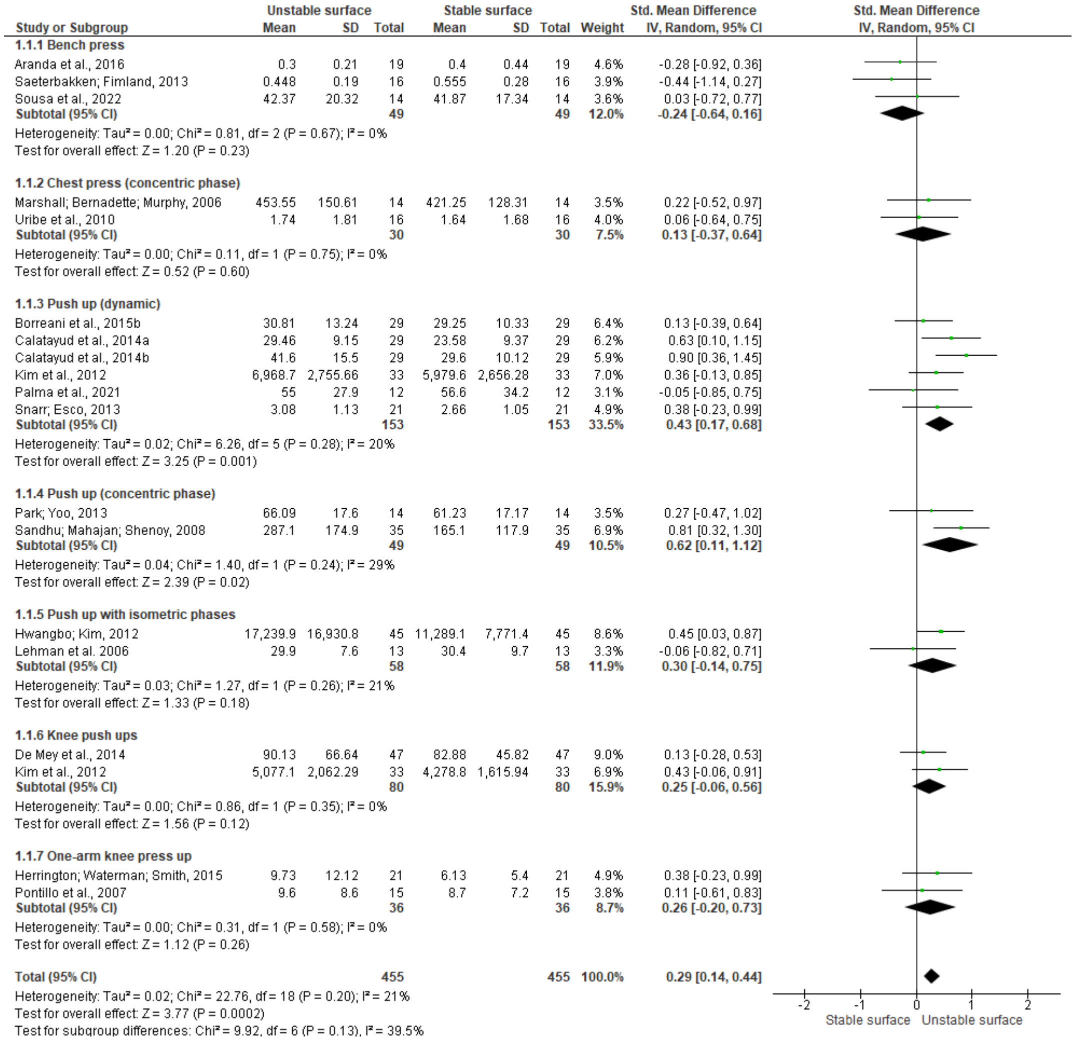

Figure 6. Forest plot of the pectoralis major muscle EMG activity on an unstable surface versus a stable surface.

Study or Subgroup	Unstable surface Mean	SD	Total	Stable surface Mean	SD	Total	Weight	Std. Mean Difference IV, Random, 95% CI
**1.3.1 Bench press**								
Saeterbakken; Fimland, 2013	0.516	0.17	16	0.747	0.23	16	6.5%	-1.11 [-1.87, -0.36]
Sousa et al., 2022	29.03	16.11	14	26.71	13.52	14	6.6%	0.15 [-0.59, 0.89]
**Subtotal (95% CI)**			**30**			**30**	**13.1%**	**-0.48 [-1.72, 0.76]**
Heterogeneity: Tau² = 0.65; Chi² = 5.51, df = 1 (P = 0.02); I² = 82%								
Test for overall effect: Z = 0.76 (P = 0.45)								
**1.3.2 Push up (dynamic)**								
Anderson et al., 2013	63.51	29.08	15	57.48	23.51	15	6.7%	0.22 [-0.50, 0.94]
Borreani et al., 2015b	37.03	9.69	29	17.14	7.05	29	6.9%	2.32 [1.64, 2.99]
Calatayud et al., 2014a	32.95	18.36	29	13.99	8.29	29	7.4%	1.31 [0.74, 1.88]
Calatayud et al., 2014b	24.4	9.04	29	17.14	7.05	29	7.6%	0.88 [0.34, 1.42]
Kim et al., 2012	26,154.4	14,583.14	33	22,780.5	13,105.64	33	7.8%	0.24 [-0.24, 0.72]
Park et al., 2013	67.21	12.18	16	62.05	13.03	16	6.8%	0.40 [-0.30, 1.10]
Snarr; Esco, 2013	5.11	1.97	21	3.91	1.36	21	7.2%	0.70 [0.07, 1.32]
Syed-Abdul et al., 2018	382	333	24	307	230	24	7.4%	0.26 [-0.31, 0.83]
**Subtotal (95% CI)**			**196**			**196**	**57.9%**	**0.79 [0.31, 1.26]**
Heterogeneity: Tau² = 0.37; Chi² = 34.85, df = 7 (P < 0.0001); I² = 80%								
Test for overall effect: Z = 3.25 (P = 0.001)								
**1.3.3 Push up (concentric phase)**								
Park; Yoo, 2013	75.43	11.31	14	72.29	14.07	14	6.6%	0.24 [-0.51, 0.98]
Sandhu; Mahajan; Shenoy, 2008	265.8	113.6	35	179.4	113.4	35	7.8%	0.75 [0.27, 1.24]
**Subtotal (95% CI)**			**49**			**49**	**14.4%**	**0.58 [0.10, 1.05]**
Heterogeneity: Tau² = 0.03; Chi² = 1.29, df = 1 (P = 0.26); I² = 22%								
Test for overall effect: Z = 2.36 (P = 0.02)								
**1.3.4 Push up com fases isométricas**								
Hwangbo; Kim, 2012	6,080.2	3,395	45	5,077.1	2,408.2	45	8.2%	0.34 [-0.08, 0.75]
Lehman et al. 2006	29.9	10.1	13	32.7	17.5	13	6.4%	-0.19 [-0.96, 0.58]
**Subtotal (95% CI)**			**58**			**58**	**14.6%**	**0.18 [-0.30, 0.65]**
Heterogeneity: Tau² = 0.04; Chi² = 1.39, df = 1 (P = 0.24); I² = 28%								
Test for overall effect: Z = 0.73 (P = 0.46)								
**Total (95% CI)**			**333**			**333**	**100.0%**	**0.48 [0.12, 0.84]**
Heterogeneity: Tau² = 0.37; Chi² = 64.03, df = 13 (P < 0.00001); I² = 80%								
Test for overall effect: Z = 2.63 (P = 0.008)								
Test for subgroup differences: Chi² = 5.59, df = 3 (P = 0.13), I² = 46.4%								

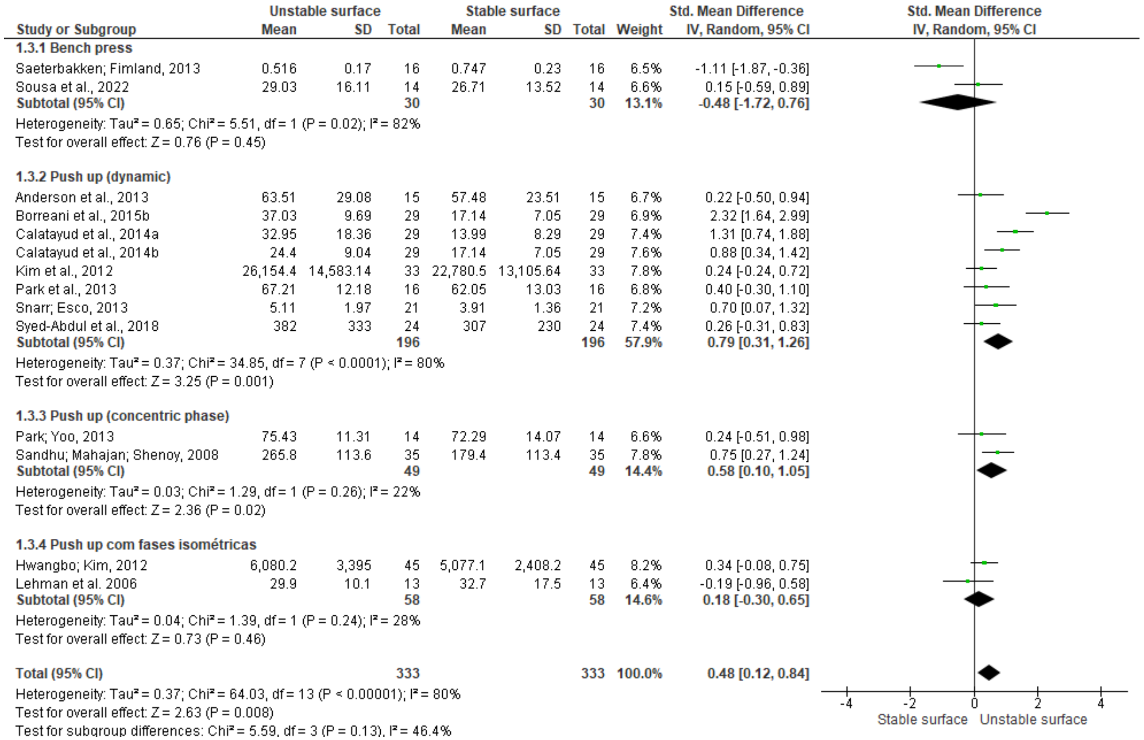

Figure 7. Forest plot of the triceps muscle EMG activity on an unstable surface versus a stable surface.

Study or Subgroup	Unstable surface Mean	SD	Total	Stable surface Mean	SD	Total	Weight	Std. Mean Difference IV, Random, 95% CI
**1.2.1 Bench press**								
Aranda et al., 2016	0.65	0.42	19	0.52	0.13	19	7.6%	0.41 [-0.23, 1.05]
Saeterbakken; Fimland, 2013	0.943	0.29	16	0.945	0.28	16	7.2%	-0.01 [-0.70, 0.69]
Sousa et al., 2022	66.58	36.21	14	68.46	33.68	14	6.9%	-0.05 [-0.79, 0.69]
**Subtotal (95% CI)**			**49**			**49**	**21.7%**	**0.14 [-0.26, 0.54]**
Heterogeneity: Tau² = 0.00; Chi² = 1.10, df = 2 (P = 0.58); I² = 0%								
Test for overall effect: Z = 0.69 (P = 0.49)								
**1.2.2 Chest press (concentric phase)**								
Marshall; Bernadette; Murphy, 2006	449.39	227.86	14	337.24	216.78	14	6.8%	0.49 [-0.26, 1.24]
Uribe et al., 2010	0.85	0.41	16	0.9	0.35	16	7.2%	-0.13 [-0.82, 0.57]
**Subtotal (95% CI)**			**30**			**30**	**14.1%**	**0.16 [-0.44, 0.77]**
Heterogeneity: Tau² = 0.05; Chi² = 1.40, df = 1 (P = 0.24); I² = 28%								
Test for overall effect: Z = 0.53 (P = 0.60)								
**1.2.3 Push up (dynamic)**								
Borreani et al. 2015a	83.74	32.64	30	78.54	24.04	30	8.6%	0.18 [-0.33, 0.69]
Borreani et al., 2015b	19.08	4.9	29	26.22	7.86	29	8.2%	-1.08 [-1.63, -0.52]
Calatayud et al., 2014a	16.42	7.59	29	29.68	13.78	29	8.2%	-1.18 [-1.74, -0.62]
Calatayud et al., 2014b	22.18	7.59	29	26.22	7.86	29	8.5%	-0.52 [-1.04, 0.01]
Snarr; Esco, 2013	5.08	1.55	21	4.01	1.27	21	7.7%	0.74 [0.11, 1.37]
Syed-Abdul et al., 2018	320	240	24	281	171	24	8.1%	0.18 [-0.38, 0.75]
**Subtotal (95% CI)**			**162**			**162**	**49.3%**	**-0.28 [-0.88, 0.31]**
Heterogeneity: Tau² = 0.47; Chi² = 34.39, df = 5 (P < 0.00001); I² = 85%								
Test for overall effect: Z = 0.93 (P = 0.35)								
**1.2.4 One-arm knee press up**								
Herrington; Waterman; Smith, 2015	9.9	7	21	11	7.2	21	7.9%	-0.15 [-0.76, 0.45]
Pontillo et al., 2007	10.95	11.34	15	12.58	9.28	15	7.1%	-0.15 [-0.87, 0.56]
**Subtotal (95% CI)**			**36**			**36**	**14.9%**	**-0.15 [-0.62, 0.31]**
Heterogeneity: Tau² = 0.00; Chi² = 0.00, df = 1 (P = 1.00); I² = 0%								
Test for overall effect: Z = 0.65 (P = 0.52)								
**Total (95% CI)**			**277**			**277**	**100.0%**	**-0.11 [-0.43, 0.21]**
Heterogeneity: Tau² = 0.25; Chi² = 42.11, df = 12 (P < 0.0001); I² = 72%								
Test for overall effect: Z = 0.69 (P = 0.49)								
Test for subgroup differences: Chi² = 2.03, df = 3 (P = 0.57), I² = 0%								

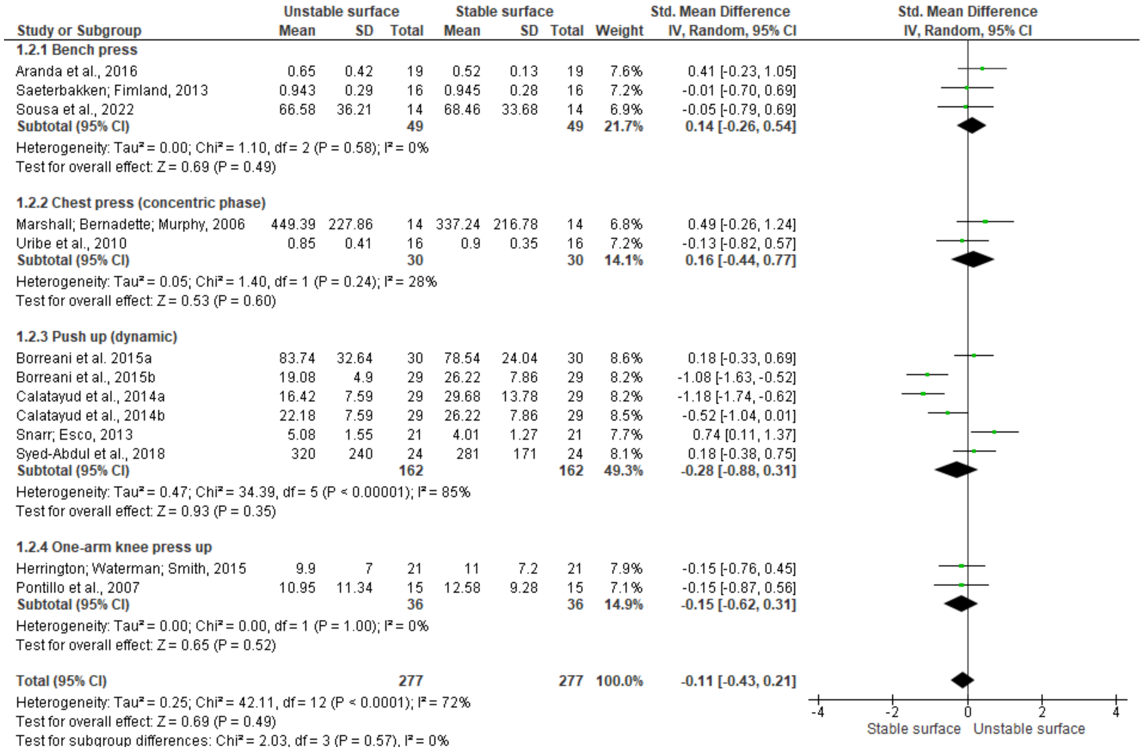

Figure 8. Forest plot of the anterior deltoid muscle EMG activity on an unstable surface versus a stable surface.

Study or Subgroup	Unstable surface Mean	Unstable surface SD	Unstable surface Total	Stable surface Mean	Stable surface SD	Stable surface Total	Weight	Std. Mean Difference IV, Random, 95% CI
**1.1.1 Half Squat (90°) Isometric**								
Jeon et al., 2020	93.54	14.79	40	133.92	21.18	40	33.8%	-2.19 [-2.75, -1.63]
Kim et al., 2021	73.54	53.04	22	68.09	61.48	22	33.6%	0.09 [-0.50, 0.68]
Saeterbakken; Fimland, 2013	0.34	0.09	15	0.41	0.11	15	32.6%	-0.68 [-1.42, 0.06]
**Subtotal (95% CI)**			**77**			**77**	**100.0%**	**-0.93 [-2.36, 0.50]**
Heterogeneity: Tau² = 1.48; Chi² = 31.21, df = 2 (P < 0.00001); I² = 94%								
Test for overall effect: Z = 1.28 (P = 0.20)								
**Total (95% CI)**			**77**			**77**	**100.0%**	**-0.93 [-2.36, 0.50]**
Heterogeneity: Tau² = 1.48; Chi² = 31.21, df = 2 (P < 0.00001); I² = 94%								
Test for overall effect: Z = 1.28 (P = 0.20)								
Test for subgroup differences: Not applicable								

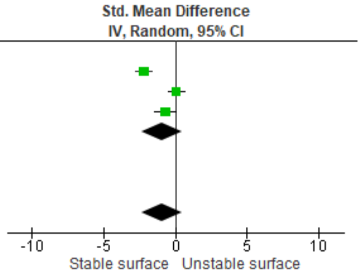

Figure 9. Forest plot of the rectus femoris muscle EMG activity on an unstable surface versus a stable surface.

Study or Subgroup	Unstable surface Mean	SD	Total	Stable surface Mean	SD	Total	Weight	Std. Mean Difference IV, Random, 95% CI
**1.2.1 Squat**								
Andersen et al., 2014	83.2	35.4	15	81.1	22.4	15	6.6%	0.07 [-0.65, 0.78]
Aranda et al.,2016	0.34	0.22	19	0.34	0.21	19	6.8%	0.00 [-0.64, 0.64]
Buscá et al,. 2020	0.74	0.26	14	0.59	0.15	14	6.4%	0.69 [-0.08, 1.45]
Gündoğan; Aydin; Sağlam, 2023	101.52	34.33	14	99.72	33.23	14	6.5%	0.05 [-0.69, 0.79]
Li; Cao; Chen, 2013	32.4	11.6	13	32	11.4	13	6.4%	0.03 [-0.74, 0.80]
**Subtotal (95% CI)**			**75**			**75**	**32.6%**	**0.15 [-0.17, 0.47]**
Heterogeneity: Tau² = 0.00; Chi² = 2.30, df = 4 (P = 0.68); I² = 0%								
Test for overall effect: Z = 0.92 (P = 0.36)								
**1.2.2 Bulgarian squat**								
Aguilera-Castells et al., 2019	76.79	16.99	20	72.34	21.51	20	6.8%	0.23 [-0.40, 0.85]
Andersen et al., 2014	77.8	24.7	15	83.7	25.7	15	6.5%	-0.23 [-0.95, 0.49]
**Subtotal (95% CI)**			**35**			**35**	**13.4%**	**0.03 [-0.44, 0.50]**
Heterogeneity: Tau² = 0.00; Chi² = 0.87, df = 1 (P = 0.35); I² = 0%								
Test for overall effect: Z = 0.13 (P = 0.90)								
**1.2.3 Half Squat (90°) Isometric**								
Jeon et al., 2020	160.01	25.3	40	88.67	14.02	40	6.6%	3.45 [2.75, 4.16]
Kim et al., 2021	53.8	20.99	22	54.67	27.24	22	6.9%	-0.04 [-0.63, 0.56]
Marshall; Murphy, 2006	23.62	10.43	12	22.68	11.15	12	6.3%	0.08 [-0.72, 0.88]
Saeterbakken; Fimland, 2013	0.44	0.12	15	0.46	0.13	15	6.6%	-0.16 [-0.87, 0.56]
**Subtotal (95% CI)**			**89**			**89**	**26.4%**	**0.84 [-0.87, 2.55]**
Heterogeneity: Tau² = 2.92; Chi² = 72.42, df = 3 (P < 0.00001); I² = 96%								
Test for overall effect: Z = 0.96 (P = 0.34)								
**1.2.4 Squat (60°) Isometric**								
Hyong; Kang, 2013	50.92	20.87	14	48.64	14.8	14	6.5%	0.12 [-0.62, 0.86]
Kang et al., 2017	159.96	25.26	24	165.15	24.7	24	7.0%	-0.20 [-0.77, 0.36]
Marín; Hazell, 2014	0.0355	0.0126	28	0.0399	0.0158	28	7.1%	-0.30 [-0.83, 0.22]
Park et al., 2015	53.49	23.15	25	45	21.83	25	7.0%	0.37 [-0.19, 0.93]
**Subtotal (95% CI)**			**91**			**91**	**27.6%**	**-0.02 [-0.34, 0.29]**
Heterogeneity: Tau² = 0.02; Chi² = 3.54, df = 3 (P = 0.32); I² = 15%								
Test for overall effect: Z = 0.15 (P = 0.88)								
**Total (95% CI)**			**290**			**290**	**100.0%**	**0.27 [-0.17, 0.72]**
Heterogeneity: Tau² = 0.65; Chi² = 93.56, df = 14 (P < 0.00001); I² = 85%								
Test for overall effect: Z = 1.20 (P = 0.23)								
Test for subgroup differences: Chi² = 1.37, df = 3 (P = 0.71), I² = 0%								

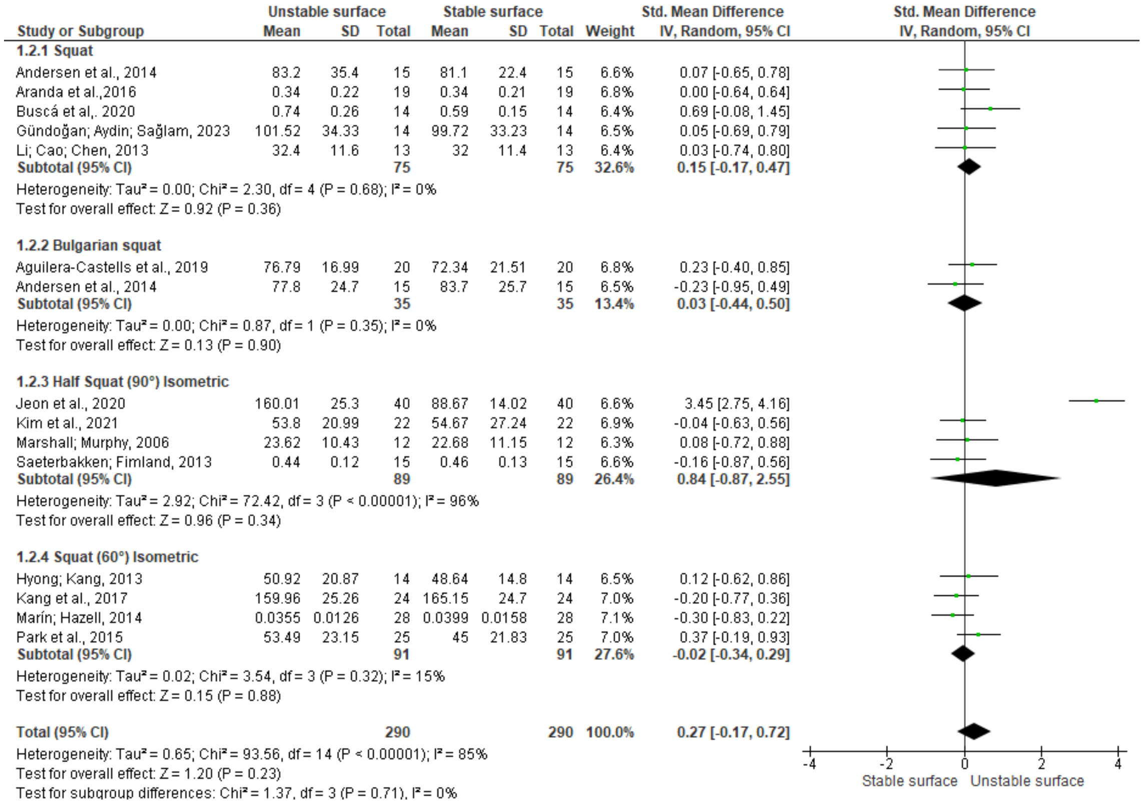

Figure 10. Forest plot of the vastus lateralis muscle EMG activity on an unstable surface versus a stable surface.

Study or Subgroup	Unstable surface Mean	SD	Total	Stable surface Mean	SD	Total	Weight	Std. Mean Difference IV, Random, 95% CI
**1.3.1 Squat**								
Buscá et al,. 2020	0.73	0.22	14	0.63	0.22	14	7.6%	0.44 [-0.31, 1.19]
Gündoğan; Aydin; Sağlam, 2023	89.16	16.74	14	84.57	19.04	14	7.6%	0.25 [-0.50, 0.99]
Li; Cao; Chen, 2013	33.7	6.5	13	32.2	8.7	13	7.5%	0.19 [-0.58, 0.96]
**Subtotal (95% CI)**			**41**			**41**	**22.7%**	**0.29 [-0.14, 0.73]**
Heterogeneity: Tau² = 0.00; Chi² = 0.23, df = 2 (P = 0.89); I² = 0%								
Test for overall effect: Z = 1.32 (P = 0.19)								
**1.3.2 Squat (60°) Isometric**								
Hyong; Kang, 2013	69.5	17.65	14	44.28	18.19	14	7.3%	1.37 [0.53, 2.20]
Kang et al., 2017	150.01	31.44	24	176.63	36.38	24	8.0%	-0.77 [-1.36, -0.18]
Marín; Hazell, 2014	0.0376	0.0125	28	0.044	0.0167	28	8.1%	-0.43 [-0.96, 0.10]
Park et al., 2015	41.21	17.92	25	35.75	17.72	25	8.1%	0.30 [-0.26, 0.86]
**Subtotal (95% CI)**			**91**			**91**	**31.6%**	**0.07 [-0.72, 0.87]**
Heterogeneity: Tau² = 0.56; Chi² = 20.25, df = 3 (P = 0.0002); I² = 85%								
Test for overall effect: Z = 0.18 (P = 0.85)								
**1.3.3 Half Squat (90°) Isometric**								
Han et al., 2017	48	13	15	48.5	12.1	15	7.7%	-0.04 [-0.75, 0.68]
Jeon et al., 2020	168.22	26.6	40	104.14	16.47	40	7.9%	2.87 [2.24, 3.50]
Kim et al., 2021	45.75	18.41	22	51.52	25.55	22	8.0%	-0.25 [-0.85, 0.34]
Saeterbakken; Fimland, 2013	0.56	0.26	15	0.67	0.51	15	7.7%	-0.26 [-0.98, 0.45]
**Subtotal (95% CI)**			**92**			**92**	**31.2%**	**0.58 [-0.97, 2.13]**
Heterogeneity: Tau² = 2.39; Chi² = 65.96, df = 3 (P < 0.00001); I² = 95%								
Test for overall effect: Z = 0.73 (P = 0.46)								
**1.3.4 Deep Squat (100°) Isometric**								
Han et al., 2017	47.3	14.7	15	48	13.6	15	7.7%	-0.05 [-0.76, 0.67]
Mcbride; Cormie; Deane, 2006	119.56	54.53	9	182.35	63.28	9	6.8%	-1.01 [-2.01, -0.02]
**Subtotal (95% CI)**			**24**			**24**	**14.5%**	**-0.47 [-1.40, 0.47]**
Heterogeneity: Tau² = 0.27; Chi² = 2.37, df = 1 (P = 0.12); I² = 58%								
Test for overall effect: Z = 0.97 (P = 0.33)								
**Total (95% CI)**			**248**			**248**	**100.0%**	**0.20 [-0.35, 0.76]**
Heterogeneity: Tau² = 0.90; Chi² = 102.70, df = 12 (P < 0.00001); I² = 88%								
Test for overall effect: Z = 0.72 (P = 0.47)								
Test for subgroup differences: Chi² = 2.40, df = 3 (P = 0.49), I² = 0%								

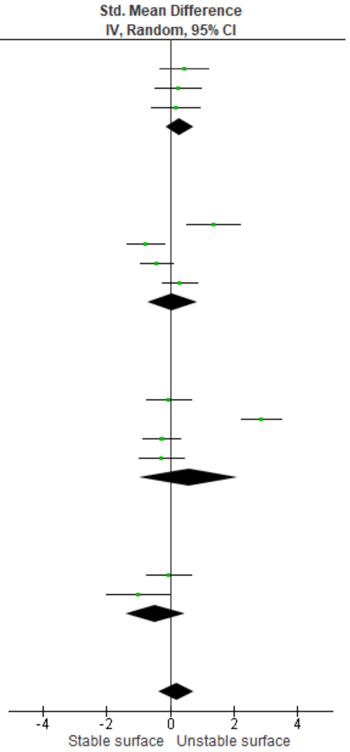

Figure 11. Forest plot of the vastus medialis muscle EMG activity on an unstable surface versus a stable surface.

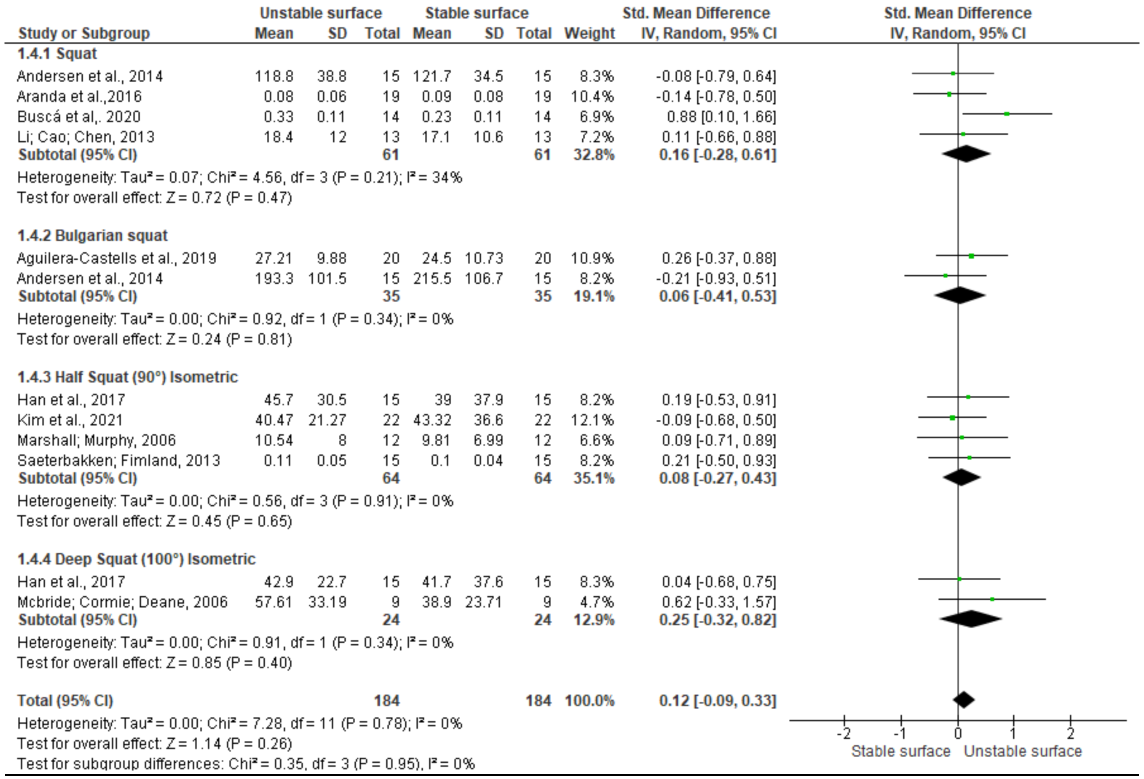

Figure 12. Forest plot of the biceps femoris muscle EMG activity on an unstable surface versus a stable surface.

Table 1. Intensity of exercises included in the meta-analysis.

EXERCISE	STUDY	INTENSITY
BENCH PRESS	ARANDA et al., 2016	15 RMs
BENCH PRESS	SOUSA et al., 2022	50% of 1 RM
BENCH PRESS	SAETERBAKKEN; FIMLAND, 2013	50% of 6 RM
CHEST PRESS	MARSHALL; MURPHY, 2006	60% of 1 RM.
CHEST PRESS	URIBE et al., 2010	80% of 1 RM.
SQUAT	ANDERSEN et al., 2014	6 RMs
SQUAT	ARANDA et al., 2016	15 RMs
SQUAT	BUSCÀ et al., 2020	80% of 1 RM
SQUAT	LI; CAO; CHEN, 2013	60% of 1 RM
SQUAT	GÜNDOĞAN; AYDIN; SAĞLAM, 2023	Body weight
