# Supplementary material for: Comparison of the Electromyography Activity during Exercises with Stable and Unstable Surfaces: A Systematic Review and Meta-Analysis
Source: Sports (Basel). 2024 Apr 18;12(4):111. doi: 10.3390/sports12040111 (PMC11055131; doi:10.3390/sports12040111)
Supplement: Supplementary file 1 [file sports-12-00111-s001.zip › Supplementary Online Material S5.pdf]

Title: Comparison of the electromyography activity during exercises with stable and unstable surfaces: A systematic review and meta-analysis

Supplementary Online Material S5 - Sensitivity Analysis

	Unstable surface			Stable surface				Std. Mean Difference
**Study or Subgroup**	**Mean**	**SD**	**Total**	**Mean**	**SD**	**Total**	**Weight**	**IV, Random, 95% CI**
**1.1.1 Double leg bridge**								
Biscarini; Contemor; Grolla, 2019	6.4	4.6	18	3.6	2.3	18	4.6%	0.75 [0.07, 1.43]
Czaprowski et al., 2013	3.53	2.6	33	2.16	1.6	33	8.7%	0.63 [0.13, 1.12]
Feldwieser et al., 2012	7.39	3.4	20	7.03	3.44	20	5.6%	0.10 [-0.52, 0.72]
Lee; Park; Lee, 2015	0.9	0.7	15	0.8	0.6	15	4.2%	0.15 [-0.57, 0.87]
Luk et al., 2021	4.3	4.2	43	2.2	1.8	43	11.4%	0.64 [0.21, 1.08]
**Subtotal (95% CI)**			**129**			**129**	**34.5%**	**0.51 [0.26, 0.76]**
Heterogeneity: Tau² = 0.00; Chi² = 3.70, df = 4 (P = 0.45); I² = 0%								
Test for overall effect: Z = 3.99 (P < 0.0001)								
**1.1.2 Single-leg bridge**								
Feldwieser et al., 2012	13.02	6.68	20	11.4	5.61	20	5.5%	0.26 [-0.37, 0.88]
Yoon et al., 2017	11.81	7.01	15	10.78	6.37	15	4.2%	0.15 [-0.57, 0.87]
**Subtotal (95% CI)**			**35**			**35**	**9.7%**	**0.21 [-0.26, 0.68]**
Heterogeneity: Tau² = 0.00; Chi² = 0.05, df = 1 (P = 0.82); I² = 0%								
Test for overall effect: Z = 0.88 (P = 0.38)								
**1.1.3 Single-legged hold**								
Ha et al., 2015	7.8	5.9	13	13.9	13.1	13		Not estimable
Kim et al., 2011	24.27	11.63	19	13	5.77	19		Not estimable
**Subtotal (95% CI)**			**0**			**0**		**Not estimable**
Heterogeneity: Not applicable								
Test for overall effect: Not applicable								
**1.1.4 Prone plank**								
Czaprowski et al., 2013	44.7	19.2	33	18.1	9.1	33		Not estimable
Lee et al., 2017	39.14	17.69	7	34.93	29.44	7		Not estimable
Snarr; Esco, 2014	65	44	12	36.1	28.1	12	3.1%	0.76 [-0.08, 1.59]
Youdas et al., 2017	54.7	31.6	26	41.2	24.6	26	7.0%	0.47 [-0.08, 1.02]
**Subtotal (95% CI)**			**38**			**38**	**10.1%**	**0.56 [0.10, 1.02]**
Heterogeneity: Tau² = 0.00; Chi² = 0.32, df = 1 (P = 0.57); I² = 0%								
Test for overall effect: Z = 2.37 (P = 0.02)								
**1.1.5 Prone plank with hands**								
Biscarini; Contemor; Grolla, 2019	19.9	11.65	18	15.3	7.89	18	4.9%	0.45 [-0.21, 1.11]
Luk et al., 2021	48.3	24.3	43	26.9	15.1	43	10.5%	1.05 [0.60, 1.50]
**Subtotal (95% CI)**			**61**			**61**	**15.3%**	**0.80 [0.23, 1.38]**
Heterogeneity: Tau² = 0.09; Chi² = 2.12, df = 1 (P = 0.15); I² = 53%								
Test for overall effect: Z = 2.73 (P = 0.006)								
**1.1.6 Side bridge**								
Biscarini; Contemor; Grolla, 2019	25.4	11.5	18	18.1	9.51	18	4.7%	0.68 [0.00, 1.35]
Czaprowski et al., 2013	18.6	6.8	33	16.1	6.7	33	9.0%	0.37 [-0.12, 0.85]
**Subtotal (95% CI)**			**51**			**51**	**13.7%**	**0.47 [0.08, 0.87]**
Heterogeneity: Tau² = 0.00; Chi² = 0.54, df = 1 (P = 0.46); I² = 0%								
Test for overall effect: Z = 2.35 (P = 0.02)								
**1.1.7 Crunch abdominal**								
Sternlicht et al., 2007	1.66	0.99	41	1.27	0.78	41	11.1%	0.43 [-0.00, 0.87]
Vilaça-Alves et al., 2016	610.52	314.62	20	518.06	333.22	20	5.5%	0.28 [-0.34, 0.90]
**Subtotal (95% CI)**			**61**			**61**	**16.7%**	**0.38 [0.02, 0.74]**
Heterogeneity: Tau² = 0.00; Chi² = 0.16, df = 1 (P = 0.69); I² = 0%								
Test for overall effect: Z = 2.09 (P = 0.04)								
**Total (95% CI)**			**375**			**375**	**100.0%**	**0.51 [0.37, 0.66]**
Heterogeneity: Tau² = 0.00; Chi² = 12.35, df = 14 (P = 0.58); I² = 0%								
Test for overall effect: Z = 6.86 (P < 0.00001)								
Test for subgroup differences: Chi² = 2.89, df = 5 (P = 0.72), I² = 0%								

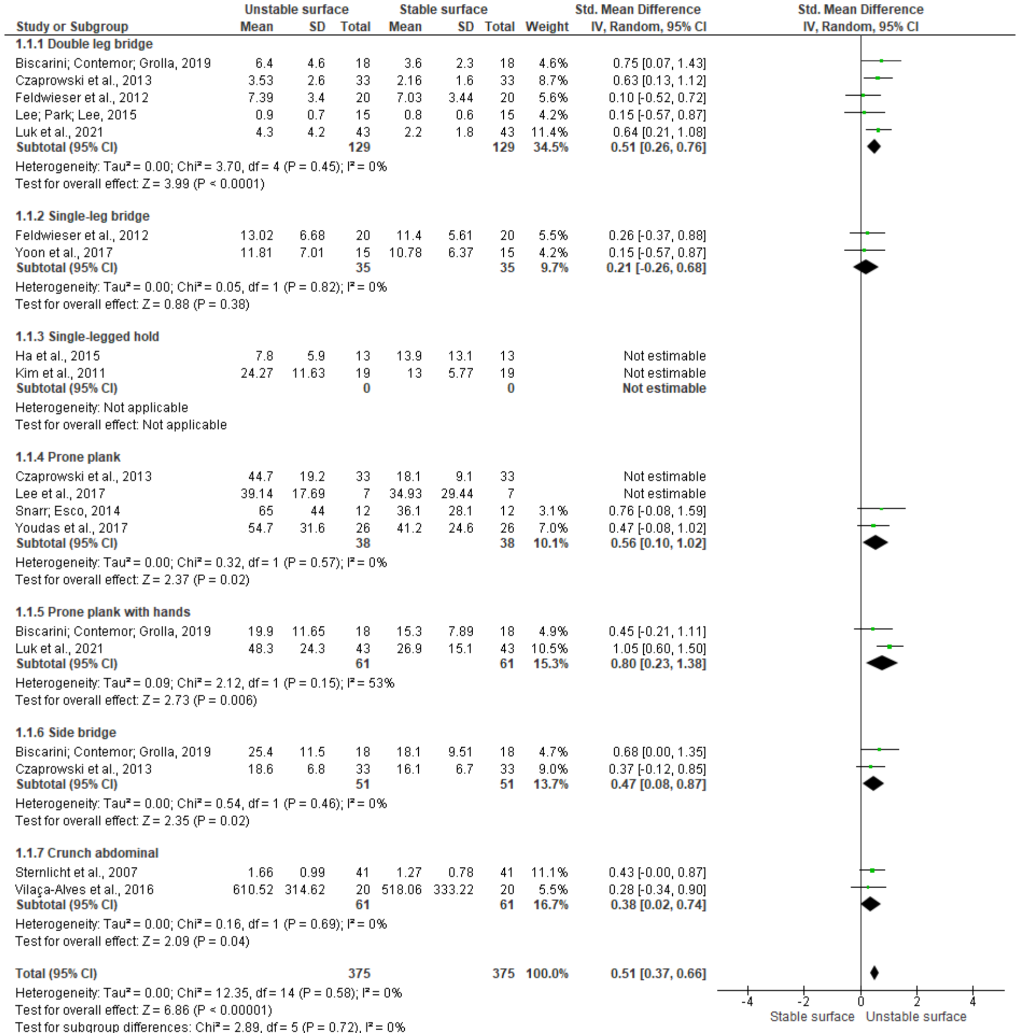

Figure 1. Forest plot of the rectus abdominis muscle EMG activity on an unstable surface versus a stable surface.

Study or Subgroup	Unstable surface Mean	Unstable surface SD	Unstable surface Total	Stable surface Mean	Stable surface SD	Stable surface Total	Weight	Std. Mean Difference IV, Random, 95% CI
**5.1.1 Double-leg bridge**								
Biscarini; Contemor; Grolla, 2019	6.2	3.8	18	1.9	1.1	18		Not estimable
Czaprowski et al., 2013	6.6	4	33	3.9	2.3	33	8.4%	0.82 [0.31, 1.32]
Feldwieser et al., 2012	8.31	9.81	20	10.18	13.33	20	6.0%	-0.16 [-0.78, 0.46]
Kim; Chung, 2014	6.77	2.31	30	5.71	2.68	30	8.2%	0.42 [-0.09, 0.93]
Lee; Park; Lee, 2015	6.1	6.1	15	4.1	3.4	15	4.6%	0.39 [-0.33, 1.12]
**Subtotal (95% CI)**			**98**			**98**	**27.1%**	**0.39 [-0.01, 0.80]**
Heterogeneity: Tau² = 0.08; Chi² = 5.72, df = 3 (P = 0.13); I² = 48%								
Test for overall effect: Z = 1.93 (P = 0.05)								
**5.1.2 Single-leg bridge**								
Feldwieser et al., 2012	17.31	14.93	20	12.81	9.51	20	5.9%	0.35 [-0.27, 0.98]
Kim; Chung, 2014	39.21	13.98	30	33.31	6.74	30	8.1%	0.53 [0.02, 1.05]
**Subtotal (95% CI)**			**50**			**50**	**14.0%**	**0.46 [0.06, 0.86]**
Heterogeneity: Tau² = 0.00; Chi² = 0.19, df = 1 (P = 0.67); I² = 0%								
Test for overall effect: Z = 2.26 (P = 0.02)								
**5.1.3 Single-legged hold**								
Ha et al., 2015	31.1	21.2	13	18	13.1	13	3.9%	0.72 [-0.08, 1.52]
Kim et al., 2011	45.34	19.51	19	25.38	10.92	19	4.9%	1.24 [0.54, 1.94]
**Subtotal (95% CI)**			**32**			**32**	**8.8%**	**1.01 [0.49, 1.54]**
Heterogeneity: Tau² = 0.00; Chi² = 0.91, df = 1 (P = 0.34); I² = 0%								
Test for overall effect: Z = 3.77 (P = 0.0002)								
**5.1.4 Prone Plank**								
Czaprowski et al., 2013	54.7	22.9	33	42.3	19.5	33	8.6%	0.58 [0.08, 1.07]
Lee et al., 2017	23.79	5.59	7	24.81	7.48	7		Not estimable
Snarr; Esco, 2014	59.1	40.9	12	42	21.6	12	3.7%	0.50 [-0.31, 1.32]
Youdas et al., 2017	88.3	56	26	76.4	63.4	26	7.4%	0.20 [-0.35, 0.74]
**Subtotal (95% CI)**			**71**			**71**	**19.8%**	**0.42 [0.09, 0.76]**
Heterogeneity: Tau² = 0.00; Chi² = 1.08, df = 2 (P = 0.58); I² = 0%								
Test for overall effect: Z = 2.48 (P = 0.01)								
**5.1.5 Crunch Abdominal**								
Sternlicht et al., 2007	0.57	0.3	41	0.46	0.32	41	10.3%	0.35 [-0.09, 0.79]
Vilaça-Alves et al., 2016	552.59	331.24	20	617.36	297.5	20	6.0%	-0.20 [-0.82, 0.42]
**Subtotal (95% CI)**			**61**			**61**	**16.3%**	**0.12 [-0.41, 0.66]**
Heterogeneity: Tau² = 0.08; Chi² = 2.04, df = 1 (P = 0.15); I² = 51%								
Test for overall effect: Z = 0.44 (P = 0.66)								
**5.1.6 Side bridge**								
Biscarini; Contemor; Grolla, 2019	41.6	15.7	18	31.8	10.61	18	5.2%	0.72 [0.04, 1.39]
Czaprowski et al., 2013	45.1	20.8	33	37.6	16.3	33	8.8%	0.40 [-0.09, 0.88]
**Subtotal (95% CI)**			**51**			**51**	**14.0%**	**0.51 [0.11, 0.90]**
Heterogeneity: Tau² = 0.00; Chi² = 0.56, df = 1 (P = 0.45); I² = 0%								
Test for overall effect: Z = 2.51 (P = 0.01)								
**Total (95% CI)**			**363**			**363**	**100.0%**	**0.44 [0.28, 0.61]**
Heterogeneity: Tau² = 0.02; Chi² = 17.37, df = 14 (P = 0.24); I² = 19%								
Test for overall effect: Z = 5.20 (P < 0.00001)								
Test for subgroup differences: Chi² = 5.95, df = 5 (P = 0.31), I² = 16.0%								

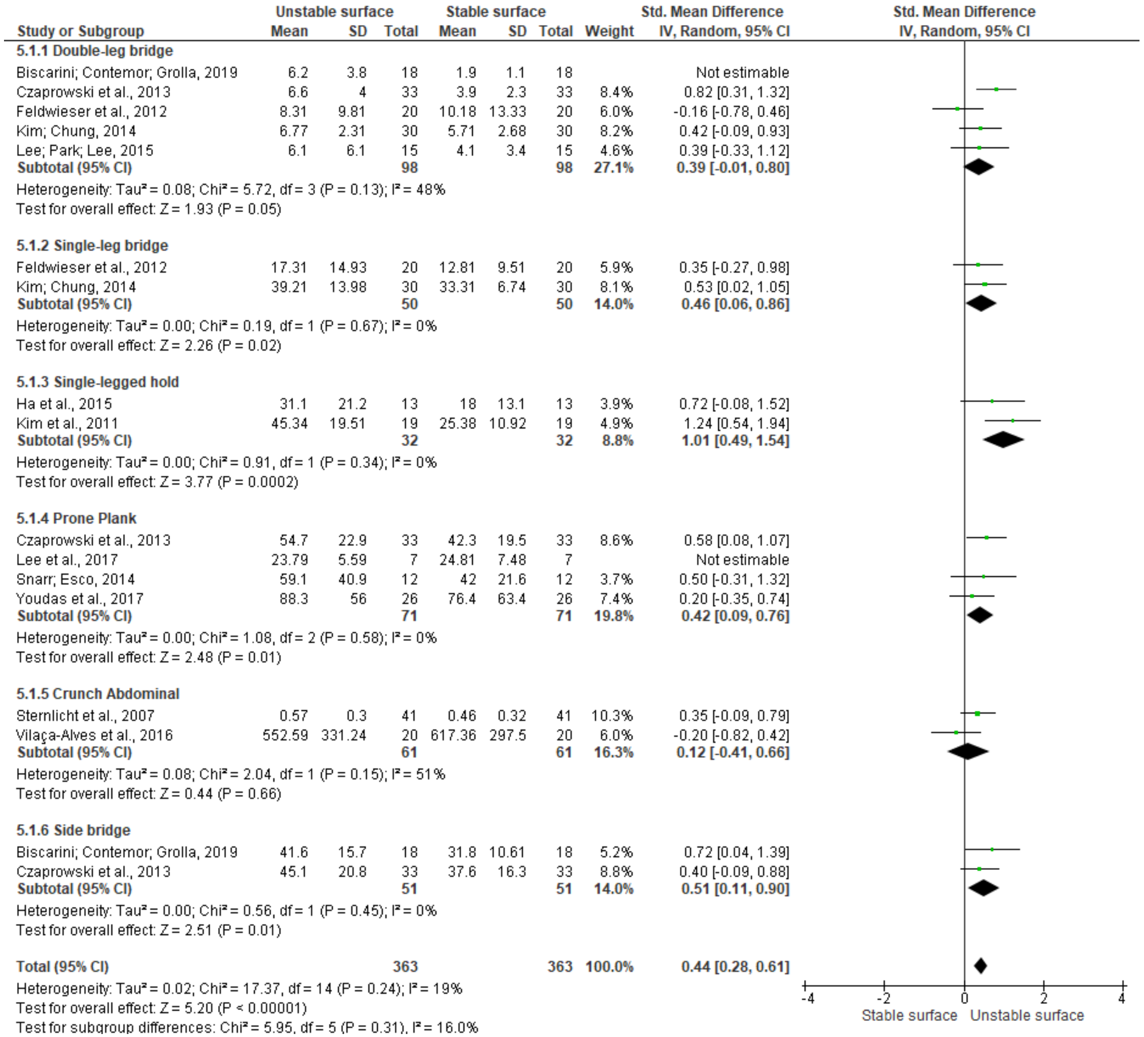

Figure 2. Forest plot of the external oblique muscle EMG activity on an unstable surface versus a stable surface.

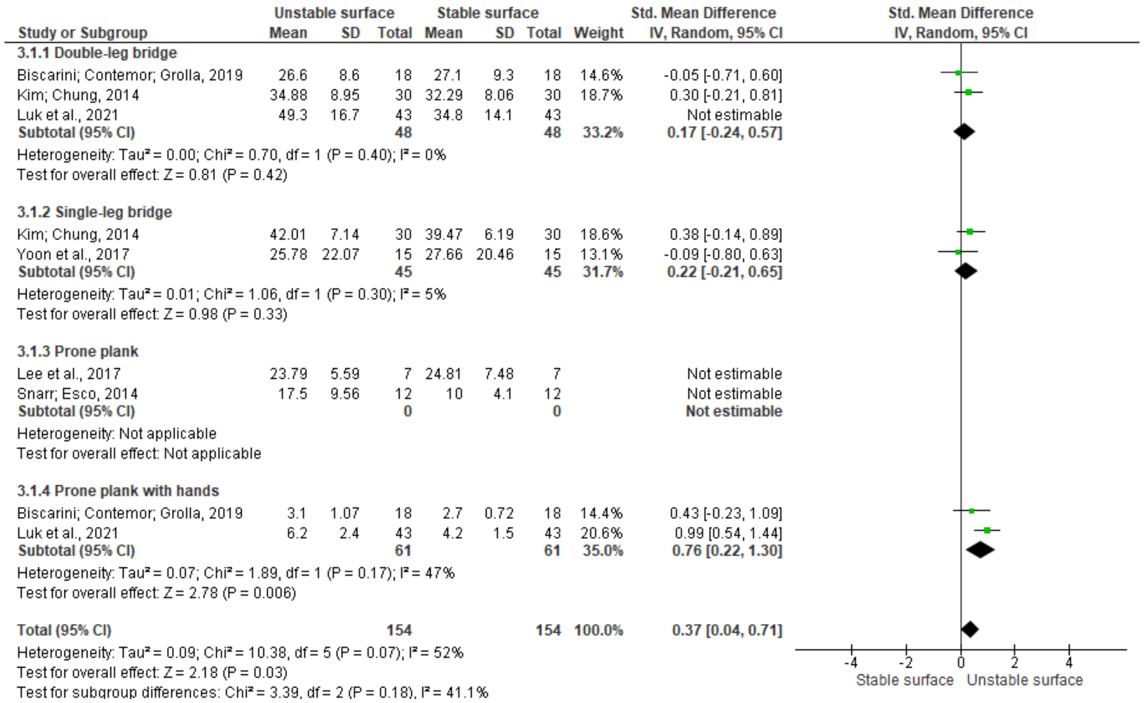

Figure 3. Forest plot of the erector spinae muscle EMG activity on an unstable surface versus a stable surface.

Study or Subgroup	Unstable surface Mean	SD	Total	Stable surface Mean	SD	Total	Weight	Std. Mean Difference IV, Random, 95% CI
**1.1.1 Bench press**								
Aranda et al., 2016	0.3	0.21	19	0.4	0.44	19	6.0%	-0.28 [-0.92, 0.36]
Saeterbakken; Fimland, 2013	0.448	0.19	16	0.555	0.28	16	5.3%	-0.44 [-1.14, 0.27]
Sousa et al., 2022	42.37	20.32	14	41.87	17.34	14	4.9%	0.03 [-0.72, 0.77]
**Subtotal (95% CI)**			**49**			**49**	**16.1%**	**-0.24 [-0.64, 0.16]**
Heterogeneity: Tau² = 0.00; Chi² = 0.81, df = 2 (P = 0.67); I² = 0%								
Test for overall effect: Z = 1.20 (P = 0.23)								
**1.1.2 Chest press (concentric phase)**								
Marshall; Bernadette; Murphy, 2006	453.55	150.61	14	421.25	128.31	14	4.8%	0.22 [-0.52, 0.97]
Uribe et al., 2010	1.74	1.81	16	1.64	1.68	16	5.4%	0.06 [-0.64, 0.75]
**Subtotal (95% CI)**			**30**			**30**	**10.2%**	**0.13 [-0.37, 0.64]**
Heterogeneity: Tau² = 0.00; Chi² = 0.11, df = 1 (P = 0.75); I² = 0%								
Test for overall effect: Z = 0.52 (P = 0.60)								
**1.1.3 Push up (dynamic)**								
Borreani et al., 2015b	30.81	13.24	29	29.25	10.33	29	8.0%	0.13 [-0.39, 0.64]
Calatayud et al., 2014a	29.46	9.15	29	23.58	9.37	29	7.8%	0.63 [0.10, 1.15]
Calatayud et al., 2014b	41.6	15.5	29	29.6	10.12	29	7.5%	0.90 [0.36, 1.45]
Kim et al., 2012	6,968.7	2,755.66	33	5,979.6	2,656.28	33		Not estimable
Palma et al., 2021	55	27.9	12	56.6	34.2	12	4.3%	-0.05 [-0.85, 0.75]
Snarr; Esco, 2013	3.08	1.13	21	2.66	1.05	21	6.4%	0.38 [-0.23, 0.99]
**Subtotal (95% CI)**			**120**			**120**	**34.1%**	**0.44 [0.11, 0.76]**
Heterogeneity: Tau² = 0.05; Chi² = 6.16, df = 4 (P = 0.19); I² = 35%								
Test for overall effect: Z = 2.64 (P = 0.008)								
**1.1.4 Push up (concentric phase)**								
Park; Yoo, 2013	66.09	17.6	14	61.23	17.17	14	4.8%	0.27 [-0.47, 1.02]
Sandhu; Mahajan; Shenoy, 2008	287.1	174.9	35	165.1	117.9	35	8.6%	0.81 [0.32, 1.30]
**Subtotal (95% CI)**			**49**			**49**	**13.4%**	**0.62 [0.11, 1.12]**
Heterogeneity: Tau² = 0.04; Chi² = 1.40, df = 1 (P = 0.24); I² = 29%								
Test for overall effect: Z = 2.39 (P = 0.02)								
**1.1.5 Push up with isometric phases**								
Hwangbo; Kim, 2012	17,239.9	16,930.8	45	11,289.1	7,771.4	45	10.1%	0.45 [0.03, 0.87]
Lehman et al. 2006	29.9	7.6	13	30.4	9.7	13	4.6%	-0.06 [-0.82, 0.71]
**Subtotal (95% CI)**			**58**			**58**	**14.7%**	**0.30 [-0.14, 0.75]**
Heterogeneity: Tau² = 0.03; Chi² = 1.27, df = 1 (P = 0.26); I² = 21%								
Test for overall effect: Z = 1.33 (P = 0.18)								
**1.1.6 Knee push ups**								
De Mey et al., 2014	90.13	66.64	47	82.88	45.82	47		Not estimable
Kim et al., 2012	5,077.1	2,062.29	33	4,278.8	1,615.94	33		Not estimable
**Subtotal (95% CI)**			**0**			**0**		**Not estimable**
Heterogeneity: Not applicable								
Test for overall effect: Not applicable								
**1.1.7 One-arm knee press up**								
Herrington; Waterman; Smith, 2015	9.73	12.12	21	6.13	5.4	21	6.4%	0.38 [-0.23, 0.99]
Pontillo et al., 2007	9.6	8.6	15	8.7	7.2	15	5.1%	0.11 [-0.61, 0.83]
**Subtotal (95% CI)**			**36**			**36**	**11.5%**	**0.26 [-0.20, 0.73]**
Heterogeneity: Tau² = 0.00; Chi² = 0.31, df = 1 (P = 0.58); I² = 0%								
Test for overall effect: Z = 1.12 (P = 0.26)								
**Total (95% CI)**			**342**			**342**	**100.0%**	**0.28 [0.09, 0.47]**
Heterogeneity: Tau² = 0.04; Chi² = 21.72, df = 15 (P = 0.12); I² = 31%								
Test for overall effect: Z = 2.94 (P = 0.003)								
Test for subgroup differences: Chi² = 9.46, df = 5 (P = 0.09), I² = 47.1%								

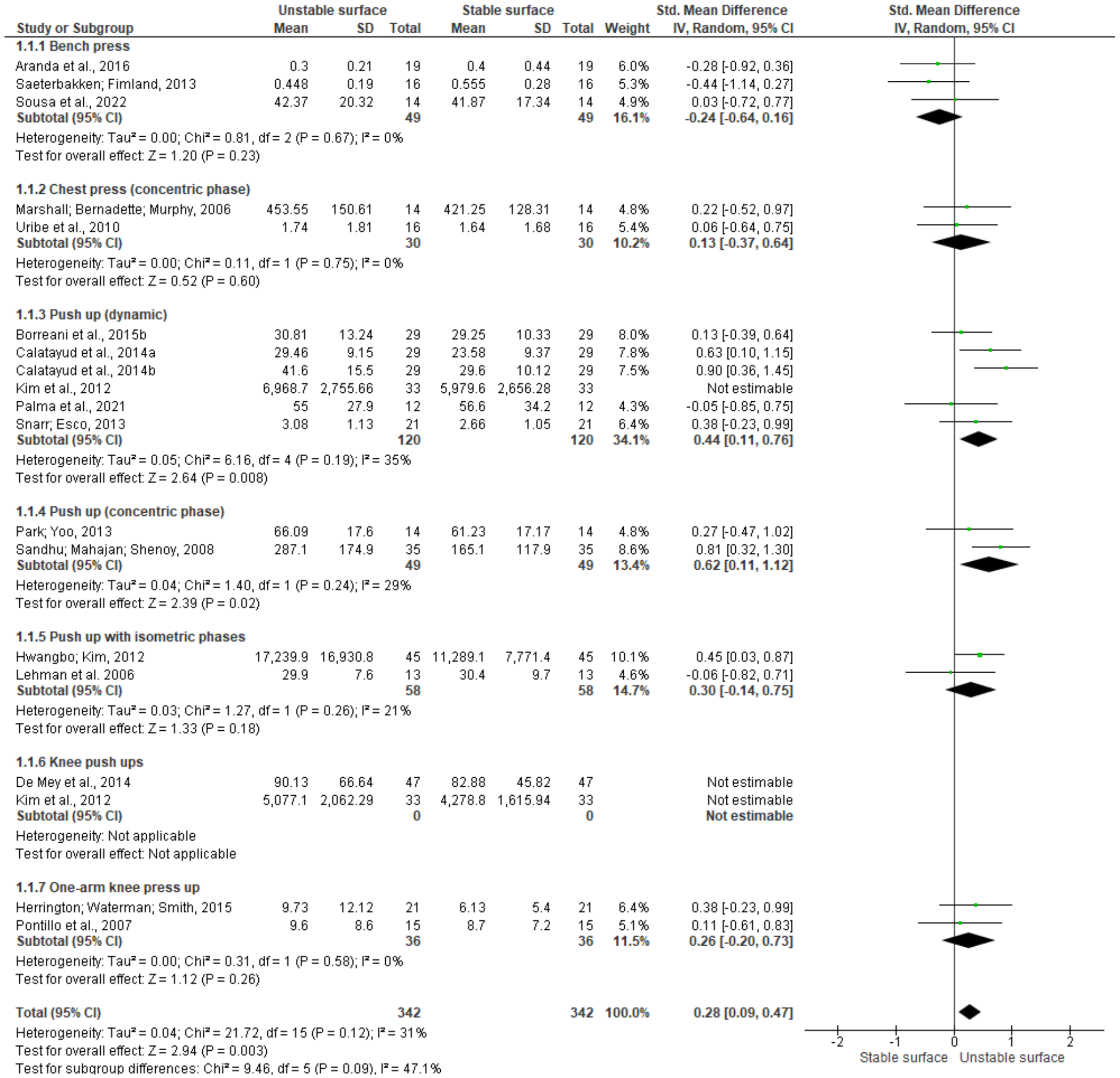

Figure 4. Forest plot of the pectoralis major muscle EMG activity on an unstable surface versus a stable surface.

Study or Subgroup	Unstable surface Mean	SD	Total	Stable surface Mean	SD	Total	Weight	Std. Mean Difference IV, Random, 95% CI
**1.3.1 Bench press**								
Saeterbakken; Fimland, 2013	0.516	0.17	16	0.747	0.23	16	0.0%	-1.11 [-1.87, -0.36]
Sousa et al., 2022	29.03	16.11	14	26.71	13.52	14	0.0%	0.15 [-0.59, 0.89]
**Subtotal (95% CI)**			**0**			**0**		**Not estimable**
Heterogeneity: Not applicable								
Test for overall effect: Not applicable								
**1.3.2 Push up (dynamic)**								
Anderson et al., 2013	63.51	29.08	15	57.48	23.51	15	7.6%	0.22 [-0.50, 0.94]
Borreani et al., 2015b	37.03	9.69	29	17.14	7.05	29		Not estimable
Calatayud et al., 2014a	32.95	18.36	29	13.99	8.29	29		Not estimable
Calatayud et al., 2014b	24.4	9.04	29	17.14	7.05	29	12.9%	0.88 [0.34, 1.42]
Kim et al., 2012	26,154.4	14,583.14	33	22,780.5	13,105.64	33		Not estimable
Park et al., 2013	67.21	12.18	16	62.05	13.03	16	8.0%	0.40 [-0.30, 1.10]
Snarr; Esco, 2013	5.11	1.97	21	3.91	1.36	21	9.9%	0.70 [0.07, 1.32]
Syed-Abdul et al., 2018	382	333	24	307	230	24	11.8%	0.26 [-0.31, 0.83]
**Subtotal (95% CI)**			**105**			**105**	**50.1%**	**0.52 [0.25, 0.80]**
Heterogeneity: Tau² = 0.00; Chi² = 3.63, df = 4 (P = 0.46); I² = 0%								
Test for overall effect: Z = 3.71 (P = 0.0002)								
**1.3.3 Push up (concentric phase)**								
Park; Yoo, 2013	75.43	11.31	14	72.29	14.07	14	7.1%	0.24 [-0.51, 0.98]
Sandhu; Mahajan; Shenoy, 2008	265.8	113.6	35	179.4	113.4	35	15.7%	0.75 [0.27, 1.24]
**Subtotal (95% CI)**			**49**			**49**	**22.8%**	**0.58 [0.10, 1.05]**
Heterogeneity: Tau² = 0.03; Chi² = 1.29, df = 1 (P = 0.26); I² = 22%								
Test for overall effect: Z = 2.36 (P = 0.02)								
**1.3.4 Push up with isometric phases**								
Hwangbo; Kim, 2012	6,080.2	3,395	45	5,077.1	2,408.2	45	20.5%	0.34 [-0.08, 0.75]
Lehman et al. 2006	29.9	10.1	13	32.7	17.5	13	6.6%	-0.19 [-0.96, 0.58]
**Subtotal (95% CI)**			**58**			**58**	**27.2%**	**0.18 [-0.30, 0.65]**
Heterogeneity: Tau² = 0.04; Chi² = 1.39, df = 1 (P = 0.24); I² = 28%								
Test for overall effect: Z = 0.73 (P = 0.46)								
**Total (95% CI)**			**212**			**212**	**100.0%**	**0.45 [0.25, 0.66]**
Heterogeneity: Tau² = 0.01; Chi² = 8.63, df = 8 (P = 0.37); I² = 7%								
Test for overall effect: Z = 4.37 (P < 0.0001)								
Test for subgroup differences: Chi² = 1.78, df = 2 (P = 0.41), I² = 0%								

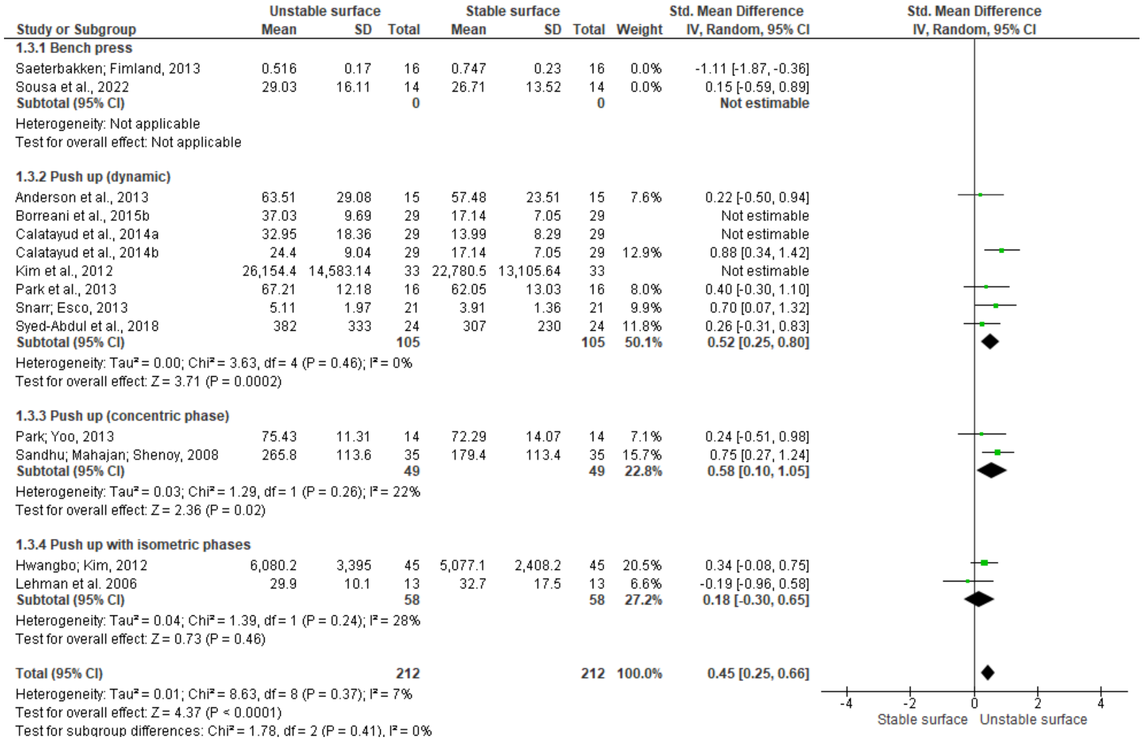

Figure 5. Forest plot of the triceps muscle EMG activity on an unstable surface versus a stable surface.

Study or Subgroup	Unstable surface Mean	SD	Total	Stable surface Mean	SD	Total	Weight	Std. Mean Difference IV, Random, 95% CI
**1.2.1 Bench press**								
Aranda et al., 2016	0.65	0.42	19	0.52	0.13	19	8.8%	0.41 [-0.23, 1.05]
Saeterbakken; Fimland, 2013	0.943	0.29	16	0.945	0.28	16	7.9%	-0.01 [-0.70, 0.69]
Sousa et al., 2022	66.58	36.21	14	68.46	33.68	14	7.1%	-0.05 [-0.79, 0.69]
**Subtotal (95% CI)**			**49**			**49**	**23.8%**	**0.14 [-0.26, 0.54]**
Heterogeneity: Tau² = 0.00; Chi² = 1.10, df = 2 (P = 0.58); I² = 0%								
Test for overall effect: Z = 0.69 (P = 0.49)								
**1.2.2 Chest press (concentric phase)**								
Marshall; Bernadette; Murphy, 2006	449.39	227.86	14	337.24	216.78	14	6.9%	0.49 [-0.26, 1.24]
Uribe et al., 2010	0.85	0.41	16	0.9	0.35	16	7.9%	-0.13 [-0.82, 0.57]
**Subtotal (95% CI)**			**30**			**30**	**14.7%**	**0.16 [-0.44, 0.77]**
Heterogeneity: Tau² = 0.05; Chi² = 1.40, df = 1 (P = 0.24); I² = 28%								
Test for overall effect: Z = 0.53 (P = 0.60)								
**1.2.3 Push up (dynamic)**								
Borreani et al. 2015a	83.74	32.64	30	78.54	24.04	30	12.5%	0.18 [-0.33, 0.69]
Borreani et al., 2015b	19.08	4.9	29	26.22	7.86	29		Not estimable
Calatayud et al., 2014a	16.42	7.59	29	29.68	13.78	29		Not estimable
Calatayud et al., 2014b	22.18	7.59	29	26.22	7.86	29	11.9%	-0.52 [-1.04, 0.01]
Snarr; Esco, 2013	5.08	1.55	21	4.01	1.27	21	9.2%	0.74 [0.11, 1.37]
Syed-Abdul et al., 2018	320	240	24	281	171	24	10.7%	0.18 [-0.38, 0.75]
**Subtotal (95% CI)**			**104**			**104**	**44.3%**	**0.13 [-0.36, 0.62]**
Heterogeneity: Tau² = 0.17; Chi² = 9.50, df = 3 (P = 0.02); I² = 68%								
Test for overall effect: Z = 0.51 (P = 0.61)								
**1.2.4 One-arm knee press up**								
Herrington; Waterman; Smith, 2015	9.9	7	21	11	7.2	21	9.7%	-0.15 [-0.76, 0.45]
Pontillo et al., 2007	10.95	11.34	15	12.58	9.28	15	7.5%	-0.15 [-0.87, 0.56]
**Subtotal (95% CI)**			**36**			**36**	**17.2%**	**-0.15 [-0.62, 0.31]**
Heterogeneity: Tau² = 0.00; Chi² = 0.00, df = 1 (P = 1.00); I² = 0%								
Test for overall effect: Z = 0.65 (P = 0.52)								
**Total (95% CI)**			**219**			**219**	**100.0%**	**0.08 [-0.14, 0.30]**
Heterogeneity: Tau² = 0.03; Chi² = 13.14, df = 10 (P = 0.22); I² = 24%								
Test for overall effect: Z = 0.70 (P = 0.48)								
Test for subgroup differences: Chi² = 1.15, df = 3 (P = 0.77), I² = 0%								

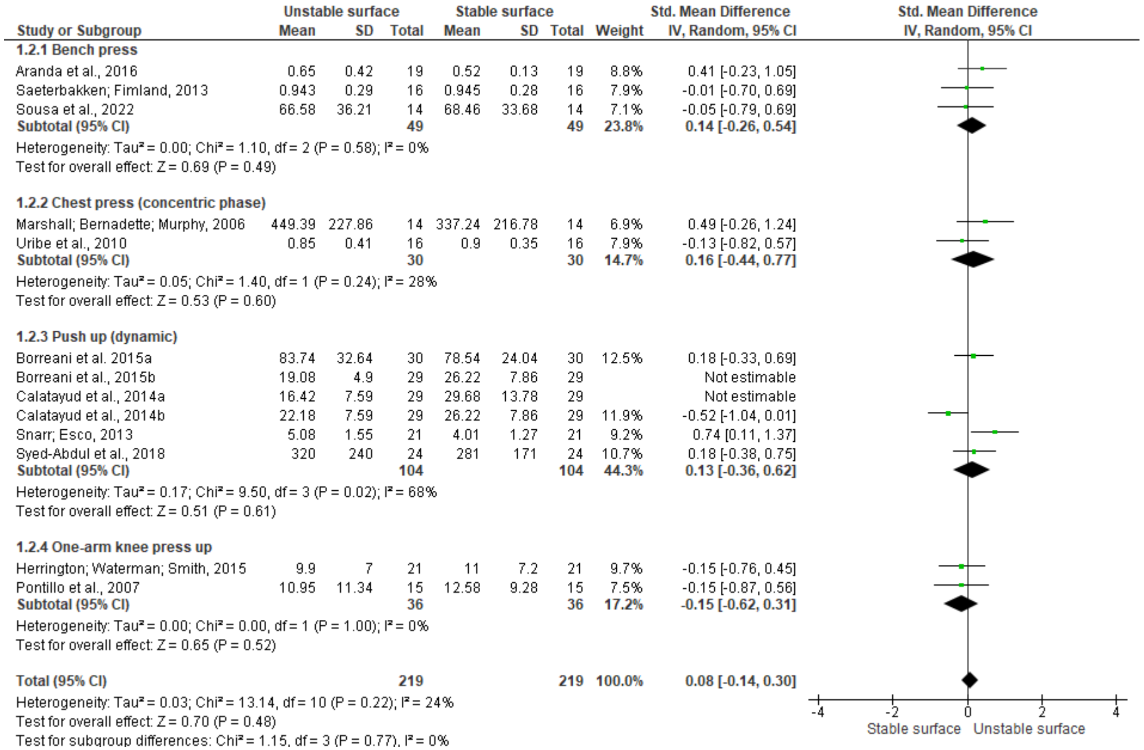

Figure 6. Forest plot of the anterior deltoid muscle EMG activity on an unstable surface versus a stable surface.

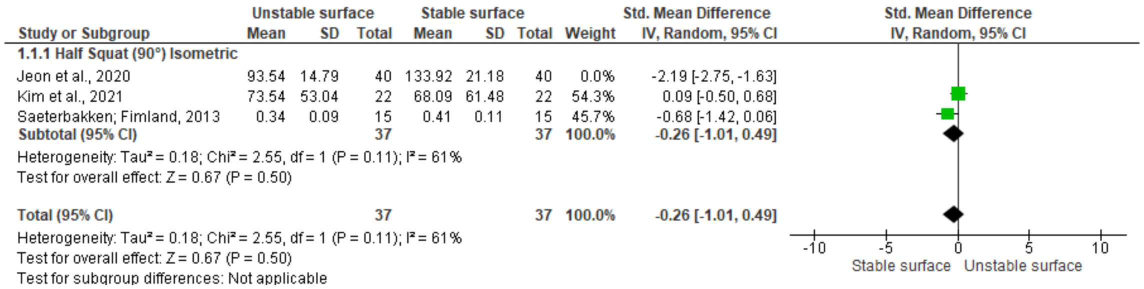

Figure 7. Forest plot of the rectus femoris muscle EMG activity on an unstable surface versus a stable surface.

Study or Subgroup	Unstable surface Mean	SD	Total	Stable surface Mean	SD	Total	Weight	Std. Mean Difference IV, Random, 95% CI
**1.2.1 Squat**								
Andersen et al., 2014	83.2	35.4	15	81.1	22.4	15	6.0%	0.07 [-0.65, 0.78]
Aranda et al.,2016	0.34	0.22	19	0.34	0.21	19	7.7%	0.00 [-0.64, 0.64]
Buscá et al,. 2020	0.74	0.26	14	0.59	0.15	14	5.3%	0.69 [-0.08, 1.45]
Gündoğan; Aydin; Sağlam, 2023	101.52	34.33	14	99.72	33.23	14	5.6%	0.05 [-0.69, 0.79]
Li; Cao; Chen, 2013	32.4	11.6	13	32	11.4	13	5.2%	0.03 [-0.74, 0.80]
**Subtotal (95% CI)**			**75**			**75**	**29.9%**	**0.15 [-0.17, 0.47]**
Heterogeneity: Tau² = 0.00; Chi² = 2.30, df = 4 (P = 0.68); I² = 0%								
Test for overall effect: Z = 0.92 (P = 0.36)								
**1.2.2 Bulgarian squat**								
Aguilera-Castells et al., 2019	76.79	16.99	20	72.34	21.51	20	8.0%	0.23 [-0.40, 0.85]
Andersen et al., 2014	77.8	24.7	15	83.7	25.7	15	6.0%	-0.23 [-0.95, 0.49]
**Subtotal (95% CI)**			**35**			**35**	**14.0%**	**0.03 [-0.44, 0.50]**
Heterogeneity: Tau² = 0.00; Chi² = 0.87, df = 1 (P = 0.35); I² = 0%								
Test for overall effect: Z = 0.13 (P = 0.90)								
**1.2.3 Half Squat (90°) Isometric**								
Jeon et al., 2020	160.01	25.3	40	88.67	14.02	40	0.0%	3.45 [2.75, 4.16]
Kim et al., 2021	53.8	20.99	22	54.67	27.24	22	8.9%	-0.04 [-0.63, 0.56]
Marshall; Murphy, 2006	23.62	10.43	12	22.68	11.15	12	4.8%	0.08 [-0.72, 0.88]
Saeterbakken; Fimland, 2013	0.44	0.12	15	0.46	0.13	15	6.0%	-0.16 [-0.87, 0.56]
**Subtotal (95% CI)**			**49**			**49**	**19.7%**	**-0.04 [-0.44, 0.35]**
Heterogeneity: Tau² = 0.00; Chi² = 0.19, df = 2 (P = 0.91); I² = 0%								
Test for overall effect: Z = 0.21 (P = 0.83)								
**1.2.4 Squat (60°) Isometric**								
Hyong; Kang, 2013	50.92	20.87	14	48.64	14.8	14	5.6%	0.12 [-0.62, 0.86]
Kang et al., 2017	159.96	25.26	24	165.15	24.7	24	9.6%	-0.20 [-0.77, 0.36]
Marín; Hazell, 2014	0.0355	0.0126	28	0.0399	0.0158	28	11.2%	-0.30 [-0.83, 0.22]
Park et al., 2015	53.49	23.15	25	45	21.83	25	9.9%	0.37 [-0.19, 0.93]
**Subtotal (95% CI)**			**91**			**91**	**36.3%**	**-0.02 [-0.34, 0.29]**
Heterogeneity: Tau² = 0.02; Chi² = 3.54, df = 3 (P = 0.32); I² = 15%								
Test for overall effect: Z = 0.15 (P = 0.88)								
**Total (95% CI)**			**250**			**250**	**100.0%**	**0.03 [-0.14, 0.21]**
Heterogeneity: Tau² = 0.00; Chi² = 7.72, df = 13 (P = 0.86); I² = 0%								
Test for overall effect: Z = 0.35 (P = 0.73)								
Test for subgroup differences: Chi² = 0.78, df = 3 (P = 0.85), I² = 0%								

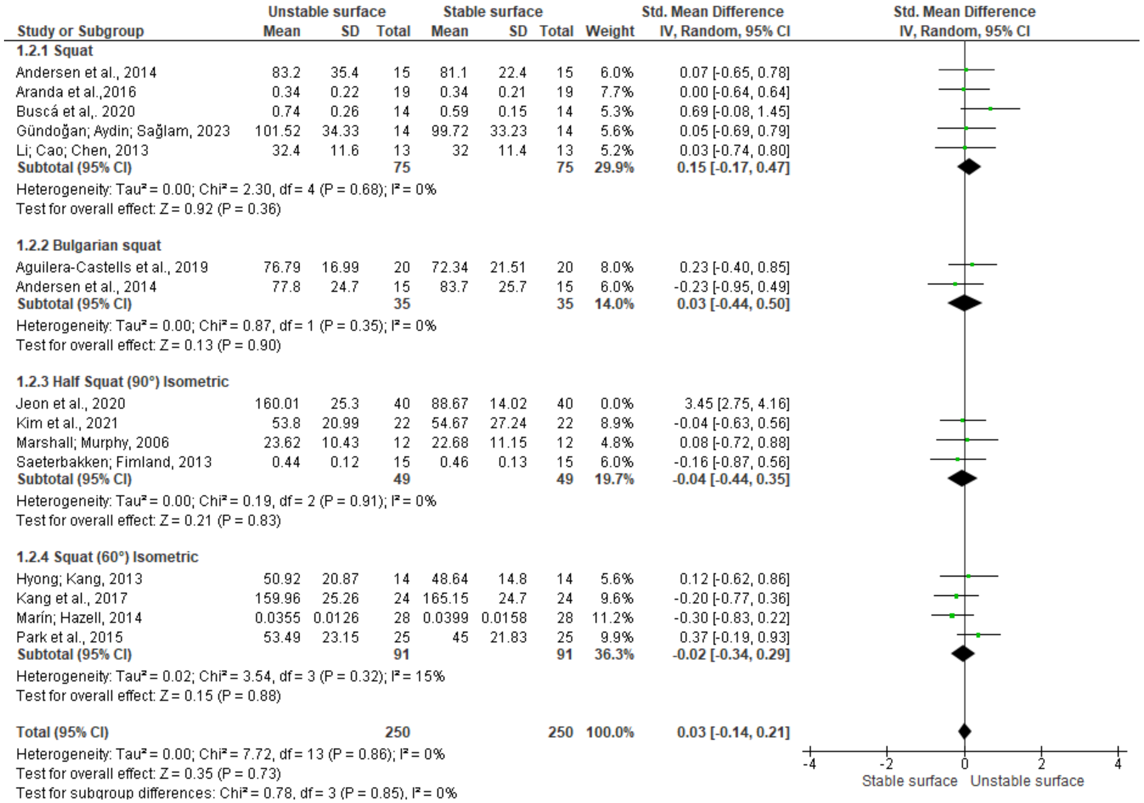

Figure 8. Forest plot of the vastus lateralis muscle EMG activity on an unstable surface versus a stable surface.

Study or Subgroup	Unstable surface Mean	SD	Total	Stable surface Mean	SD	Total	Weight	Std. Mean Difference IV, Random, 95% CI
**1.3.1 Squat**								
Buscá et al,. 2020	0.73	0.22	14	0.63	0.22	14	10.4%	0.44 [-0.31, 1.19]
Gündoğan; Aydin; Sağlam, 2023	89.16	16.74	14	84.57	19.04	14	10.6%	0.25 [-0.50, 0.99]
Li; Cao; Chen, 2013	33.7	6.5	13	32.2	8.7	13	10.1%	0.19 [-0.58, 0.96]
**Subtotal (95% CI)**			**41**			**41**	**31.1%**	**0.29 [-0.14, 0.73]**
Heterogeneity: Tau² = 0.00; Chi² = 0.23, df = 2 (P = 0.89); I² = 0%								
Test for overall effect: Z = 1.32 (P = 0.19)								
**1.3.2 Squat (60°) Isometric**								
Hyong; Kang, 2013	69.5	17.65	14	44.28	18.19	14	0.0%	1.37 [0.53, 2.20]
Kang et al., 2017	150.01	31.44	24	176.63	36.38	24	13.9%	-0.77 [-1.36, -0.18]
Marín; Hazell, 2014	0.0376	0.0125	28	0.044	0.0167	28	15.5%	-0.43 [-0.96, 0.10]
Park et al., 2015	41.21	17.92	25	35.75	17.72	25	14.7%	0.30 [-0.26, 0.86]
**Subtotal (95% CI)**			**77**			**77**	**44.1%**	**-0.30 [-0.90, 0.31]**
Heterogeneity: Tau² = 0.21; Chi² = 7.14, df = 2 (P = 0.03); I² = 72%								
Test for overall effect: Z = 0.95 (P = 0.34)								
**1.3.3 Half Squat (90°) Isometric**								
Han et al., 2017	48	13	15	48.5	12.1	15	0.0%	-0.04 [-0.75, 0.68]
Jeon et al., 2020	168.22	26.6	40	104.14	16.47	40	0.0%	2.87 [2.24, 3.50]
Kim et al., 2021	45.75	18.41	22	51.52	25.55	22	13.8%	-0.25 [-0.85, 0.34]
Saeterbakken; Fimland, 2013	0.56	0.26	15	0.67	0.51	15	11.0%	-0.26 [-0.98, 0.45]
**Subtotal (95% CI)**			**37**			**37**	**24.8%**	**-0.26 [-0.72, 0.20]**
Heterogeneity: Tau² = 0.00; Chi² = 0.00, df = 1 (P = 0.98); I² = 0%								
Test for overall effect: Z = 1.11 (P = 0.27)								
**1.3.4 Deep Squat (100°) Isometric**								
Han et al., 2017	47.3	14.7	15	48	13.6	15	0.0%	-0.05 [-0.76, 0.67]
Mcbride; Cormie; Deane, 2006	119.56	54.53	9	182.35	63.28	9	0.0%	-1.01 [-2.01, -0.02]
**Subtotal (95% CI)**			**0**			**0**		**Not estimable**
Heterogeneity: Not applicable								
Test for overall effect: Not applicable								
**Total (95% CI)**			**155**			**155**	**100.0%**	**-0.10 [-0.40, 0.20]**
Heterogeneity: Tau² = 0.08; Chi² = 12.24, df = 7 (P = 0.09); I² = 43%								
Test for overall effect: Z = 0.66 (P = 0.51)								
Test for subgroup differences: Chi² = 3.81, df = 2 (P = 0.15), I² = 47.5%								

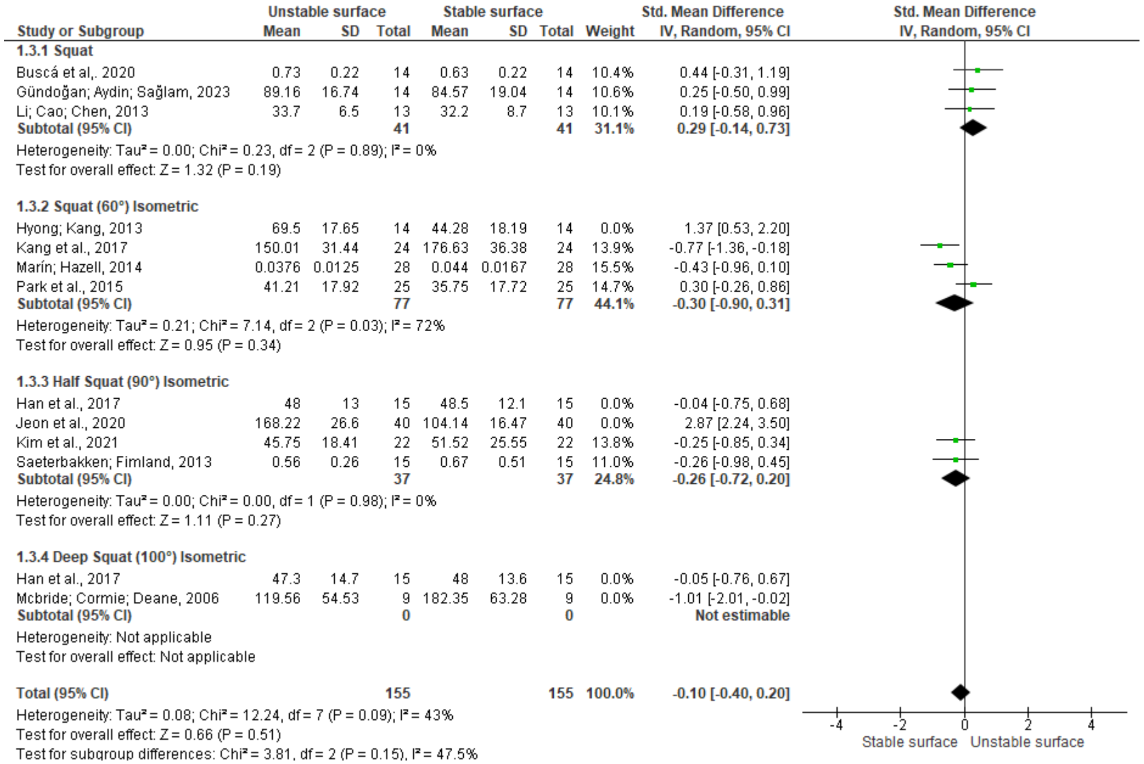

Figure 9. Forest plot of the vastus medialis muscle EMG activity on an unstable surface versus a stable surface.

Study or Subgroup	Unstable surface Mean	Unstable surface SD	Unstable surface Total	Stable surface Mean	Stable surface SD	Stable surface Total	Weight	Std. Mean Difference IV, Random, 95% CI
**1.4.1 Squat**								
Andersen et al., 2014	118.8	38.8	15	121.7	34.5	15	10.5%	-0.08 [-0.79, 0.64]
Aranda et al.,2016	0.08	0.06	19	0.09	0.08	19	13.2%	-0.14 [-0.78, 0.50]
Buscá et al,. 2020	0.33	0.11	14	0.23	0.11	14	8.8%	0.88 [0.10, 1.66]
Li; Cao; Chen, 2013	18.4	12	13	17.1	10.6	13	9.1%	0.11 [-0.66, 0.88]
**Subtotal (95% CI)**			**61**			**61**	**41.6%**	**0.16 [-0.28, 0.61]**
Heterogeneity: Tau² = 0.07; Chi² = 4.56, df = 3 (P = 0.21); I² = 34%								
Test for overall effect: Z = 0.72 (P = 0.47)								
**1.4.2 Bulgarian squat**								
Aguilera-Castells et al., 2019	27.21	9.88	20	24.5	10.73	20	13.9%	0.26 [-0.37, 0.88]
Andersen et al., 2014	193.3	101.5	15	215.5	106.7	15	10.4%	-0.21 [-0.93, 0.51]
**Subtotal (95% CI)**			**35**			**35**	**24.3%**	**0.06 [-0.41, 0.53]**
Heterogeneity: Tau² = 0.00; Chi² = 0.92, df = 1 (P = 0.34); I² = 0%								
Test for overall effect: Z = 0.24 (P = 0.81)								
**1.4.3 Half Squat (90°) Isometric**								
Han et al., 2017	45.7	30.5	15	39	37.9	15	0.0%	0.19 [-0.53, 0.91]
Kim et al., 2021	40.47	21.27	22	43.32	36.6	22	15.4%	-0.09 [-0.68, 0.50]
Marshall; Murphy, 2006	10.54	8	12	9.81	6.99	12	8.4%	0.09 [-0.71, 0.89]
Saeterbakken; Fimland, 2013	0.11	0.05	15	0.1	0.04	15	10.4%	0.21 [-0.50, 0.93]
**Subtotal (95% CI)**			**49**			**49**	**34.1%**	**0.05 [-0.35, 0.44]**
Heterogeneity: Tau² = 0.00; Chi² = 0.44, df = 2 (P = 0.80); I² = 0%								
Test for overall effect: Z = 0.23 (P = 0.82)								
**1.4.4 Deep Squat (100°) Isometric**								
Han et al., 2017	42.9	22.7	15	41.7	37.6	15	0.0%	0.04 [-0.68, 0.75]
Mcbride; Cormie; Deane, 2006	57.61	33.19	9	38.9	23.71	9	0.0%	0.62 [-0.33, 1.57]
**Subtotal (95% CI)**			**0**			**0**		**Not estimable**
Heterogeneity: Not applicable								
Test for overall effect: Not applicable								
**Total (95% CI)**			**145**			**145**	**100.0%**	**0.09 [-0.14, 0.32]**
Heterogeneity: Tau² = 0.00; Chi² = 6.08, df = 8 (P = 0.64); I² = 0%								
Test for overall effect: Z = 0.77 (P = 0.44)								
Test for subgroup differences: Chi² = 0.17, df = 2 (P = 0.92), I² = 0%								

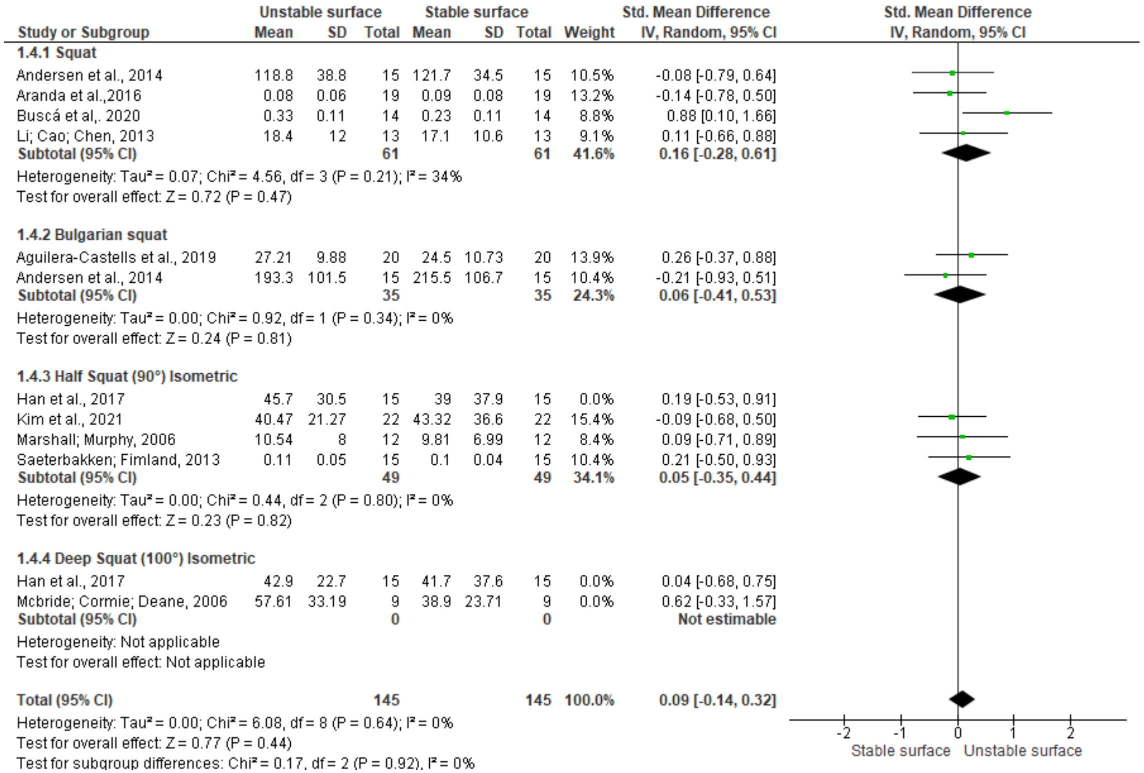

Figure 10. Forest plot of the biceps femoris muscle EMG activity on an unstable surface versus a stable surface.
